# ELAVL1-mediated USP29 mRNA degradation activates TAK1 driving M1 microglial polarization and neural stem cell differentiation dysregulation in spinal cord injury

**DOI:** 10.1038/s41420-025-02604-8

**Published:** 2025-07-09

**Authors:** Chunhe Sha, Feng Pan, Xiaodong Liu, Zhiqing Wang, Guohui Liu, Kai Huang

**Affiliations:** Department of Orthopaedics, Shanghai Jing’an District Zhabei Central Hospital, Shanghai, China

**Keywords:** Stem cells, Diseases

## Abstract

Spinal cord injury (SCI) represents a profound neurological condition characterized by motor dysfunction and sensory impairment. Microglial polarization significantly influences neurorepair and regeneration post SCI. This study aims to investigate the regulatory role of the ELAV-like RNA binding protein 1 (ELAVL1)-ubiquitin-specific peptidase 29 (USP29)-transforming growth factor beta-activated kinase 1 (TAK1) axis in microglial polarization and its effects on differentiation of neural stem cells (NSCs). A rat model of SCI was established via spinal cord transection at the tenth thoracic vertebra segment, followed by short hairpin RNA (shRNA) lentivirus infection. Motor function and coordination were evaluated while histopathological analysis of spinal cord tissues was conducted. Microglial polarization and NSC differentiation were assessed via immunofluorescence and Western blot analysis. In cellular experiments, lipopolysaccharide (LPS) was utilized to induce M1 polarization in HMC3 cells, with polarization status determined by flow cytometry, immunofluorescence, and WB. Co-immunoprecipitation, GST pull-down, and ubiquitination assays elucidated USP29 effects on TAK1 ubiquitination and activation. In SCI rat spinal cord tissues and LPS-treated HMC3 cells, we observed upregulation of ELAVL1 and phosphorylated level of TAK1, while USP29 expression was downregulated. ELAVL1 was found to bind USP29 mRNA, promoting its degradation and suppressing USP29 expression. USP29 directly interacted with TAK1, inhibiting its ubiquitination and phosphorylation. Knockdown of ELAVL1 significantly enhanced USP29 mRNA stability, inhibited TAK1 activation, promoted M2 microglial polarization, and suppressed M1 polarization. In vivo downregulation of ELAVL1 promoted the differentiation of NSCs into neurons by inhibiting M1 polarization and promoting M2 polarization, thereby improving motor function, alleviating nerve injury, and facilitating spinal cord repair. ELAVL1 exacerbates SCI pathology by degrading USP29 mRNA, thereby activating TAK1 and driving M1 microglial polarization. Targeting the ELAVL1-USP29-TAK1 axis may offer therapeutic potential for enhancing neurorepair in SCI.

**Figure:**
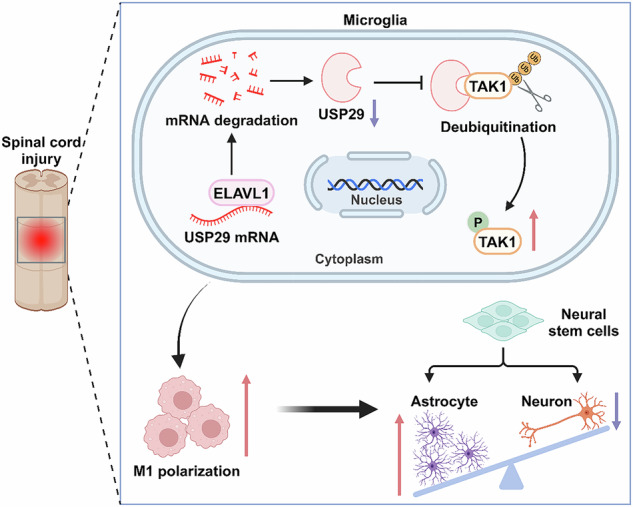
Schematic diagram of the ELAVL1-USP29-TAK1 axis mediating M1 microglial polarization and NSC differentiation dysregulation exacerbating SCI.

Schematic diagram of the ELAVL1-USP29-TAK1 axis mediating M1 microglial polarization and NSC differentiation dysregulation exacerbating SCI.

## Introduction

Spinal cord injury (SCI) is a devastating neurological injury that typically results in the loss of motor, sensory, and autonomic functions [1]. Globally, approximately 200,000 to 500,000 individuals sustain SCI annually, with incidence rates steadily rising [1, 2]. SCI not only directly causes a loss of bodily functions but also severely impacts psychological health and quality of life, placing a heavy burden on families, society, and the economy [3, 4]. Current interventions including surgical decompression, pharmacological therapy, and rehabilitation may offer symptomatic relief but lack curative efficacy [5]. Consequently, identifying novel therapeutic strategies remains a critical focus of SCI research.

Microglial polarization and neural stem cell (NSC) differentiation are crucial for neurorepair and functional recovery post SCI. Microglia adopt two primary phenotypes: the pro-inflammatory M1 subtype, which exacerbates neuronal damage via pro-inflammatory cytokine release [6–8], and the anti-inflammatory M2 subtype, which promotes neurorepair and regeneration [9]. Following SCI, the injury microenvironment predominantly drives M1 polarization, while promoting M2 polarization has been shown to enhance recovery [10, 11]. Concurrently, the disrupted SCI microenvironment, which is characterized by oxidative stress, inflammation, microcirculatory dysfunction, and axonal disruption, could impede NSC differentiation into neurons and favor astrocytic differentiation [12]. Promoting the NSC neuronal differentiation while reversing their differentiation into astrocytes is a promising therapeutic avenue for SCI[13, 14]. Notably, M1 microglia facilitate the differentiation of NSCs into astrocytes, whereas M2 microglia support the differentiation of NSCs into neurons [15], suggesting that microglial polarization modulates NSC fate and SCI outcomes.

ELAV like RNA binding protein 1 (ELAVL1), also known as HuR, is an RNA-binding protein that regulates mRNA stability and translation by binding to 3’untranslated regions (UTRs), influencing diverse biological processes [16]. In SCI, the expression of ELAVL1 is significantly increased [17], and its inhibition can effectively suppress M1 microglial polarization and enhance motor function in SCI mice [18]. In addition, ubiquitin-specific peptidase 29 (USP29) is an important deubiquitinase that modulates protein stability by removing ubiquitin chains [19, 20]. USP29 stabilizes NRF2 through deubiquitination, promoting M2 microglial polarization and ameliorating SCI [21]. Transforming growth factor β-activated kinase 1 (TAK1) is a serine/threonine kinase that regulates microglial polarization; its inhibition can downregulate the MAPK signaling, reduce microglial activation and neuronal damage following SCI [22]. On the other hand, TAK1 activation promotes M1 microglial polarization through the NF-κB pathway [23]. Although ELAVL1, USP29, and TAK1 individually influence microglial behavior, their interrelationships and collective role in SCI remain undefined.

This study aims to elucidate the regulatory mechanisms of ELAVL1, USP29, and TAK1 in microglial polarization and their effects on NSC differentiation. Through systematic cellular and animal experiments, we seek to clarify how the ELAVL1-USP29-TAK1 axis regulates microglial polarization and differentiation of NSCs, thus offering new insights and potential targets for SCI management and treatment.

## Results

### Downregulation of USP29 may be involved in M1 microglial polarization following SCI

M1 microglial polarization is closely related to the occurrence and progression of SCI [24]. To further explore the regulatory mechanisms of microglial polarization in SCI, we obtained the GSE245470 dataset from the Gene Expression Omnibus (GEO) database. DEGs were identified using thresholds of |log_2_(fold change)| > 1 and *P*.adj < 0.05, resulting in a total of 4358 DEGs. Among them, 2579 DEGs were upregulated and 1779 DEGs were downregulated in SCI (Fig. S1A). Considering the crucial role of deubiquitinating enzymes (DUBs) in microglial phenotype transformation [25, 26], we screened 95 DUBs from the UbiBrowser 2.0 database and combined them with SCI- and microglial polarization-related genes from GeneCards. Venn diagram revealed 4 overlapping DEGs (Fig. S1B). Further screening using machine learning algorithms identified 3 feature genes via LASSO regression (Fig. S1C) and 1 feature gene through random forest analysis (Fig. S1D). Finally, the intersection of these analyses identified USP29 as the core candidate gene (Fig. S1E). USP29 exhibited significantly reduced expression in the GSE245470 dataset (Fig. S1F).

**Fig. 1:**
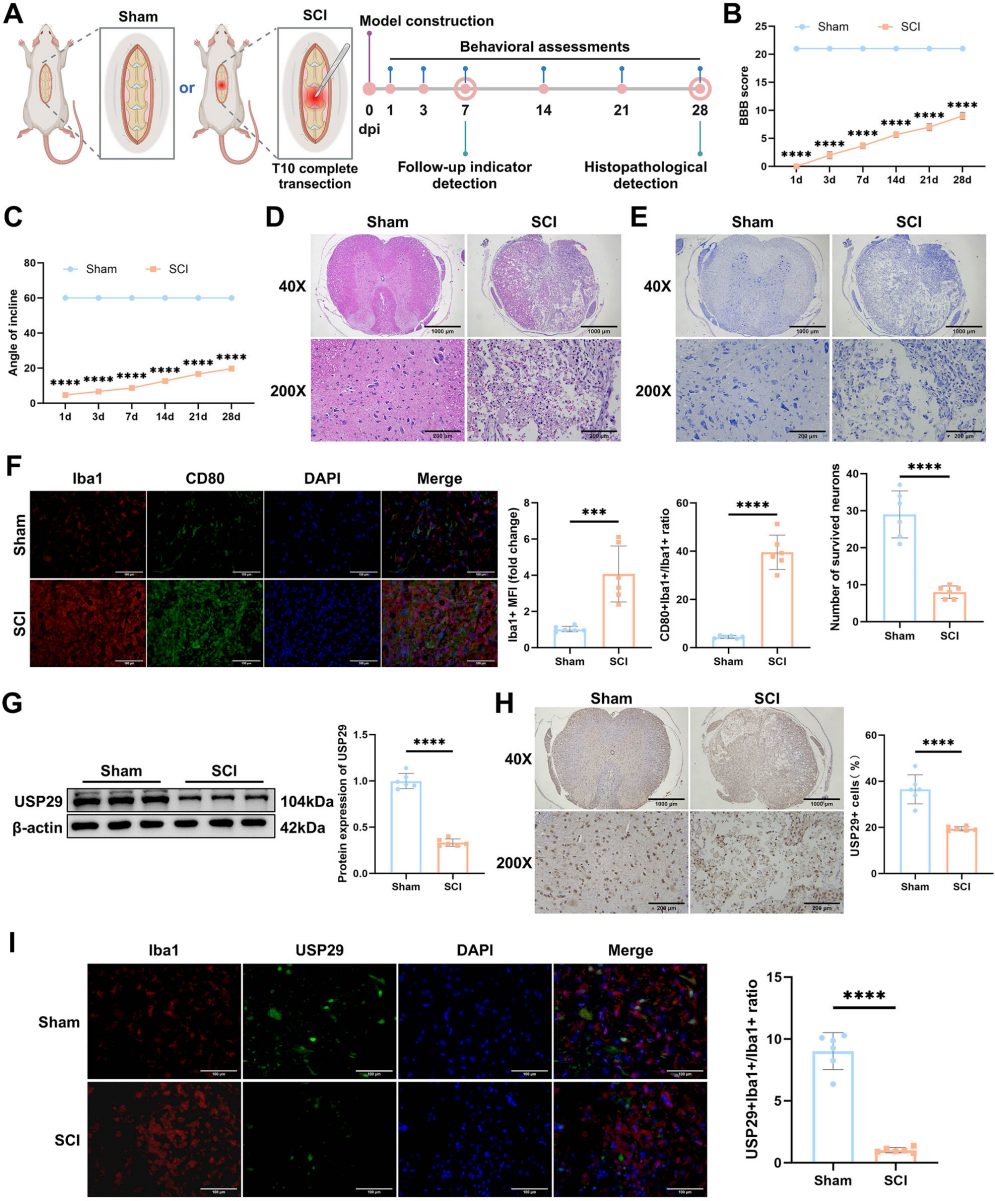
The impact of SCI on microglial polarization and USP29 expression in spinal cord tissues. **A** Schematic diagram of the SCI rat model construction. **B** BBB scores of rats in each group at days 1, 3, 7, 14, 21, and 28 post-SCI for motor function assessment. **C** Inclined plane test scores of rats in each group at days 1, 3, 7, 14, 21, and 28 post-SCI, used to assess motor coordination. **D** Representative images of HE staining of spinal cord tissue on 28 d in each group (scale bars: 1000 μm and 200 μm). **E** Representative images of Nissl staining of spinal cord tissue on 28 d in each group (scale bars: 1000 μm and 200 μm) and the quantification of surviving neurons. **F** Representative images of Iba1 and CD80 IF staining on 7 d (scale bar = 100 μm), and quantification of average fluorescence intensity of Iba1+ cells, CD80+ cells and related fluorescence intensity ratio. **G** Western blot analysis and protein quantification of USP29 expression in spinal cord tissue of each group on 7 d. **H** Representative images of USP29 IHC staining in spinal cord tissue on 7 d (scale bars: 1000 μm and 200 μm), and quantification of the percentage of USP29-positive cells. **I** Representative images of Iba1 and USP29 IF staining on 7 d (scale bar = 100 μm) as well as quantification of USP29 +Iba1+/Iba1+ fluorescence intensity ratio. *N* = 6, *** indicates *p* < 0.001, **** indicates *p* < 0.0001.

To further verify the changes in USP29 expression, we established a rat model of SCI (Fig. 1A). The BBB scores and inclined plane test results demonstrated a significant decline in motor function and coordination in the SCI group (Fig. 1B, C). HE and Nissl staining revealed substantial tissue damage, hemorrhage, and edema in the spinal cord, along with a reduced number of surviving neurons in the SCI group (Fig. 1D, E). These findings confirm the successful establishment of the SCI model. IF analysis results showed a marked increase in the fluorescence intensity of Iba1+ and CD80+ cells (Fig. 1F), indicating microglial activation and promotion of M1 polarization following SCI. Consistent with expectations, SCI downregulated the expression of USP29 in the spinal cord tissues (Fig. 1G, H). Additionally, compared with the Sham group, the fluorescence intensity of USP29 in Iba1^+^ microglia was significantly curtailed in the SCI group, further suggesting that SCI inhibits USP29 expression in microglia (Fig. 1I). Collectively, these results suggest that the downregulation of the deubiquitinase USP29 may play a critical role in M1 microglial polarization following SCI.

### USP29 suppresses LPS-induced M1 microglial polarization

**Fig. 2:**
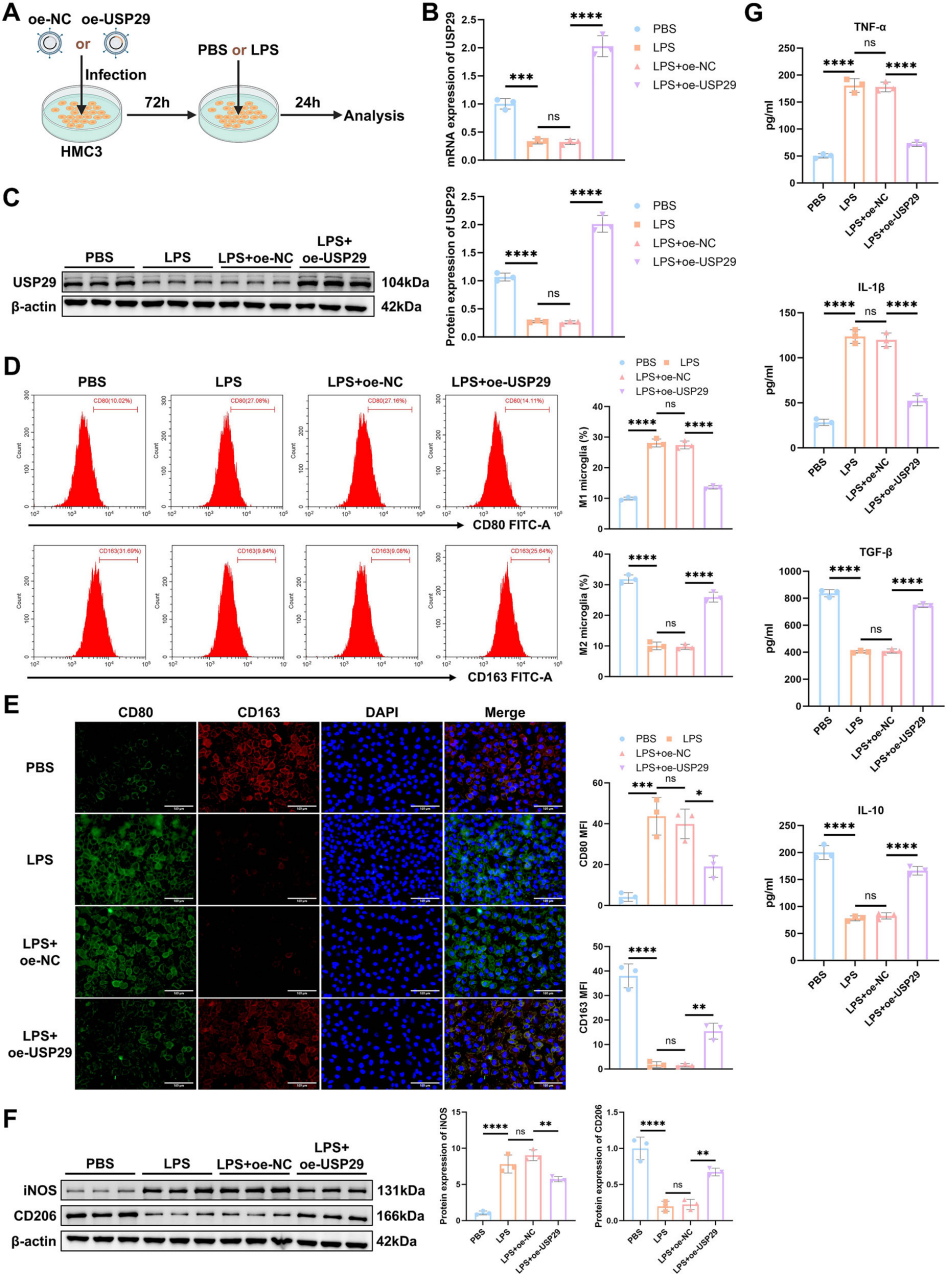
Effect of USP29 on LPS-induced polarization of HMC3 cells. **A** Schematic diagram of lentiviral infection and LPS treatment of HMC3 cells. **B** RT-qPCR detection of USP29 mRNA expression levels in HMC3 cells. **C** Western blot detection of USP29 protein expression in HMC3 cells. **D** Flow cytometry detection of the percentage of M1 and M2 microglia in each group of cells. **E** Representative IF images of CD80 and CD163 in cells from each group (scale bar = 100 μm), and quantification of average fluorescence intensity. **F** Western blot detection of iNOS and CD206 protein expression in cells. **G** ELISA detection of the levels of pro-inflammatory cytokines and anti-inflammatory cytokines in cell culture supernatants. All cell experiments were repeated three times, ns indicates *p* > 0.05, * indicates *p* < 0.05, ** indicates *p* < 0.01, *** indicates *p* < 0.001, **** indicates *p* < 0.0001.

To clarify the regulatory role of USP29 in microglial polarization, human microglial HMC3 cells were infected with lentivirus of oe-USP29. Following that, cells were treated with LPS to induce microglial M1 polarization (Fig. 2A). As anticipated, LPS treatment significantly inhibited the expression of USP29 in HMC3 cells. Concurrently, we successfully generated a USP29 overexpression model (Fig. 2B, C). Flow cytometry results showed that LPS treatment increased the proportion of M1 microglia while significantly decreasing the proportion of M2 microglia. However, overexpression of USP29 reversed the LPS-induced M1 microglial polarization (Fig. 2D). This finding was further corroborated by IF staining and Western blot analysis (Fig. 2E, F). Additionally, ELISA results showed that LPS treatment promoted the secretion of pro-inflammatory cytokines (TNF-α and IL-1β) and significantly reduced the levels of anti-inflammatory cytokines (TGF-β and IL-10). In contrast, USP29 overexpression by transfection with oe-USP29 effectively counteracted the LPS-induced alterations in cytokine secretion (Fig. 2G). Meanwhile, compared with the PBS + oe-NC group, overexpression of USP29 in the PBS + oe-USP29 group significantly inhibited microglial M1 polarization and promoted their polarization toward the M2 phenotype, further confirming that USP29 possessed the ability to regulate microglial polarization even in the absence of inflammatory stimulation (Fig. S2A–F).

In summary, overexpression of USP29 promotes M2 microglial polarization and inhibits M1 polarization, effectively ameliorating LPS-induced microglial polarization imbalance. These results highlight the regulatory role of USP29 in maintaining the balance of microglial polarization.

### USP29 inhibits TAK1 phosphorylation by deubiquitination

**Fig. 3:**
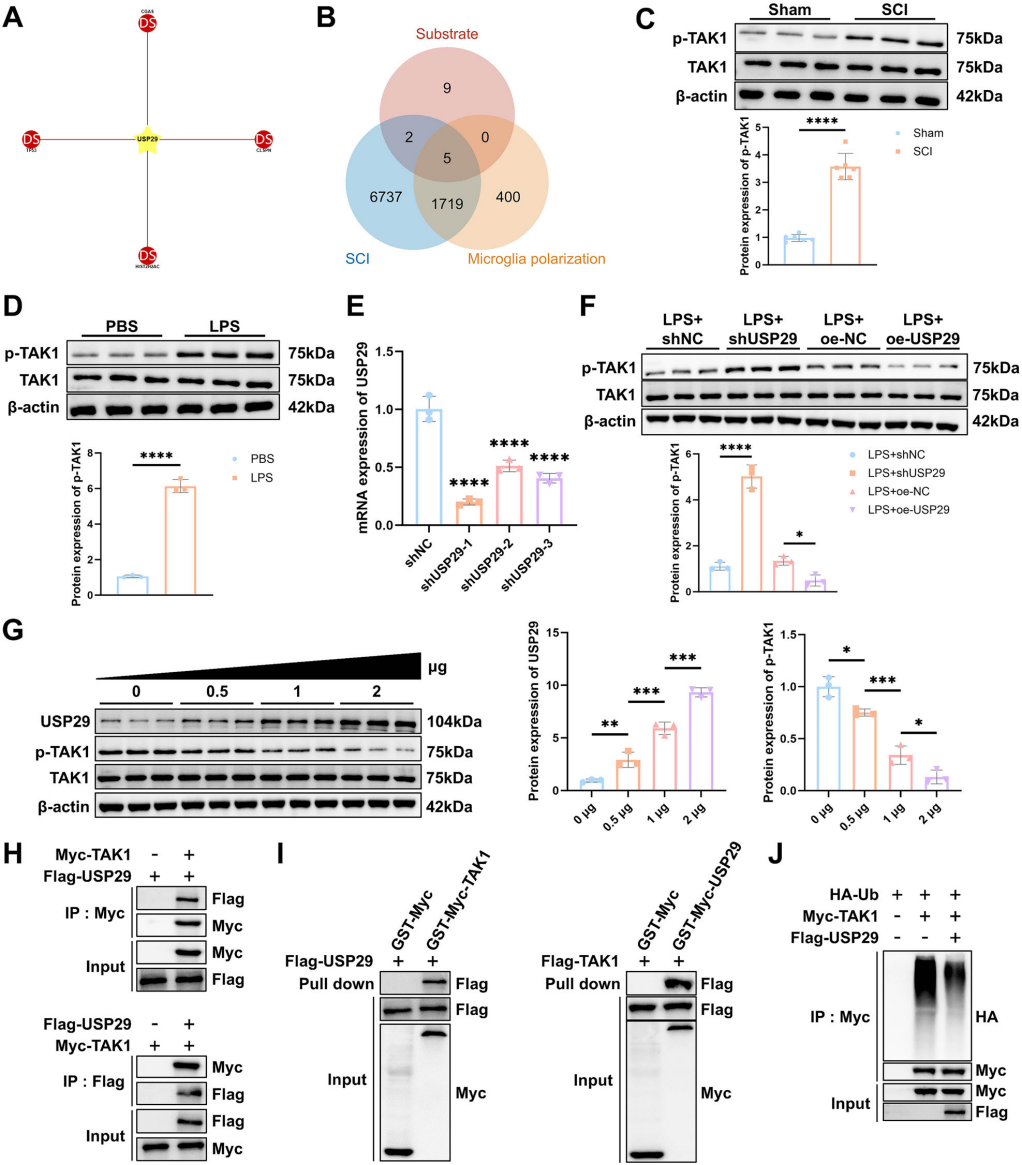
The effect of USP29 on TAK1 ubiquitination and phosphorylation. **A** Network diagram of USP29 and known deubiquitination substrates from the UbiBrowser 2.0 database. **B** Venn diagram of USP29 substrates and genes related to SCI and microglial polarization. **C** Western blot analysis of p-TAK1 and TAK1 protein expression in spinal cord tissues of rats on day 7 after SCI (*N* = 6). **D** Western blot analysis of p-TAK1 and TAK1 protein expression in HMC3 cells of each group. **E** RT-qPCR analysis of USP29 expression in HMC3 cells. **F** Western blot analysis of p-TAK1 and TAK1 protein expression in HMC3 cells of each group. **G** Western blot analysis of USP29, p-TAK1, and TAK1 protein expression in HEK293T cells transfected with different doses of oe-USP29 plasmids (0, 0.5, 1, or 2 μg) for 48 h. **H** CO-IP analysis of USP29 interaction with TAK1 in HEK293T cells co-transfected with Flag-USP29 and Myc-TAK1 plasmids. **I** GST pull-down experiment assessing the direct interaction between USP29 and TAK1 in HEK293T cells, with GST-Myc plasmid as a control. **J** After transfecting HEK293T cells with Myc-TAK1, Flag-USP29, and HA-Ub plasmids for 48 h, immunoprecipitation was performed using anti-Myc agarose beads, followed by Western blotting with anti-HA and anti-Myc antibodies. *p < 0.05, **p < 0.01, ***p < 0.001, ****p < 0.0001. All cell experiments were repeated three times.

To elucidate the specific mechanisms regulated by USP29, we first screened 4 known USP29 deubiquitination substrates from the UbiBrowser 2.0 database (Fig. 3A), and supplemented the list with 12 additional substrates through literature search. Venn analysis of these 16 substrates and genes related to SCI and microglia polarization, resulting in 5 intersection substrates: TP53, CGAS, HIF1A, TWIST1, and TAK1 (Fig. 3B). Among these, phosphorylation of TAK1 is closely related to microglial polarization [23]. We further observed a distinct increase in TAK1 phosphorylation in SCI rat spinal cord tissues and LPS-treated HMC3 cells (Fig. 3C, D). which suggests that USP29 may regulate microglial polarization by modulating TAK1 activity.

To verify this hypothesis, we utilized shUSP29 lentivirus to knock down USP29 expression (Fig. 3E), selecting the most effective shUSP29-1 construct for further experiments. We then assessed the impact of USP29 on TAK1 phosphorylation. Western blot analysis revealed that silencing USP29 in LPS-treated HMC3 cells enhanced TAK1 phosphorylation, while USP29 overexpression suppressed TAK1 phosphorylation (Fig. 3F). These results indicate that downregulated USP29 promotes TAK1 activation during the LPS-induced M1 microglial polarization.

To further elucidate the mechanism by which USP29 regulates TAK1 activation, we transfected HEK293T cells with different doses of USP29 overexpression plasmids (oe-USP29). The results showed that oe-USP29 upregulated USP29 expression and inhibited TAK1 phosphorylation in a dose-dependent manner (Fig. 3G). Next, Co-IP experiments revealed a direct interaction between USP29 and TAK1 (Fig. 3H), which was further confirmed by GST pull-down assays (Fig. 3I). IP analysis of TAK1 ubiquitination levels demonstrated that Flag-USP29 transfection significantly reduced TAK1 ubiquitination compared to the Myc-TAK1 and HA-Ub co-transfection group (Fig. 3J). These findings collectively suggest that USP29 directly interacts with TAK1 and deubiquitinates it, thereby inhibiting TAK1 phosphorylation.

### USP29 inhibits TAK1 activation to promote M2 microglial polarization

**Fig. 4:**
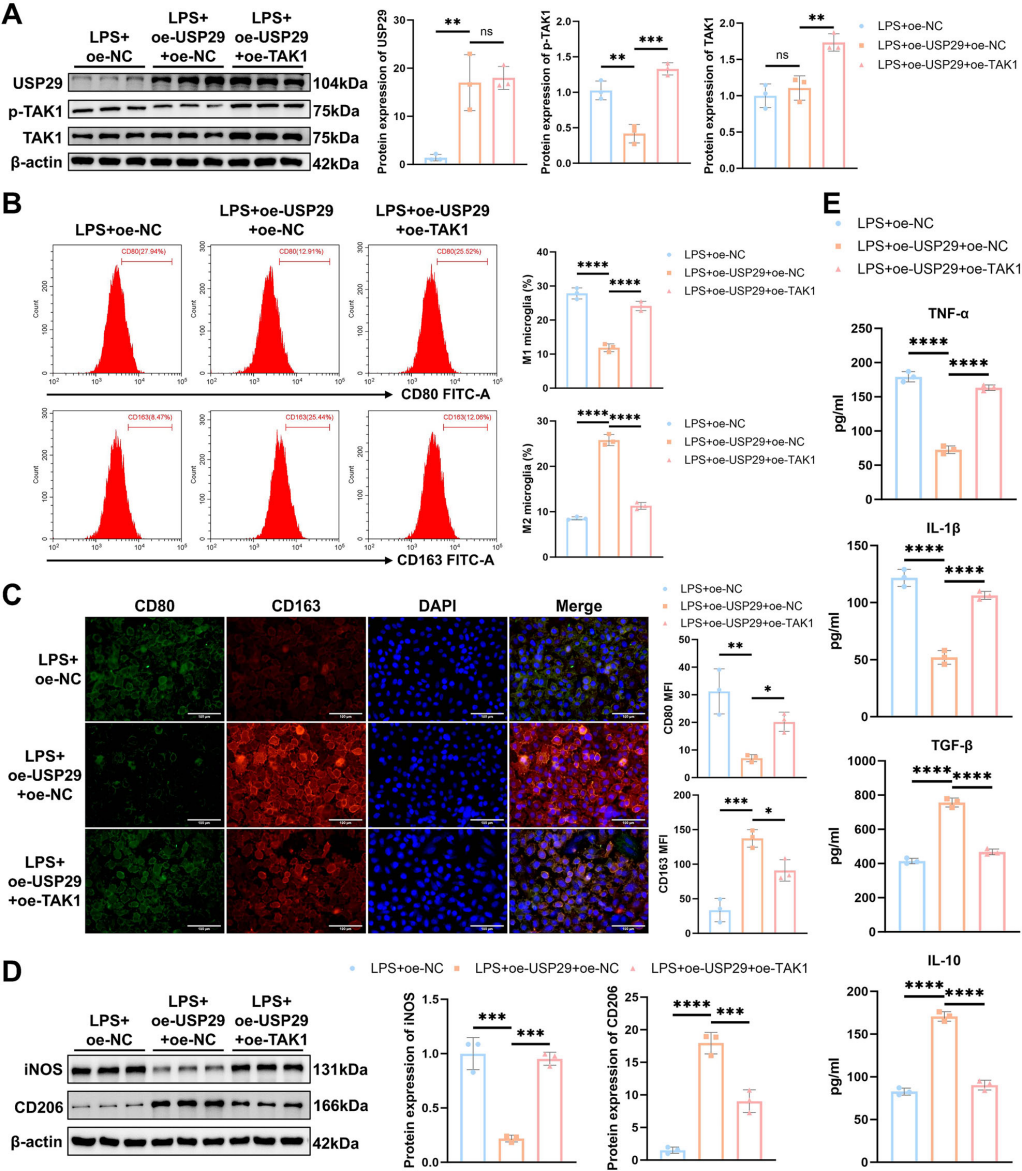
The effect of USP29-regulated TAK1 on HMC3 cell polarization. **A** Western blot detection of USP29, p-TAK1, and TAK1 protein expression in HMC3 cells. **B** Flow cytometry analysis of the percentage of M1 and M2 microglia in each group of cells. **C** Representative images of CD80 and CD163 IF staining in each group of cells (scale bar = 100 μm) and quantification of the average fluorescence intensity. **D** Western blot analysis of iNOS and CD206 protein expression in each group of cells. **E** ELISA detection of pro-inflammatory and anti-inflammatory cytokine levels in cell culture supernatants. All cell experiments were repeated 3 times, ns indicates *p* > 0.05, * indicates *p* < 0.05, ** indicates *p* < 0.01, *** indicates *p* < 0.001, **** indicates *p* < 0.0001.

Next, we further investigated whether USP29 influences microglial polarization through TAK1. We verified the co-transfection efficiency of lentiviruses overexpressing USP29 (oe-USP29) and TAK1 (oe-TAK1). Western blot analysis showed that, compared with the oe-NC group, USP29 expression was significantly upregulated in the oe-USP29 + oe-NC group, accompanied by a marked decrease in p-TAK1 levels. Meanwhile, oe-TAK1 treatment significantly increased the expression of TAK1 and p-TAK1 level, but had no significant effect on USP29 expression (Fig. S3A). Under LPS stimulation, consistent trends in protein expression were observed across the oe-NC, oe-USP29 + oe-NC, and oe-USP29 + oe-TAK1 groups (Fig. 4A). Notably, CCK-8 assay results indicated that lentiviral co-infection did not significantly affect cell viability (Fig. S3B).

Flow cytometry, IF staining, and Western blot analysis showed that overexpression of USP29 significantly increased the proportion of M2 microglia and the expression of M2 polarization markers (CD163 and CD206), while decreasing the proportion of M1 microglia and the expression of M1 polarization markers (CD80 and iNOS) (Fig. 4B–D). Additionally, overexpression of USP29 significantly increased the levels of anti-inflammatory cytokines TGF-β and IL-10, while reducing the levels of pro-inflammatory cytokines TNF-α and IL-1β (Fig. 4E). However, in the LPS + oe-USP29 + oe-TAK1 group, activation of TAK1 reversed the effects of USP29 overexpression (Fig. 4B–E). Taken above, these results indicate that USP29 promotes microglial polarization toward the M2 phenotype by inhibiting TAK1 activation.

### ELAVL1 promotes USP29 mRNA degradation

**Fig. 5:**
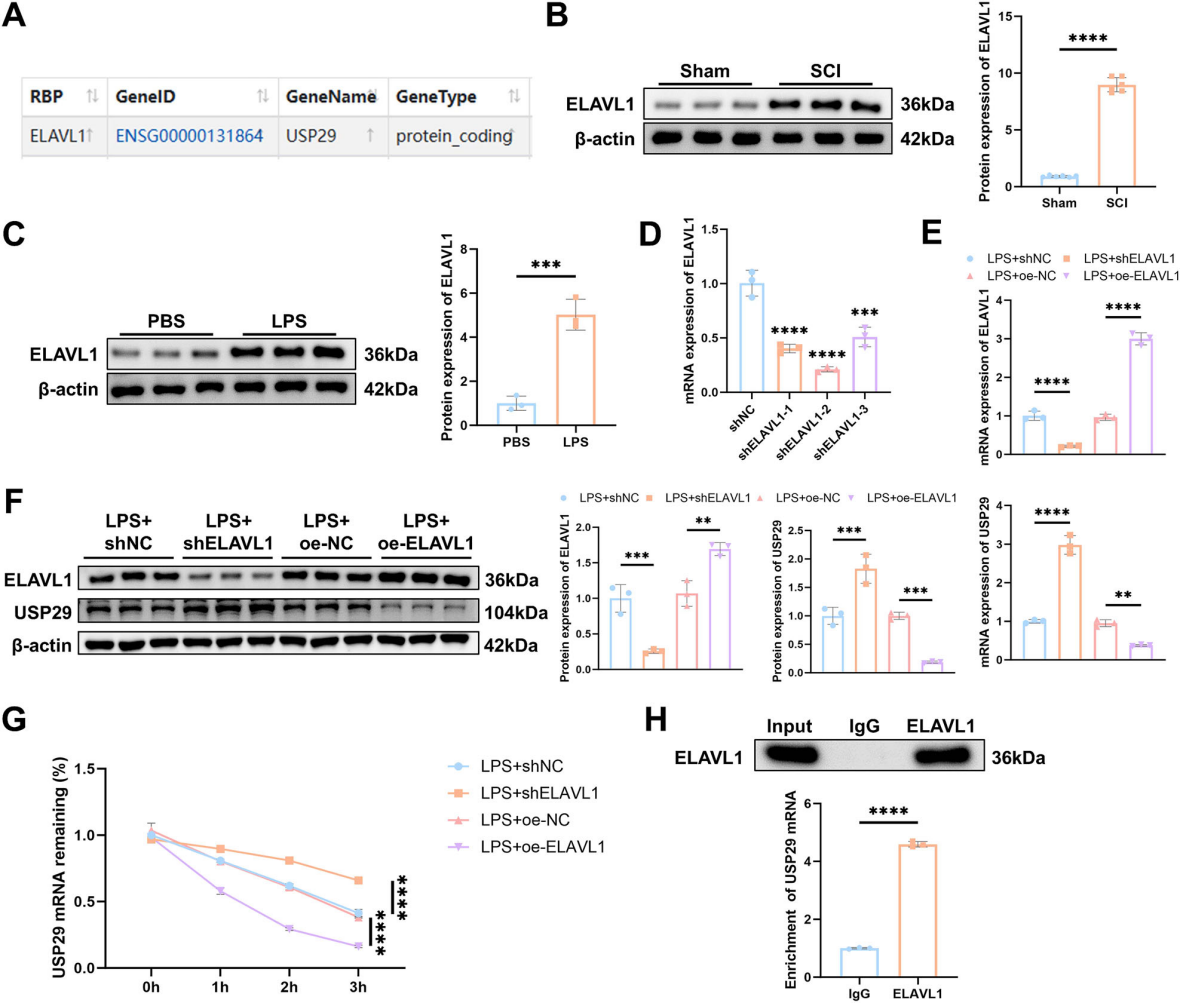
Effects of ELAVL1 on USP29 mRNA. **A** Prediction of the RNA-binding protein (RBP) that binds to USP29 mRNA through the ENCORI database. **B** Western blot analysis of ELAVL1 protein expression in spinal cord tissues of rats (*N* = 6) on 7 d. **C** Western blot analysis of ELAVL1 protein expression in cells of each group. **D** RT-qPCR analysis of ELAVL1 expression in cells of each group. **E** RT-qPCR analysis of ELAVL1 and USP29 mRNA expression in cells of each group. **F** Western blot analysis of ELAVL1 and USP29 protein expression in cells of each group. **G** RT-qPCR analysis of USP29 mRNA expression in cells at different time points (0, 1, 2, 3 h) after treatment with Actinomycin D. **H** RIP-qPCR analysis of the interaction between ELAVL1 and USP29 mRNA, with Western blot validation of the RIP experiment and RT-qPCR detection of the level of USP29 mRNA enriched by ELAVL1. ** indicates *p* < 0.01, *** indicates *p* < 0.001, **** indicates *p* < 0.0001. All cell experiments were repeated three times.

To further elucidate the specific mechanisms regulating USP29, we predicted a potential RNA-binding protein (RBP) that may regulate USP29 using the ENCORI website: ELAVL1 (HuR) (Fig. 5A). Western blot results showed that ELAVL1 expression was upregulated in both SCI rat spinal cord tissues and LPS-treated HMC3 cells (Fig. 5B, C). To investigate the regulatory role of ELAVL1 on USP29, HMC3 cells were transfected with ELAVL1 knockdown lentivirus (shELAVL1), and the knockdown efficiency was verified by RT-qPCR (Fig. 5D). The shELAVL1-2 construct with the most significant knockdown efficiency was selected for subsequent experiments.

RT-qPCR and Western blot analysis showed that silencing of ELAVL1 led to an upregulation of USP29 expression, while overexpression of ELAVL1 notably inhibited USP29 expression (Fig. 5E, F). Additionally, actinomycin D assays indicated that knockdown of ELAVL1 enhanced the stability of USP29 mRNA, while overexpression of ELAVL1 decreased its stability (Fig. 5G). Furthermore, RIP-qPCR experiments were conducted to assess the interaction between ELAVL1 and USP29 mRNA, which revealed that ELAVL1 significantly enriched USP29 mRNA compared to the IgG control, indicating a direct interaction between ELAVL1 and USP29 mRNA (Fig. 5H). In summary, these results suggest that ELAVL1 regulates USP29 expression by binding to USP29 mRNA and promoting its degradation, thereby inhibiting USP29 expression.

### Inhibition of the ELAVL1-USP29-TAK1 axis rescues LPS-induced imbalance in microglial polarization

**Fig. 6:**
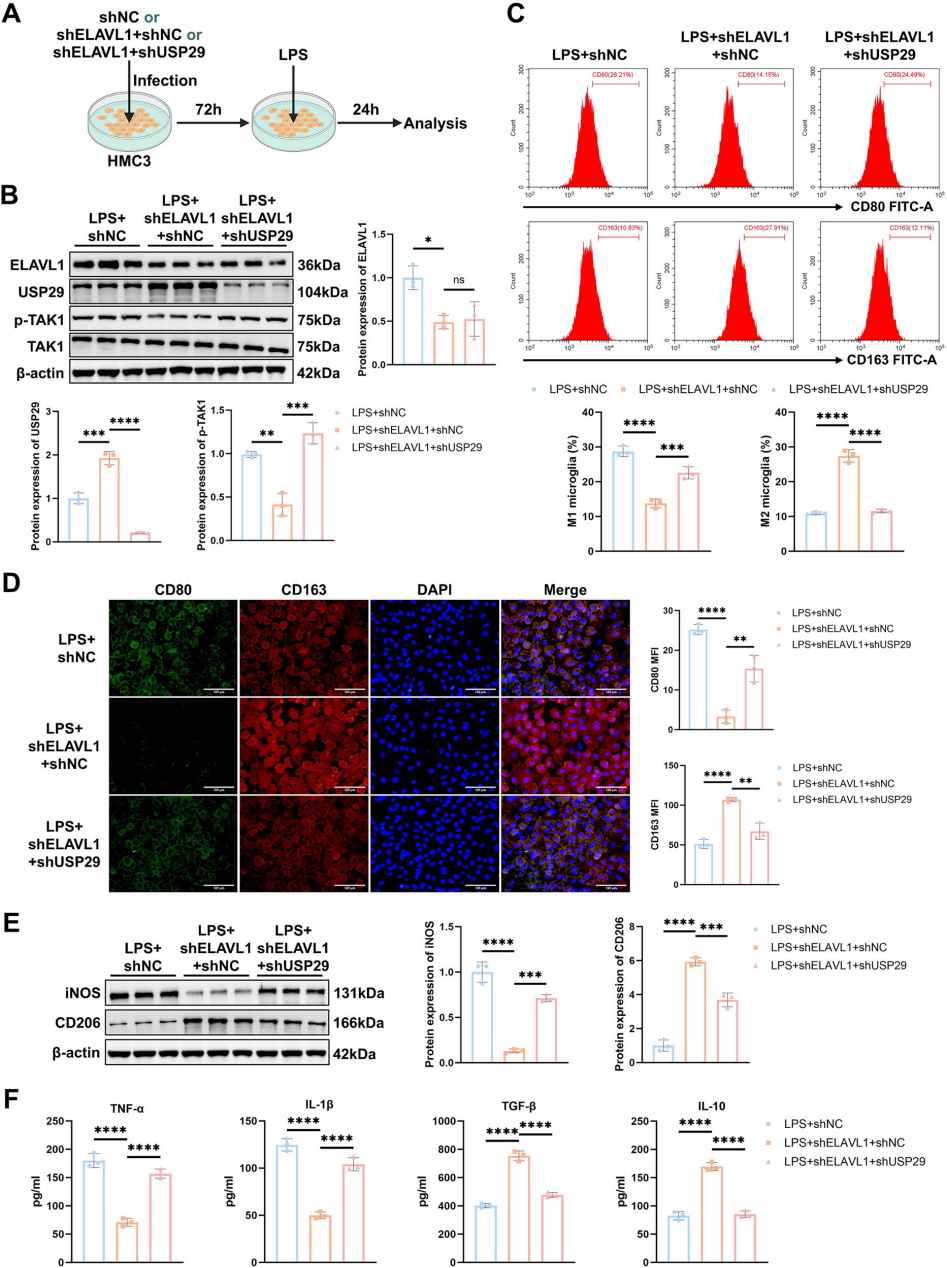
The effect of the ELAVL1-USP29-TAK1 axis on HMC3 cells polarization. **A** Diagram of HMC3 cells treated with LPS after infection with shELAVL1 and shUSP29 lentivirus. **B** Western blot detection of the protein expression of ELAVL1, USP29, p-TAK1, and TAK1 in HMC3 cells. **C** Flow cytometry detection of the percentage of M1 and M2 microglial cells in each group. **D** Representative images of CD80 and CD163 IF staining in each group (scale bar = 100 μm) and quantification of the average fluorescence intensity. **E** Western blot detection of iNOS and CD206 protein expression in each group. **F** ELISA measurement of pro-inflammatory and anti-inflammatory cytokine levels in the cell culture supernatant. All cell experiments were performed in triplicate, ns indicates *p* > 0.05; * *p* < 0.05; ** *p* < 0.01; *** *p* < 0.001; **** *p* < 0.0001.

To further validate the regulatory effect of ELAVL1 on the USP29-TAK1 axis, HMC3 cells were co-infected with shELAVL1 and shUSP29 lentiviruses, followed by LPS-induced polarization (Fig. 6A). We confirmed the infection efficiency of the lentiviruses by western blot. The results showed that shELAVL1 significantly downregulated ELAVL1 expression, accompanied by an upregulation of USP29. In contrast, co-transfection with shUSP29 markedly suppressed USP29 expression, yet without affecting ELAVL1 expression (Fig. S3C). CCK-8 assay indicated no significant differences in cell viability among the groups, ruling out effects of lentiviral infection on cell survival (Fig. S3D). Further Western blot analysis revealed that, compared to the LPS + shNC group, knockdown of ELAVL1 in the LPS + shELAVL1 + shNC group significantly increased USP29 expression and inhibited TAK1 phosphorylation. However, simultaneous knockdown of USP29 restored p-TAK1 levels without altering ELAVL1 expression (Fig. 6B). These findings confirm that ELAVL1 promotes TAK1 activation by accelerating the USP29 mRNA degradation.

Next, we explored the effect of the ELAVL1-USP29-TAK1 axis on microglial polarization. The results showed that compared to the LPS + shNC group, treatment with shELAVL1 significantly reduced the percentage of M1 microglia and the expression of M1 polarization markers (CD80 and iNOS), while significantly increasing the percentage of M2 microglia and the expression of M2 polarization markers (CD163 and CD206) (Fig. 6C–E). These results indicate that ELAVL1 knockdown promotes M2 polarization while inhibiting M1 polarization, thereby correcting the LPS-induced M1 polarization bias in microglia. However, in the LPS + shELAVL1 + shUSP29 group, silencing of USP29 inhibited M2 polarization and promoted M1 polarization (Fig. 6C–E). Additionally, silencing of ELAVL1 significantly reduced the levels of pro-inflammatory cytokines TNF-α and IL-1β, and increased the levels of anti-inflammatory cytokines TGF-β and IL-10, while knockdown of USP29 reversed the effects of shELAVL1 (Fig. 6F). Collectivley, inhibition of ELAVL1 enhances USP29 mRNA stability, suppresses TAK1 activation, and promotes the shift from M1 to M2 microglial polarization, thereby effectively rescuing the LPS-induced imbalance in microglial polarization.

### ELAVL1-USP29-TAK1 axis mediates microglial polarization imbalance, affecting NSC differentiation

**Fig. 7:**
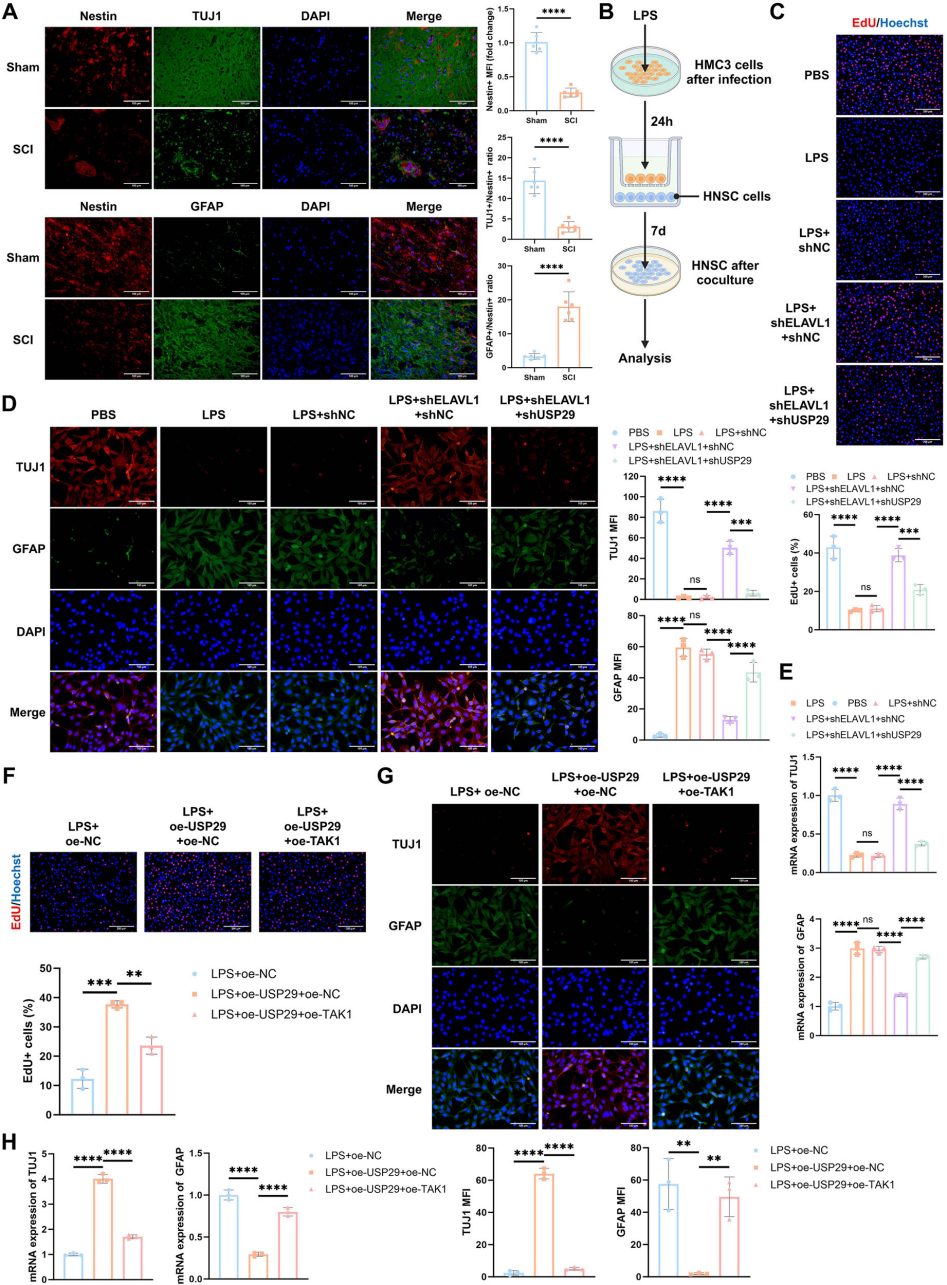
Impact of HMC3 cells polarization on the fate differentiation of HNSCs. **A** Representative IF images of Nestin and TUJ1, and Nestin and GFAP co-staining in spinal cord tissue on 7 d (scale bar = 100 μm), and quantification of the average fluorescence intensity of Nestin+ cells as well as the TUJ1+/Nestin+ or GFAP+/Nestin+ fluorescence intensity ratio, *N* = 6. **B** Diagram showing the co-culture of HMC3 cells and HNSCs. **C** EdU staining to detect HNSCs proliferation in each group, scale bar = 200 μm. **D** Representative IF images of TUJ1 and GFAP in HNSCs from each group (scale bar = 100 μm), and quantification of the average fluorescence intensity of TUJ1 and GFAP. **E** RT-qPCR measurement of TUJ1 and GFAP mRNA expression in HNSCs from each group. **F** EdU staining to detect HNSCs proliferation in each group (scale bar = 200 μm). **G** Representative IF images of TUJ1 and GFAP in HNSCs from each group (scale bar = 100 μm), and quantification of the average fluorescence intensity of TUJ1 and GFAP. **H** RT-qPCR of TUJ1 and GFAP mRNA expression in HNSCs from each group. All cell experiments were repeated 3 times, ns indicates *p* > 0.05, ** indicates *p* < 0.01, *** indicates *p* < 0.001, **** indicates *p* < 0.0001.

We evaluated the differentiation of neural stem cells (NSCs) in the spinal cord tissue of rats from different groups using IF staining. Compared to the Sham group, the SCI group showed a marked reduction in the expression of the NSC marker Nestin, a significant decrease in the number of TUJ1+ neurons, and an increase in GFAP+ astrocytes (Fig. 7A). These findings suggest that SCI induces a loss of NSCs in spinal cord tissue, promoting their differentiation into astrocytes while inhibiting neuronal differentiation.

Previous research has shown that microglial polarization plays a role in regulating NSC differentiation [27]. To further verify the effect of the ELAVL1-USP29-TAK1 axis-mediated microglial polarization and its impact on NSC differentiation, we co-cultured HMC3 cells with HNSCs under different treatments (Fig. 7B). EdU staining results indicated that LPS-treated microglia significantly inhibited the proliferation of HNSCs. Compared to the LPS + shNC group, microglia treated with LPS + shELAVL1 + shNC promoted HNSCs proliferation, whereas treatment with LPS + shELAVL1 + shUSP29 reversed this effect (Fig. 7C). Furthermore, IF staining revealed that LPS treatment increased the expression of the astrocyte marker GFAP and decreased the expression of the neuronal marker TUJ1. Knockdown of ELAVL1 in microglia reversed the impact of LPS on HNSC fate differentiation. However, knockdown of USP29 prevented this reversal (Fig. 7D), which was corroborated by RT-qPCR results (Fig. 7E). In line with the previous results, LPS + oe-USP29 + oe-NC-treated microglia significantly promoted HNSC proliferation, increased TUJ1 expression, and reduced GFAP expression. However, co-culturing HNSCs with LPS + oe-USP29 + oe-TAK1-treated microglia effectively negated these effects (Fig. 7F–H). These results suggest that LPS-induced M1 microglial polarization inhibits NSC proliferation, promotes their differentiation into astrocytes, and suppresses neuronal differentiation. Conversely, inhibiting the ELAVL1-USP29-TAK1 axis in microglia to induce M2 polarization can effectively restore proper NSC differentiation.

### ELAVL1 silencing inhibits M1 microglial polarization via the USP29-TAK1 axis, promotes neuronal differentiation of NSCs, and facilitates SCI repair

**Fig. 8:**
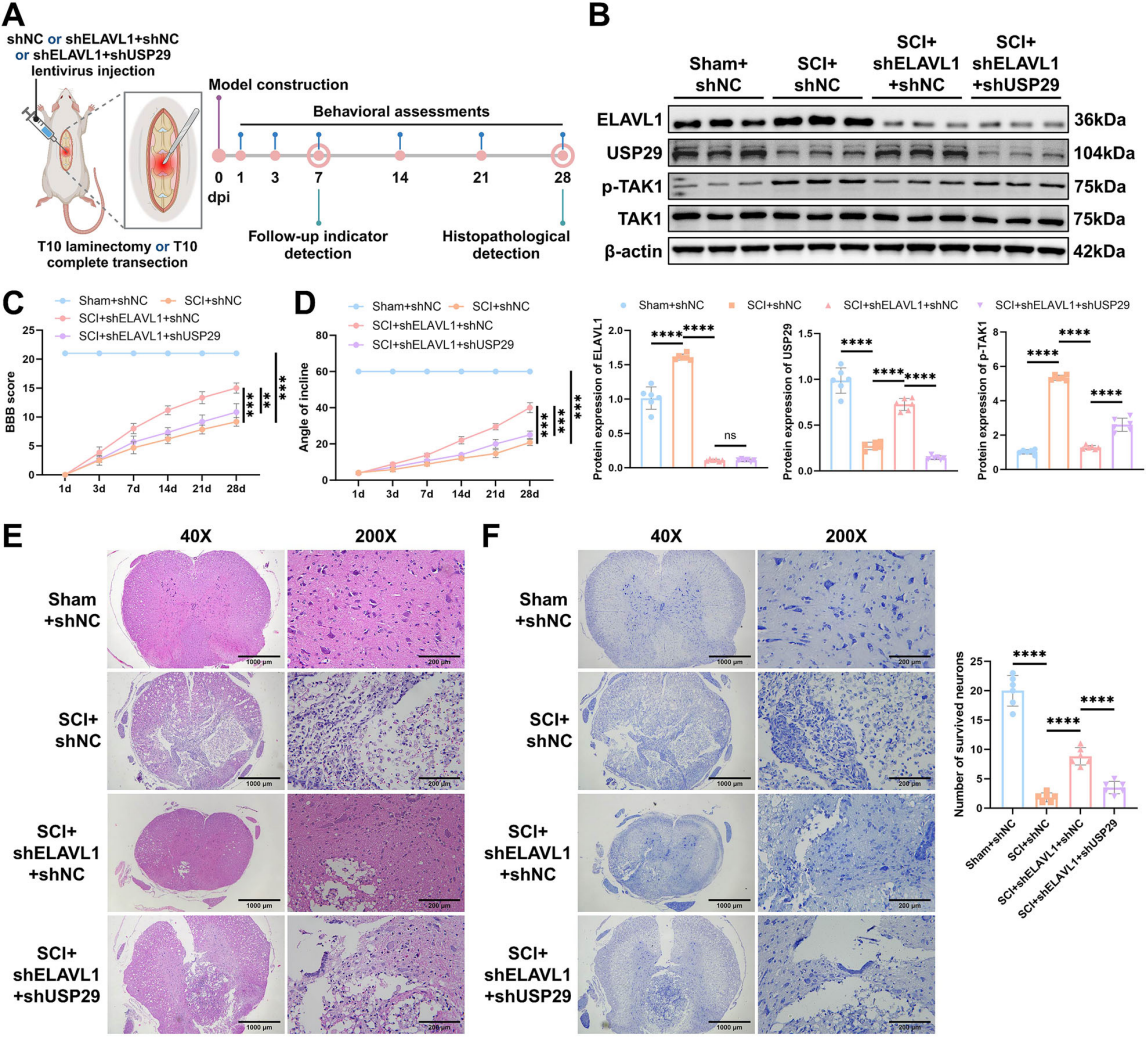
Effect of the ELAVL1-USP29-TAK1 axis on SCI rats. **A** Diagram of SCI rat model construction and lentiviral infection. **B** Western blot analysis of the expression of ELAVL1, USP29, p-TAK1, and TAK1 in spinal cord tissue on 7 d. **C** BBB scores of rats from each group on days 1, 3, 7, 14, 21, and 28 after SCI, used to assess motor function. **D** Incline test scores of rats from each group on days 1, 3, 7, 14, 21, and 28 after SCI, used to assess motor coordination. **E** Representative images of HE staining of spinal cord tissue from rats in each group on 28 d (scale bars = 1000 μm and 200 μm, respectively). **F** Representative images of Nissl staining of spinal cord tissue from rats in each group on 28 d (scale bars = 1000 μm and 200 μm, respectively) and quantification of surviving neurons. *N* = 6, ns indicates *p* > 0.05, ** indicates *p* < 0.01, *** indicates *p* < 0.001, **** indicates *p* < 0.0001.

To investigate whether the ELAVL1-USP29-TAK1 axis regulates NSC differentiation dysregulation by modulating microglial polarization in vivo, we injected lentivirus carrying shELAVL1, shUSP29, or shNC into the spinal cord of SCI rats (Fig. 8A). Western blot and IF analyses revealed that, compared to the Sham + shNC group, the SCI + shNC group exhibited significantly elevated expression of ELAVL1 and p-TAK1 level in rat spinal cord tissues and microglia, accompanied by a notable reduction in USP29 expression. Silencing ELAVL1 in the SCI + shELAVL1 + shNC group led to a significant increase in USP29 expression and a marked reduction in p-TAK1 levels in rat spinal cord tissues and microglia. Further silencing of USP29 in the SCI + shELAVL1 + shUSP29 group increased p-TAK1 level, without causing significantly altered expression of ELAVL1 (Figs. 8B and S4A–C).

Behavioral assessments showed that the BBB score and angle of incline in the SCI + shNC group significantly decreased, while silencing ELAVL1 significantly improved motor function and coordination in the SCI rats. However, silencing USP29 blocked the recovery effect induced by shELAVL1 on motor function (Fig. 8C, D). HE and Nissl staining revealed significant tissue damage, hemorrhage, and edema in the spinal cord of the rats in the SCI + shNC group, along with a marked reduction in the number of surviving neurons. Treatment with shELAVL1 significantly ameliorated the pathological changes, while USP29 silencing counteracted the therapeutic effect of shELAVL1 (Fig. 8E, F).

**Fig. 9:**
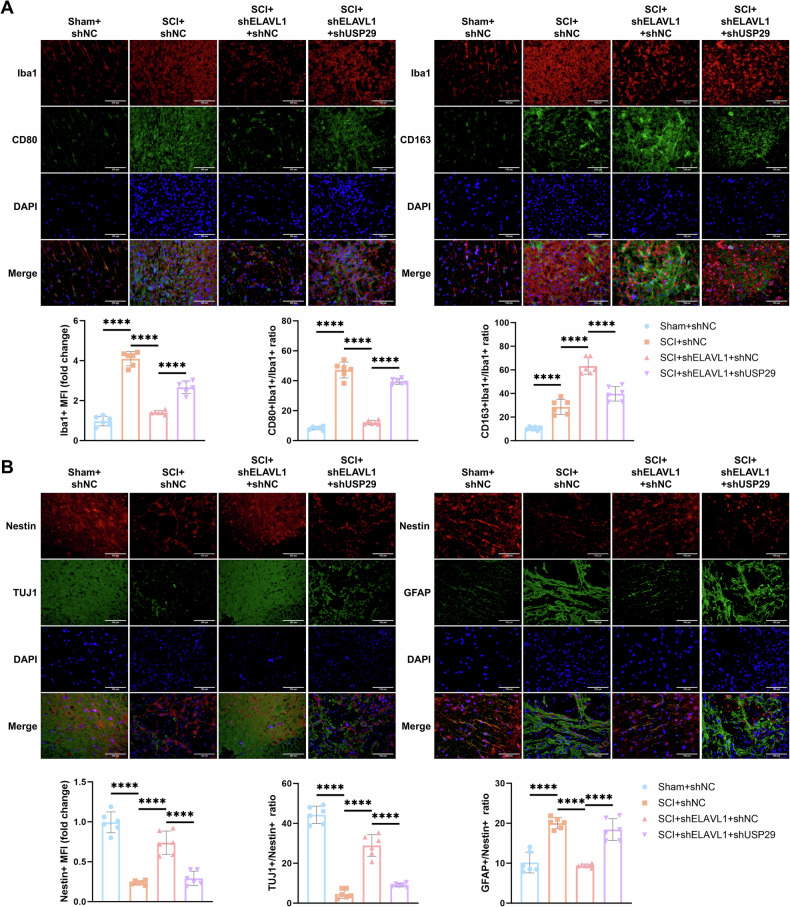
Effect of the ELAVL1-USP29-TAK1 axis on microglial polarization and NSCs differentiation in SCI rats. **A** Representative images of Iba1 and CD80, Iba1 and CD163 IF staining in spinal cord tissue on day 7 after SCI (scale bar = 100 μm), and quantification of Iba1 + fluorescence intensity and the ratio of Iba1+CD80+/Iba1 + or Iba1+CD163+/Iba1 + fluorescence intensity. **B** Representative images of Nestin and TUJ1, Nestin and GFAP IF staining in spinal cord tissue on day 7 after SCI (scale bar = 100 μm), and quantification of Nestin + fluorescence intensity and the ratio of TUJ1+/Nestin + or GFAP+/Nestin + fluorescence intensity. *N* = 6, **** indicates *p* < 0.0001.

As expected, SCI induced a substantial increase in activated microglia (Iba1+), M1 microglia (Iba1+ and CD80+), and M2 microglia (Iba1+ and CD163+) in the spinal cord. Silencing ELAVL1 significantly suppressed microglial activation and M1 polarization in the SCI rats, while further promoting M2 polarization. However, shUSP29 treatment reversed the effects of ELAVL1 silencing (Fig. 9A). Additionally, SCI resulted in a marked reduction in NSCs (Nestin+), a significant decrease in neuronal differentiation (TUJ1+), and a notable increase in astrocyte differentiation (GFAP+). Compared to the SCI + shNC group, the SCI + shELAVL1 + shNC group showed a significant increase in Nestin + NSCs in the spinal cord tissues, effectively improving the differentiation dysregulation of NSCs towards neurons and astrocytes. However, silencing USP29 reversed the effect of shELAVL1 (Fig. 9B). These results indicate that silencing ELAVL1 modulates the USP29-TAK1 signaling pathway to induce M2 microglial polarization, which promotes the neuronal differentiation of NSCs, thereby alleviating neural injury and enhancing spinal cord repair in SCI rats.

## Discussion

SCI is a severe traumatic condition of the central nervous system that often leads to the loss of motor function and neurological impairment [28]. Despite ongoing efforts, no effective breakthrough has been achieved in the treatment of SCI, underscoring the urgent need to explore novel therapeutic strategies for its clinical management.

Recent studies have highlighted that the pivotal role of microglial polarization in the pathophysiology of SCI [11, 29, 30]. The polarization status of microglia influences the onset and progression of SCI, with an emphasis on the shift between pro-inflammatory M1 polarization and anti-inflammatory M2 polarization. As a result, modulating microglial polarization, specifically inhibiting the overactivation of M1 microglia and promoting M2 polarization, has been demonstrated to facilitate neural repair following SCI [31]. In this study, we successfully established a SCI rat model, characterized by motor dysfunction and spinal cord tissue damage [24]. We observed a significant increase in M1 microglia, consistent with previous reports on the inflammatory response post-SCI [32–35]. Through bioinformatics analysis, we identified the deubiquitinating enzyme USP29 as a key factor associated with SCI and microglial polarization. Notably, the expression of USP29 was significantly downregulated in the spinal cord tissues and microglia of SCI rats. Further experimental results revealed that overexpression of USP29 could inhibit LPS-induced M1 microglial polarization and promote, M2 polarization. This observation aligns with previous finding, which also demonstrated the crucial role of USP29 in regulating M2 microglial polarization [21].

TAK1 is a central regulatory factor in microglial polarization [23]. Our data indicate that USP29 not only inhibits the ubiquitination of TAK1 by binding to it but also further reduces the phosphorylation level of TAK1, thereby modulating its activation. These results corroborate prior research which reported similar regulatory effects of USP29 on TAK1 activity [36]. Furthermore, bioinformatic predictions led us to investigate the RNA-binding protein ELAVL1 (HuR) as an upstream regulator of USP29. It has been reported that ELAVL1 expression is upregulated after SCI [17], which is in line with our findings. We demonstrated that overexpression of ELAVL1 could suppress the stability of USP29 mRNA, thereby reducing its expression, while silencing ELAVL1 increased USP29 expression. Subsequent research revealed that ELAVL1 acts by binding to USP29 mRNA and destabilizing it. These findings are in agreement with previous work which confirmed that ELAVL1 promotes the mRNA degradation of downstream targets such as A20 [18]. Importantly, silencing ELAVL1 to regulate USP29 and inhibit TAK1 activation effectively reduced M1 microglial polarization while promoting the M2 microglial polarization in the spinal cord tissues of SCI rats. This observation offers a new molecular target for modulating microglial polarization after SCI.

Moreover, it has been well-documented that microglial polarization status is closely related to the neuronal-astrocytic fate differentiation of NSCs [15]. Our experimental results demonstrate that LPS-induced M1 microglial polarization not only promotes the differentiation of NSCs into astrocytes but also inhibits their differentiation into neurons. In contrast, inhibition of the ELAVL1-USP29-TAK1 pathway induced M2 polarization of microglia, which alleviated the differentiation dysregulation of NSCs. In the SCI rat model, inhibition of the ELAVL1-USP29-TAK1 pathway drove microglial polarization towards M2, enhancing neurogenesis of NSCs, helping motor function, and facilitating spinal cord tissue repair. These results are in line with other scholars who demonstrated that M2 polarization of microglia promotes the differentiation of NSCs into neurons, thereby aiding SCI repair [27].

This study thoroughly investigates the role of the ELAVL1-USP29-TAK1 axis in microglial polarization shift following SCI, revealing its impact on NSC fate differentiation. However, there are some limitations to the current study. Firstly, the study was conducted only in a rat model, and future research should validate these findings using other animal models and clinical samples to enhance the reliability and broader applicability of the results. Secondly, microglial polarization is a dynamic process. The lack of longitudinal assessment at multiple post-injury time points (e.g., 1, 2, and 4 weeks) limits our ability to fully elucidate the stage-specific contributions of this signaling axis. Future observations at different time points would provide a more comprehensive understanding of the role of this axis in microglial polarization and its subsequent effect on NSC differentiation following SCI. Furthermore, while our study elucidates the role of TAK1 in activating the NF-κB signaling pathway, other potential downstream signaling cascades, particularly the MAPK and JNK pathways, remain underexplored. Given MAPK and JNK pathways are critically involved in neuroinflammation and neuronal apoptosis following SCI [37], a more comprehensive investigation of these additional signaling branches is necessary to fully elucidate the multifaceted role of TAK1 in SCI pathogenesis.

In conclusion, this study reveals that ELAVL1 promotes USP29 mRNA degradation by binding to it, thereby increasing the ubiquitination and activation of TAK1. This drives microglial polarization towards the M1 phenotype, which not only supports NSC differentiation into astrocytes but also hinders their differentiation into neurons, ultimately exacerbating SCI pathology. Conversely, silencing ELAVL1 to regulate USP29 and inhibit TAK1 activation fosters M2 polarization of microglia, which supports neurogenesis and spinal cord repair. These findings provide new potential molecular targets for SCI treatment and offer valuable theoretical support for the development of novel therapeutic strategies for SCI.

## Materials and methods

### Bioinformatics analysis

The transcriptome sequencing dataset GSE245470 related to SCI was downloaded from the Gene Expression Omnibus (GEO) database (https://www.ncbi.nlm.nih.gov/gds/), which includes spinal cord tissue samples from 3 control mice (Sham-operated) and 3 SCI mice (SCI-modeled). Differentially expressed genes (DEGs) were identified using the “limma” R package with thresholds of *P*.adj < 0.05 and |log_2_(fold change)| > 1. The results of differential analysis were visualized using the “ggplot2” and “pheatmap” R packages. A Venn diagram was generated using the “venn” R package, and the intersecting genes were subjected to LASSO regression and random forest algorithms to identify potential feature genes using the “glmnet” and “randomForest” R packages, respectively. Finally, a Venn analysis was performed on the outputs from these two machine learning algorithms to identify core feature genes.

### Ethics statement

Female specific pathogen free (SPF)-grade Sprague-Dawley rats (101, Vital River, Beijing, China) aged 8–10 weeks and weighing 220–240 g were used in the study. All rats were housed in SPF-grade standard conditions, with a temperature maintained at 24–26°C, humidity at 45–55%, and a 12-h light/12-h dark cycle. The animals had free access to food and water and underwent a 7-day acclimation period to the start of the experiment. All animal procedures complied with relevant national regulations and institutional guidelines for the care and use of laboratory animals. The study was approved by the Ethics Committee of Shanghai Jing’an District Zhabei Central Hospital.

### SCI modeling and grouping

A total of 72 rats were randomly divided into 6 groups using a random number table method, with 12 rats in each group: the Sham group (with sham operation), the SCI group (with SCI modeling), the Sham + shNC group [sham-operated rats injected with empty lentiviral vector short hairpin RNA negative control (shNC)], the SCI + shNC group (SCI-modeled rats injected with shNC), the SCI + shELAVL1 + shNC group [SCI-modeled rats injected with ELAVL1 knockdown lentiviral vector shRNA against ELAVL1 (shELAVL1) and shNC], and the SCI + shELAVL1 + shUSP29 group [SCI-modeled rats injected with shELAVL1 and USP29 knockdown lentiviral vector shRNA against USP29 (shUSP29)]. All lentiviral vectors were purchased from HANBIO (Shanghai, China).

The SCI rat model was established by performing a complete spinal cord transection at the 10th thoracic vertebra (T10), as previously described [13]. In brief, the rats were anesthetized via inhalation of isoflurane, and the back fur was shaved and disinfected. A skin incision was made between the T9 and T11 vertebrae, exposing the T10 vertebra. Next, the T10 lamina was removed, and the spinal cord at the T10 was completely transected. Rats in the Sham and the Sham + shNC groups only underwent T10 laminectomy without spinal cord transection. For the Sham + shNC, the SCI + shNC, the SCI + shELAVL1 + shNC, and the SCI + shELAVL1 + shUSP29 groups, different lentiviral vectors (5 μL, 2 × 10^8^ TU/mL) were mixed with 5 μL of Matrigel and precisely injected into the injury site of the SCI rats to ensure effective delivery into the spinal cord tissue. All procedures were conducted in a sterile environment, and antibiotics were locally applied to the surgical area to prevent infection. Postoperative care includes artificial urination twice daily until the rats regained spontaneous urination.

### Behavioral assessments

The recovery of motor function in SCI rats was evaluated using the Basso-Beattie-Bresnahan (BBB) motor scale and the inclined plane test. Behavioral tests were conducted on days 1, 3, 7, 14, 21, and 28 post-surgery. The BBB motor scale was used to assess the hind limb motor ability of rats in an open field, with scores ranging from 0 to 21, where 0 indicates complete paralysis and 21 represents normal motor function. A higher score indicates better motor function recovery in the rats. The inclined plane test recorded the maximum angle at which the rats could maintain their position for 5 s without falling at different time points. A larger angle indicates facilitated recovery of motor function. All behavioral assessments were independently conducted by two evaluators blinded to group allocation, and the average of their results was used to ensure objectivity and consistency in the evaluation process.

### Hematoxylin-eosin (HE) staining

On day 28 post-SCI (28 dpi) when the behavioral assessments were completed, rats were anesthetized with isoflurane and then underwent cardiac perfusion with 0.9% NaCl (ST341, Beyotime, Shanghai, China) followed by 4% paraformaldehyde (P0099, Beyotime). After perfusion, the spinal cord was carefully exposed by a midline skin incision, and a 2–3 cm segment centered on the injury site was collected. The spinal cord was then cut into 5 mm-long segments and fixed overnight in 4% paraformaldehyde. After fixation, the spinal cord tissues were dehydrated through a graded alcohol series, cleared in xylene, and embedded in paraffin. Paraffin-embedded spinal cord tissues were sectioned into 5 μm thick slices using a Leica RM 2255 microtome (Wetzlar, Germany). The sections were stained using a HE staining kit (G1120, Solarbio, Beijing, China) following the standard procedure: the sections were deparaffinized with xylene, rehydrated with graded ethanol, and washed with distilled water. The sections were then stained with hematoxylin for 10 min, differentiated with differentiation solution for 30 s, and stained with eosin for 2 min. After washing, the sections were dehydrated, cleared, mounted, and observed under a microscope (BX53, Olympus, Tokyo, Japan), and images were captured for further analysis.

### Nissl staining

After deparaffinization and rehydration of the paraffin sections, Nissl staining was performed using a Nissl staining kit (S0197, BIOSS, Beijing, China). In brief, the sections were stained with methyl violet solution for 15 min, washed, and then differentiated with Nissl differentiation solution for 6 s. The sections were then dehydrated, cleared, and mounted, and were subsequently observed and photographed under a microscope (BX53, Olympus). Finally, The number of surviving neurons was quantified using ImageJ software.

### Immunohistochemistry (IHC)

On day 7 post-SCI (7 dpi), spinal cord tissues from rats were collected as described previously and prepared into paraffin sections. After deparaffinization and rehydration, antigen retrieval was performed by boiling the sections in citrate buffer (P0081, Beyotime) for 20 min. Endogenous peroxidase activity was blocked with 3% H_2_O_2_ solution (MM0750-500ML, Maokang Biotechnology, Shanghai, China) for 15 min. Sections were then blocked with 5% bovine serum albumin (BSA) (E-IR-R107, Elabscience, Wuhan, China) for 30 min to block nonspecific binding sites and incubated overnight at 4 °C with USP29 primary antibody (1:200, 27522-1-AP, Proteintech, Rosemont, IL, USA). Subsequently, the sections were treated with horseradish peroxide (HRP)-conjugated goat anti-rabbit IgG (1:2000; ab6702, Abcam, Cambridge, UK) at room temperature for 1 h The staining reaction was developed using a 3,3’-Diaminobenzidine HRP Color Development Kit (P0203, Beyotime). Finally, the sections were observed and photographed under a microscope (BX53, Olympus), with 3 independent fields analyzed per section. The percentage of USP29-positive cells was quantified using ImageJ software.

### Cell culture

Human microglial cells (HMC3, SNL-320, SUNNCELL, Wuhan, China) and human embryonic kidney cells (HEK293T, SCSP-502, Chinese Academy of Sciences Stem Cell Bank, Shanghai, China) were cultured in complete Dulbecco’s modified eagle’s medium (D6429, Sigma-Aldrich, St.Louis, MO, USA) containing 10% fetal bovine serum (FBS; A5256701, Gibco, Waltham, MA, USA) and 1% penicillin-streptomycin (PB180120, Procell). Human neural stem cells (HNSCs, SNP-H235, SUNNCELL) were seeded in cell culture dishes coated with poly-L-lysine (P6282, Sigma-Aldrich) and cultured in HNSCs complete medium (SNPM-H235, SUNNCELL). All cell cultures were incubated at 37°C in a humidified atmosphere containing 5% CO₂. Cells were passaged upon reaching 80-90% confluence. All cell lines were authenticated by Short Tandem Repeat profiling prior to the experiments. All cells were also tested for mycoplasma contamination, and the results were negative, ensuring the reliability of the experimental data.

### Cell transfection

HMC3 cells were infected differently with USP29 knockdown lentivirus (shUSP29-1, shUSP29-2, and shUSP29-3), ELAVL1 knockdown lentivirus (shELAVL1-1, shELAVL1-2, and shELAVL1-3), USP29 overexpression lentivirus (oe-USP29), ELAVL1 overexpression lentivirus (oe-ELAVL1), TAK1 overexpression lentivirus (oe-TAK1), and negative control lentiviruses (oe-NC and shNC) for 72 h at a multiplicity of infection (MOI) of 10. After infection, reverse transcription quantitative polymerase chain reaction (RT-qPCR) was carried out to verify the knockdown or overexpression efficiency. Lentiviral constructs with the highest efficiency in gene modulation (shUSP29 and shELAVL1) were selected for further experiments.

To explore the regulatory mechanism of USP29 on TAK1, the coding sequences of human USP29 and TAK1 were cloned into several expression vectors including pcDNA5, pcDNA5-Flag, pcDNA5-Myc, and GST-Myc, respectively, to generate overexpression plasmids (oe-USP29, Flag-USP29, Flag-TAK1, Myc-TAK1, GST-Myc-USP29, and GST-Myc-TAK1). These overexpression plasmids, along with GST-Myc control plasmids or HA-tagged ubiquitin plasmids (HA-Ub), were transfected into HEK293T cells using Lipofectamine™ 3000 transfection reagent (L3000015, ThermoFisher, Waltham, MA, USA). After 48 h of transfection, cells were harvested for subsequent analysis. All plasmids and lentiviruses were provided by HANBIO (Shanghai, China).

### HMC3 cell treatment and grouping

To simulate the inflammatory environment following SCI, infected or non-infected HMC3 cells were treated with 1 μg/mL lipopolysaccharide (LPS; ST1470, Beyotime) for 24 h to induce M1 microglial polarization, with an equal volume of phosphate buffer saline (PBS; C0221A, Beyotime) as a control. Based on different drug or lentiviral treatment conditions, the cells were divided into 14 experimental groups: the PBS group, the PBS + oe-NC group, the PBS + oe-USP29 group, the LPS group, the LPS + oe-NC group, the LPS + oe-USP29 group, the LPS + oe-ELAVL1 group, the LPS + shNC group, the LPS + shELAVL1 group, the LPS + shUSP29 group, the LPS + oe-USP29 + oe-NC group, the LPS + oe-USP29 + oe-TAK1 group, the LPS + shELAVL1 + shNC group, and the LPS + shELAVL1 + shUSP29 group.

### Cell counting kit-8 (CCK-8) assay

Infected or uninfected HMC3 cells were seeded into 96-well plates. After 24 h of incubation, cell viability was assessed using the CCK-8 assay kit (40203ES60, YEASEN, Shanghai, China). Briefly, cells were incubated with complete medium containing 10% CCK-8 solution for 2 h, and the absorbance was measured at 450 nm.

### Flow cytometry

HMC3 cells were digested with 0.05% trypsin (PB180222, Procell), centrifuged, and washed. The cell pellet was resuspended in fluorescence-activated cell sorting buffer (BL1136A, Biosharp, Anhui, China) to form a single-cell suspension. Cells were incubated with fluorescein isothiocyanate (FITC)-labeled antibodies against CD80 (11-0809-42, ThermoFisher) or CD163 (MA5-17719, ThermoFisher) at room temperature in the dark for 30 min. Finally, the percentage of M1 microglia (CD80^+^) and M2 microglia (CD163^+^) was quantified using an Accuri C6 Plus flow cytometer (BD Biosciences, Franklin Lakes, NJ, USA).

### Enzyme-linked immunosorbent assay (ELISA)

After different treatments, the culture supernatants of HMC3 cells were collected and centrifuged at 300 × *g* for 10 min to remove cell debris. The concentrations of pro-inflammatory cytokines associated with M1 polarization [tumor necrosis factor-alpha (TNF-α) and interleukin-1 beta (IL-1β)] as well as anti-inflammatory cytokines related to M2 polarization [transforming growth factor-beta (TGF-β) and IL-10] in the supernatant were measured using TNF-α (EK182, Multi Sciences, Hangzhou, China), IL-1β (EK101B, Multi Sciences), TGF-β (PT880, Beyotime), and IL-10 (EHIL10, ThermoFisher) kits.

### RT-qPCR

Total RNA was extracted from HMC3 cells using Trizol reagent (B511311-0100, Sangon Biotech, Shanghai, China). Subsequently, reverse transcription was performed using the HiScript III RT SuperMix for qPCR (+gDNA wiper) kit (R323-01, Vazyme, Nanjing, China) to synthesize cDNA. Real-time qPCR was performed using ChamQ Universal SYBR qPCR Master Mix (Q711-02, Vazyme) on the QuantStudio 3 Real-Time PCR Systems (ThermoFisher), with β-actin used as an internal reference, and the relative expression of genes was calculated using the 2^−∆∆Ct^ method. Specific primer sequences for each gene were synthesized by Sangon Biotech and listed in Table S1.

### Western blot analysis

Total proteins were extracted from cells and spinal cord tissues (7 dpi) using radioimmunoprecipitation assay lysis buffer (20101ES60, YEASEN) supplemented with phenylmethylsulfonyl fluoride protease inhibitor (20104ES03, YEASEN). After thorough lysis, samples were centrifuged at 14,000 × *g* for 3 min to collect the supernatant. Protein concentration was quantified using the BCA protein assay kit (20201ES76, YEASEN). Equal amounts of protein were separated by SDS-PAGE (8–12% gel) and transferred to a polyvinylidene fluoride (PVDF) membrane (FFP39, Beyotime). The membrane was blocked with 5% skim milk (P0216, Beyotime) at room temperature for 1 h, followed by incubation overnight at 4°C with primary antibodies against USP29 (1:300, 27522-1-AP, Proteintech, Woburn, MA, USA), iNOS (1:1000, 18985-1-AP, Proteintech), CD206 (1:1000, 18704-1-AP, Proteintech), ELAVL1 (1:4000, 11910-1-AP, Proteintech), phosphorylated TAK1 (p-TAK1; 1:1000, AP0071, ABclonal), TAK1 (1:1000, A12022, ABclonal), Flag (1:20,000, 20543-1-AP, Proteintech), Myc (1:1000, 16286-1-AP, Proteintech), HA (1:1000, #3724, Cell Signaling Technology, Danvers, MA, USA), and β-actin (1:4000, 20536-1-AP, Proteintech). After washing, the membrane was incubated with HRP-conjugated goat anti-rabbit IgG (1:2000, ab6702, Abcam) at room temperature for 1 h. The membrane was then treated with enhanced chemiluminescence working solution (E411-04, Vazyme) at room temperature for 2 min and visualized using the ChemiDoc MP imaging system (Bio-RAD, Hercules, CA, USA). β-actin was used as internal reference, and protein band intensities were quantified and normalized using ImageJ software.

### Immunofluorescence (IF)

For tissue IF staining, paraffin-embedded spinal cord tissue sections (7 dpi) were deparaffinized and rehydrated. Antigen retrieval was performed by boiling the sections for 15 min in 1× ethylenediaminetetraacetic acid antigen retrieval buffer (pH 8.0; P0085, Beyotime). For cell IF, cells treated differently were fixed with 4% paraformaldehyde for 30 min, followed by permeabilization with 0.1% Triton X-100 solution (ST797, Beyotime) for 30 min. Both tissue sections and cells were blocked with 5% BSA (E-IR-R107, Elabscience) for 30 min, followed by overnight incubation at 4°C with primary antibodies: Iba1 (1:200; MA5-27726, ThermoFisher), CD80 (1:100; PA5-79001, ThermoFisher), rabbit anti-CD163 (1:100, 16646-1-AP, Proteintech), mouse anti-CD163 (1:100, ab156769, Abcam), USP29 (1:300; 27522-1-AP, Proteintech), ELAVL1 (1:100; 11910-1-AP, Proteintech), p-TAK1 (1:200; AF3019, Affinity), Nestin (1:500; MA5-47428, ThermoFisher), mouse anti-TUJ1 (1:200; 66375-1-Ig, Proteintech), rabbit anti-TUJ1 (1:200; PA5-85639, ThermoFisher), and GFAP (1:100; 16825-1-AP, Proteintech). Subsequently, the sections and cells were incubated with secondary antibodies: Cy3-conjugated goat anti-mouse IgG (1:200; A10521, ThermoFisher) and FITC-conjugated goat anti-rabbit IgG (1:1000; F-2765, ThermoFisher) at 37 °C for 1 h. Afterward, the cell nuclei were stained with DAPI (C1005, Beyotime) and mounted with anti-fade mounting medium (P0126, Beyotime). Fluorescence images were captured using an LSM 880 confocal microscope (Zeiss, Germany). For each tissue section and each cell culture well, three independent fields of view were selected for observation, and the fluorescence intensity was quantified using ImageJ software.

### Co-immunoprecipitation (Co-IP)

HEK293T cells were transfected with Flag-USP29 and Myc-TAK1 plasmids for 48 h. Co-IP was performed using the Protein A/G IP/Co-IP kit (YJ201, Epizyme Biotech, Cambridge, MA, USA) according to the manufacturer’s instructions. The detailed procedure is as follows: Cells were lysed on ice for 20 min using lysis buffer containing protease inhibitors (GRF101, Epizyme Biotech). After lysis, the samples were centrifuged at 16,000 × *g* and 4 °C for 10 min, and the supernatant was collected for further use. The supernatant was incubated with Flag (20543-1-AP, Proteintech) or Myc (16286-1-AP, Proteintech) antibodies at 4 °C for 4 h to form the antigen-antibody complex. Then, pre-treated Protein A/G magnetic beads were added and incubated overnight at 4 °C to capture the antigen-antibody-bead complex. After washing the magnetic beads with washing buffer, the complex was resuspended in 80 µl 1× SDS-PAGE sample buffer and heated at 100 °C for 10 min to elute the antigen. Finally, the eluted supernatant was collected for Western blot analysis.

### Glutathione S-transferase (GST) pull-down

After HEK293T cells were transfected with Flag-USP29, GST-Myc-TAK1, Flag-TAK1, GST-Myc-USP29, or GST-Myc plasmids for 48 h, GST pull-down detection was performed using a GST pull-down kit (JKR23011, GENE CREATE, Wuhan, China). Briefly, cell lysates were prepared by incubation on ice for 30 min in lysis buffer containing protease inhibitors, followed by sonication in an ice bath for 5 min using an ultrasonic cell crusher (E0380, Beyotime). After centrifugation at 12,000 rpm and 4 °C for 10 min, the supernatant was collected and incubated at room temperature with Glutathione MagBeads for 2 h to capture GST-tagged proteins (GST-Myc-TAK1, GST-Myc-USP29, or GST-Myc). The total protein and magnetic bead-GST fusion protein complex were incubated overnight at 4 °C, eluted with buffer, and heated in a boiling water bath for 10 min, followed by centrifugation at 12,000 rpm for 5 min to collect the supernatant. Finally, the samples were mixed with loading buffer, heated in a boiling water bath for 10 min, and subjected to Western blot analysis.

### Ubiquitination detection

HEK293T cells were transfected with Myc-TAK1, Flag-USP29, or HA-Ub plasmids for 48 h. Cells were lysed with lysis buffer containing protease inhibitors, and the lysate was incubated with Myc antibody (16286-1-AP, Proteintech) at 4 °C for 4 h. Then, Protein A/G magnetic beads were added, and the mixture was incubated overnight at 4 °C. After washing the magnetic beads, the protein complex was resuspended in SDS-PAGE sample buffer and heated at 100 °C for 10 min. Finally, the eluted protein supernatant was collected and subjected to Western blot analysis to assess the ubiquitination level of Myc-TAK1.

### Actinomycin D assay

To assess stability of USP29 mRNA, HMC3 cells were exposed to 4 μg/mL actinomycin D (HY-17559, MCE, Monmouth, NJ, USA) and collected at different time points (0, 1, 2, and 3 h). Subsequently, USP29 mRNA levels were then measured by RT-qPCR.

### RNA immunoprecipitation (RIP)-qPCR

RIP was performed using the BeyoRIP^TM^ RIP Assay Kit (P1801S, Beyotime). In brief, 1.5 × 10^6^ HMC3 cells were resuspended in 300 μL of lysis buffer and incubated on ice for 15 min. After centrifugation at 14,000 × *g*, 4 °C for 10 min, the supernatant was harvested and stored for further use. Protein A/G Agarose beads were conjugated with ELAVL1 antibody (11910-1-AP, Proteintech) or IgG control antibody (A7016, Beyotime), which was incubated with lysates overnight at 4 °C to capture the protein-RNA complexes. The agarose beads were washed with NT2 wash buffer, and 100 μL elution buffer was added, followed by incubation at 55 °C for 30 min. One portion of the eluted complex was used for Western blot analysis to assess the RIP efficiency, while the other portion was used for RNA purification. The purified RNA was then analyzed by RT-qPCR to measure USP29 mRNA enrichment.

### Co-culture of HMC3 with HNSCs

HNSCs were seeded into the basolateral chamber of a poly-L-lysine-coated Transwell co-culture chamber (FTW001, Beyotime), and differentially treated HMC3 cells were seeded into the apical chamber of the Transwell at a 1:1 ratio. After 7 days of co-culture, HNSCs were collected for subsequent analysis. The experiment was divided into 8 groups according to the different treatments of co-cultured HMC3 cells: the PBS group, the LPS group, the LPS + shNC group, the LPS + shELAVL1 + shNC group, the LPS + shELAVL1 + shUSP29 group, the LPS + oe-NC group, the LPS + oe-USP29 + oe-NC group, and the LPS + oe-USP29 + oe-TAK1 group.

### 5-Ethynyl-2’-deoxyuridine (EdU) staining

Following the BeyoClick^TM^ EdU-594 Cell Proliferation Detection Kit (C0078S, Beyotime) instructions, HNSCs were incubated with 10 µM EdU for 1 day after 7 days of co-culture. Then, the cells were fixed with 4% paraformaldehyde at room temperature for 15 min, permeabilized with 0.3% Triton X-100 at room temperature for 15 min, incubated with Click reaction solution (0.5 mL per well) in the dark at room temperature for 30 min for EdU detection. After washing 3 times, nuclei were stained with 1 mL Hoechst 33342 solution (1×) in the dark at room temperature for 10 min. Finally, images were captured using an LSM 880 confocal microscope (Zeiss, Germany). Three independent fields per well were observed, and ImageJ software was used to quantitatively analyze the percentage of EdU-positive cells.

### Statistical analysis

Statistical analysis was performed using GraphPad Prism 10.1.2 (San Diego, CA, USA). All measurement data are presented as the mean ± standard error of the mean (SEM). All experiments were performed at least three times. The normality of the data was assessed using the Shapiro–Wilk test, and homogeneity of variance was evaluated using Levene’s test. For data conforming to normal distribution and homogeneity of variance, comparisons between two groups were conducted using independent samples *t-*test. For comparisons among three or more groups, one-way analysis of variance (ANOVA) was used, followed by Tukey’s HSD post-hoc test. When data did not conform to normal distribution or homogeneity of variance, the Wilcoxon rank-sum test or Kruskal–Wallis test was employed. A *p* < 0.05 was considered statistically significant.

## Supplementary information

Table S1

Figure S1-S4

Original figures

## Data Availability

The transcriptome sequencing dataset GSE245470 related to spinal cord injury (SCI) was downloaded from the GEO database (https://www.ncbi.nlm.nih.gov/gds/).
