# Supplementary material for: ELAVL1-mediated USP29 mRNA degradation activates TAK1 driving M1 microglial polarization and neural stem cell differentiation dysregulation in spinal cord injury
Source: Cell Death Discov. 2025 Jul 9;11:317. doi: 10.1038/s41420-025-02604-8 (PMC12241534; doi:10.1038/s41420-025-02604-8)
Supplement: Supplementary file 1 — Table S1 [file 41420_2025_2604_MOESM1_ESM.docx]

**Table S1. Primer sequences of RT-qPCR**

| Gene | Species | Primer sequence |
| --- | --- | --- |
| ELAVL1 | Human | F 5’- TGTTCTCTCGGTTTGGGCGGAT -3’ |
|  |  | R 5’- TCTTCTGCCTCCGACCGTTTGT -3’ |
| USP29 | Human | F 5’- CCAGTGATTCCCTGGTTCTACC -3’ |
|  |  | R 5’- GCAGAGCCATTTTCCAGGTCTC -3’ |
| TUJ1 | Human | F 5’- TCAGCGTCTACTACAACGAGGC -3’ |
|  |  | R 5’- GCCTGAAGAGATGTCCAAAGGC -3’ |
| GFAP | Human | F 5’- CTGGAGAGGAAGATTGAGTCGC -3’ |
|  |  | R 5’- ACGTCAAGCTCCACATGGACCT -3’ |
| β-actin | Human | F 5’- GGCACCCAGCACAATGAAG -3’ |
|  |  | R 5’- CCGATCCACACGGAGTACTTG -3’ |

Note: ELAVL1, ELAV like RNA binding protein 1; USP29, ubiquitin specific peptidase 29; TUJ1 (TUBB3), tubulin beta 3 class Ⅲ; GFAP, glial fibrillary acidic protein; β-actin, actin beta; F, Forward; R, Reverse.
