# Supplementary material for: ELAVL1-mediated USP29 mRNA degradation activates TAK1 driving M1 microglial polarization and neural stem cell differentiation dysregulation in spinal cord injury
Source: Cell Death Discov. 2025 Jul 9;11:317. doi: 10.1038/s41420-025-02604-8 (PMC12241534; doi:10.1038/s41420-025-02604-8)
Supplement: Supplementary file 2 — Figure S1-S4 [file 41420_2025_2604_MOESM2_ESM.docx]

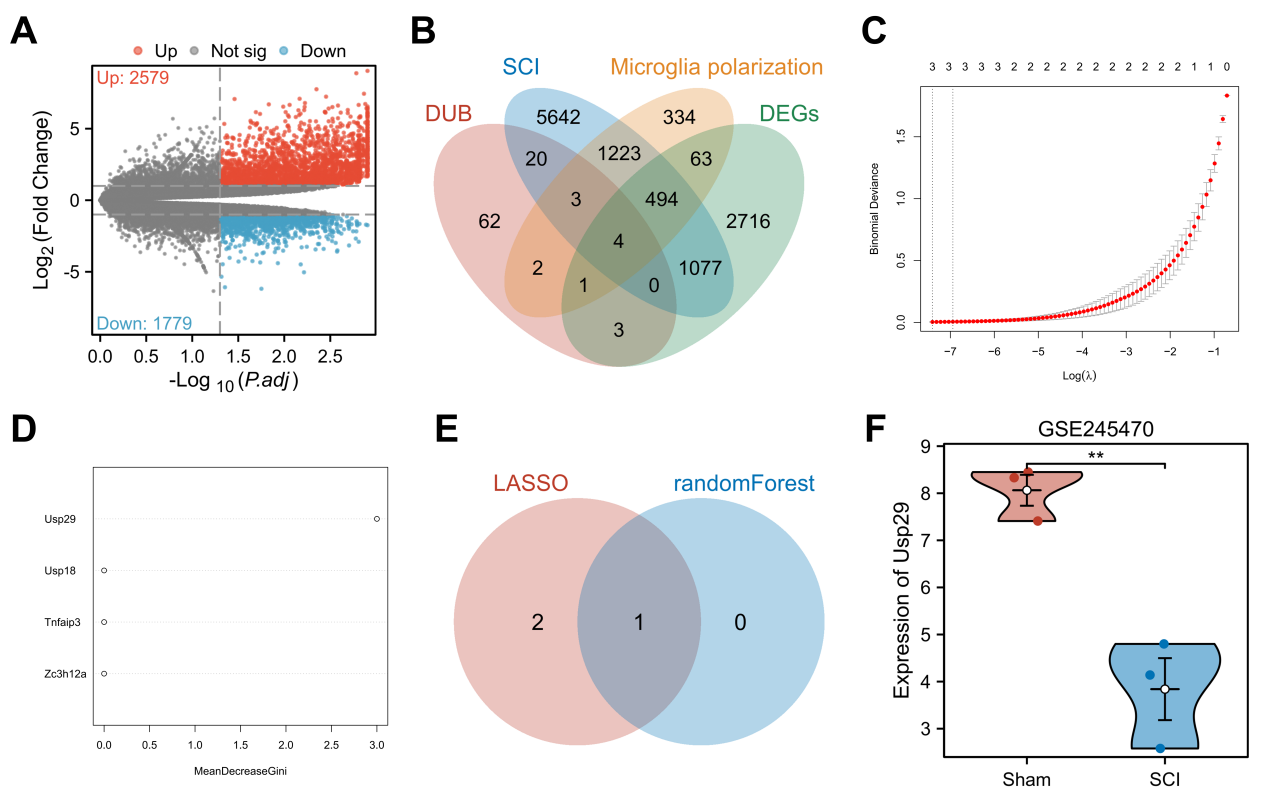


**Figure S1. Screening of the core candidate gene from the GSE245470 dataset.**

Notes: (A) Volcano plot of differential analysis for the GEO database dataset GSE245470. (B) Venn diagram of DUBs, DEGs, microglial polarization- and SCI-related genes. (C) LASSO regression results of the intersected genes. (D) Random forest algorithm results of the intersected genes. (E) Venn diagram of LASSO regression and random forest algorithm results. (F) Expression of USP29 in the GSE245470 dataset.


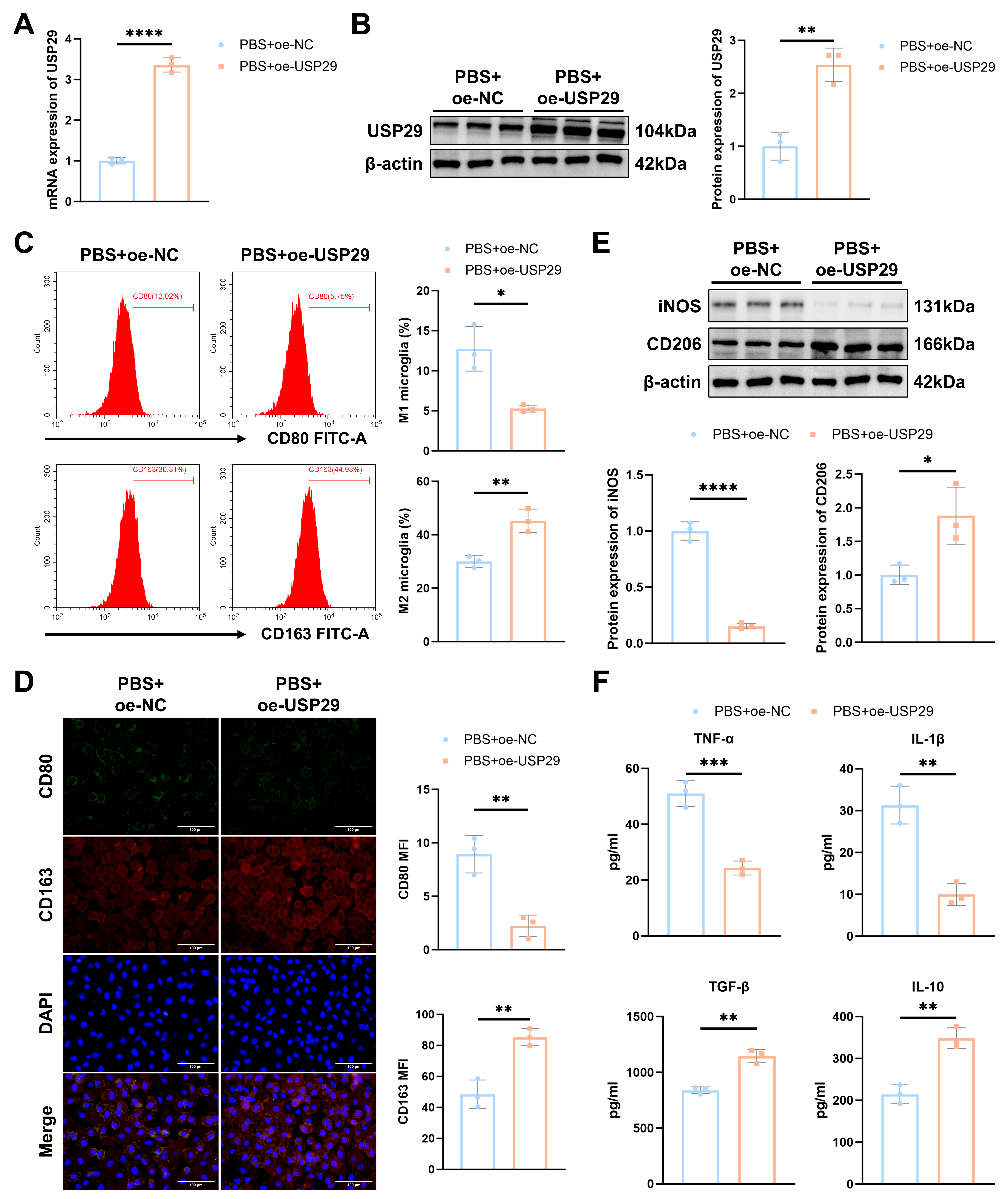


**Figure S2. Effect of USP29 on polarization of HMC3 cells.**

Note: (A) RT-qPCR detection of USP29 mRNA expression levels in HMC3 cells. (B) Western blot detection of USP29 protein expression in HMC3 cells. (C) Flow cytometry detection of the percentage of M1 and M2 microglia in each group of cells. (D) Representative IF images of CD80 and CD163 in cells from each group (scale bar = 100 μm), and quantification of average fluorescence intensity. (E) Western blot detection of iNOS and CD206 protein expression in cells. (F) ELISA detection of the levels of pro-inflammatory cytokines and anti-inflammatory cytokines in cell culture supernatants. * indicates *p* < 0.05, ** indicates *p* < 0.01, *** indicates *p* < 0.001, **** indicates *p* < 0.0001. All experiments were performed in triplicate.


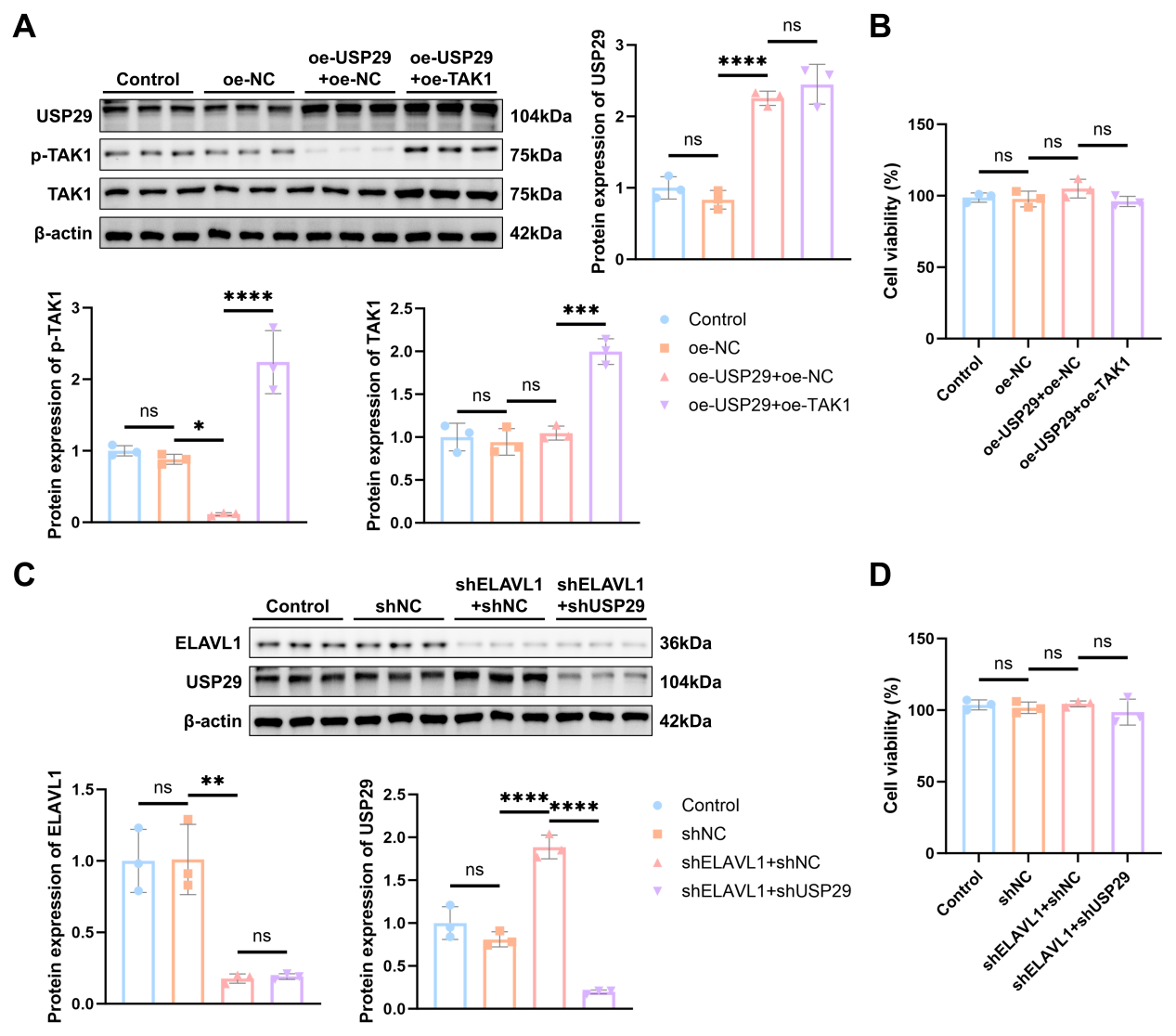


**Figure S3. The effect of lentiviral infection on expression of key factors and cell viability**

Note: (A) Western blot detection of USP29, p-TAK1, and TAK1 expression in HMC3 cells. (B) CCK8 assay for cell viability in each group. (C) Western blot detection of ELAVL1 and USP29 expression in HMC3 cells. (D) CCK8 assay for cell viability in each group. All cell experiments were performed in triplicate. ns indicates *p* > 0.05, * indicates *p* < 0.05, ** indicates *p* < 0.01, *** indicates *p* < 0.001, **** indicates *p* < 0.0001.


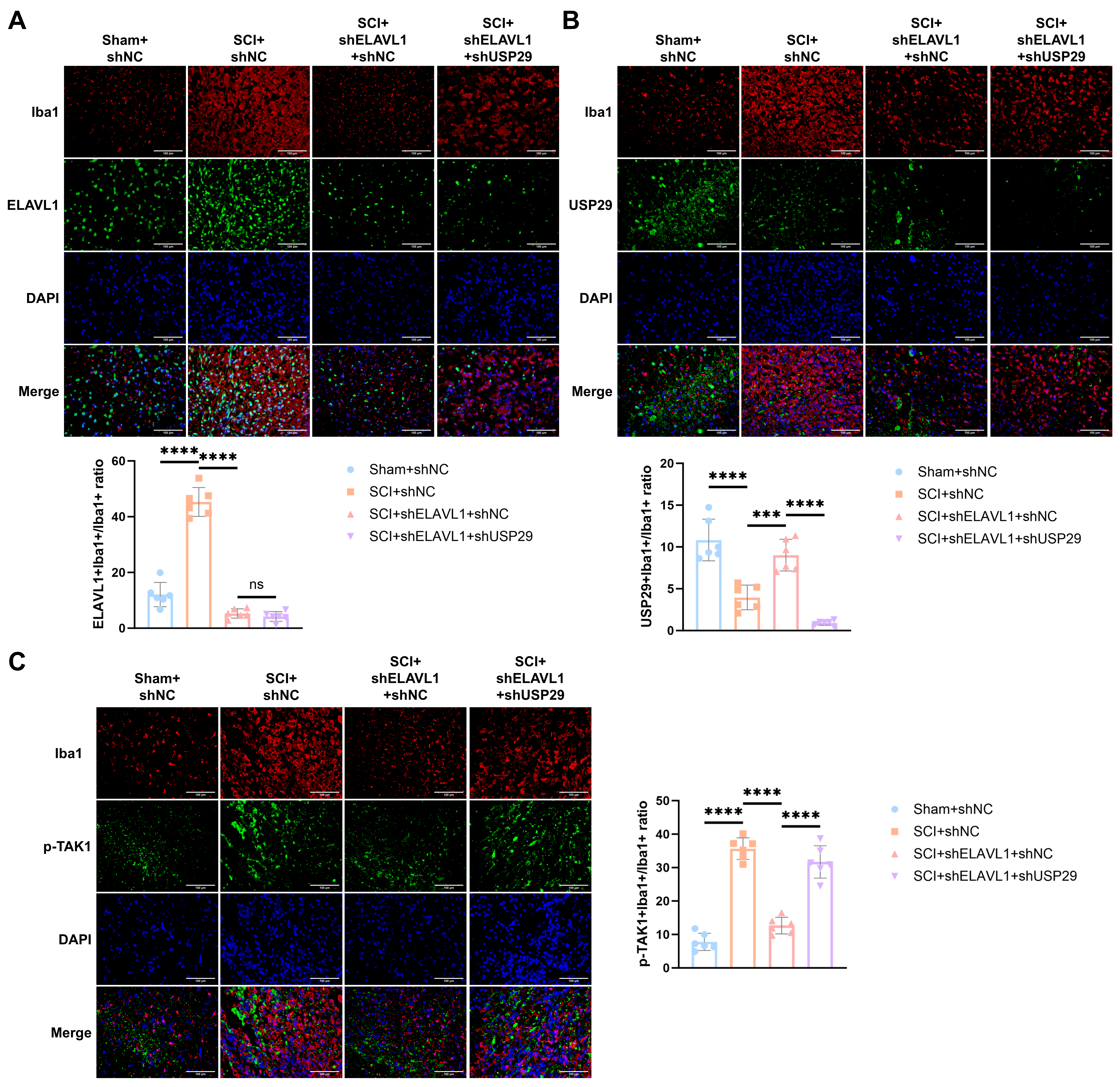


**Figure S4. Expression of ELAVL1, USP29, and p-TAK1 in microglia of rat spinal cord tissue**

Note: (A) Representative images of Iba1 and ELAVL1 IF staining on day 7 post-SCI (scale bar = 100 μm), and quantification of the ratio of ELAVL1⁺Iba1⁺ to Iba1⁺ average fluorescence intensity. (B) Representative images of Iba1 and USP29 IF staining on day 7 post-SCI (scale bar = 100 μm), and quantification of the ratio of USP29⁺Iba1⁺ to Iba1⁺ average fluorescence intensity. (C) Representative images of Iba1 and p-TAK1 IF staining on day 7 post-SCI (scale bar = 100 μm), and quantification of the ratio of p-TAK1⁺Iba1⁺ to Iba1⁺ average fluorescence intensity. N = 6, ns indicates *p* > 0.05, *** indicates *p* < 0.001, **** indicates *p* < 0.0001.
