# Supplementary material for: ELAVL1-mediated USP29 mRNA degradation activates TAK1 driving M1 microglial polarization and neural stem cell differentiation dysregulation in spinal cord injury
Source: Cell Death Discov. 2025 Jul 9;11:317. doi: 10.1038/s41420-025-02604-8 (PMC12241534; doi:10.1038/s41420-025-02604-8)

Marker: G2086, Servicebio

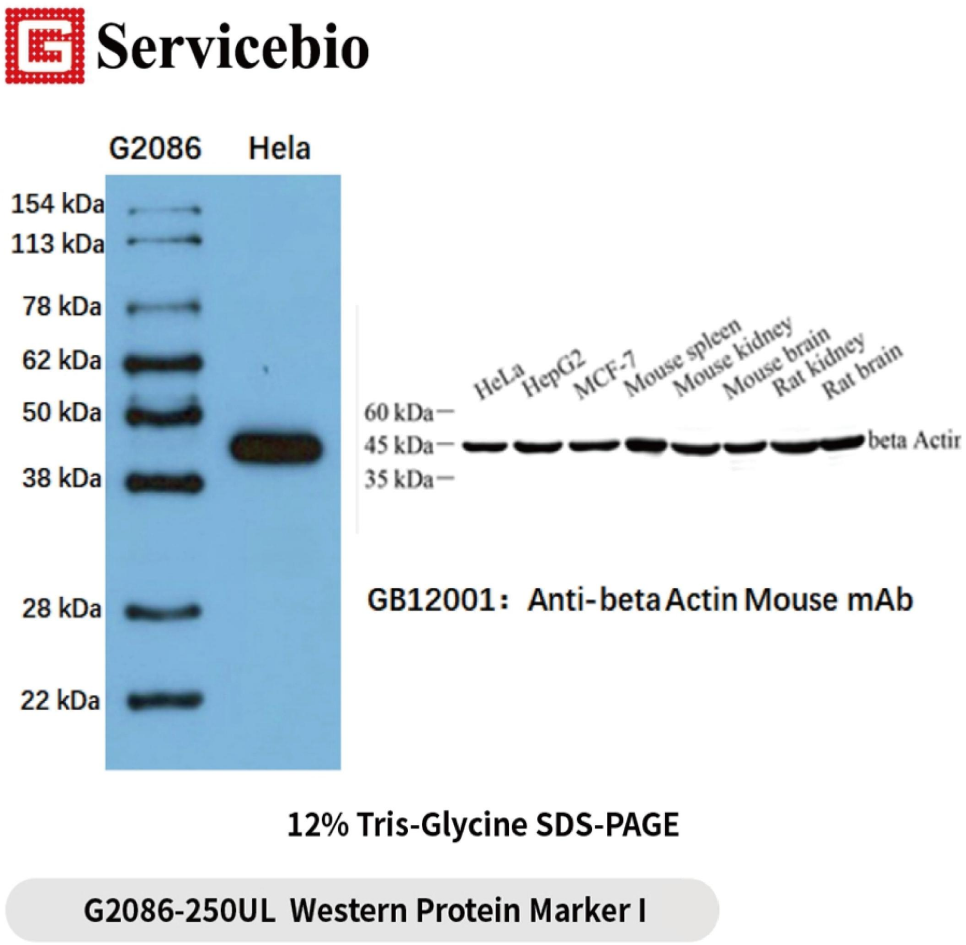

Figure 1G. Original western blot.

Figure 1G shows the whole blot after cutting membrane at molecular weight USP29 (104kDa) and β-actin (42kDa).

USP29
104kDa

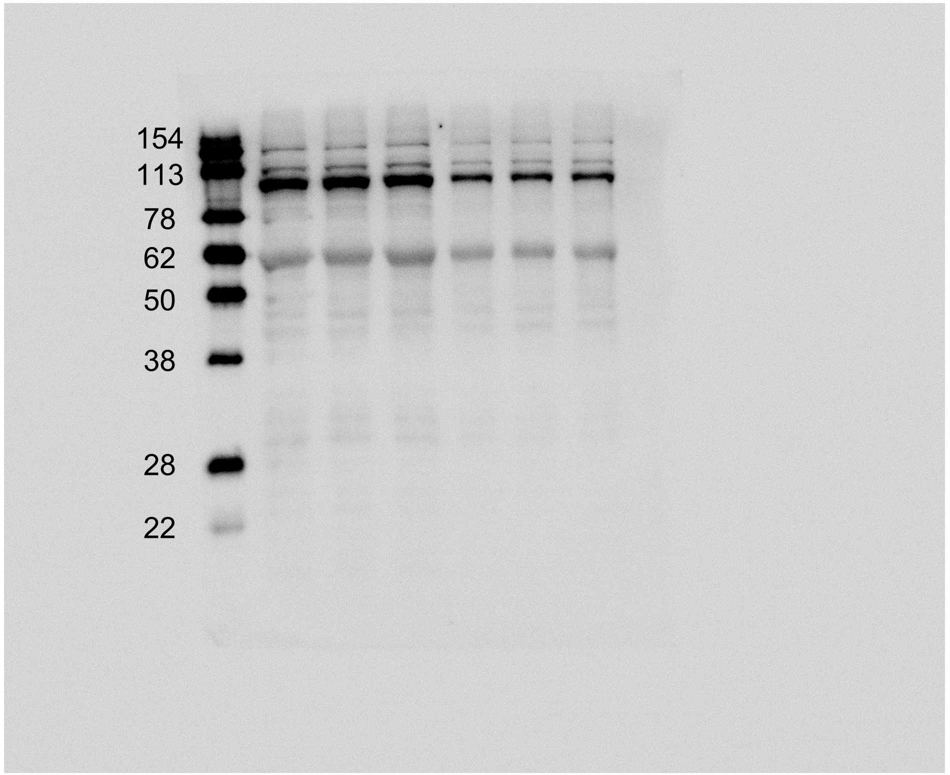

β-actin
42kDa

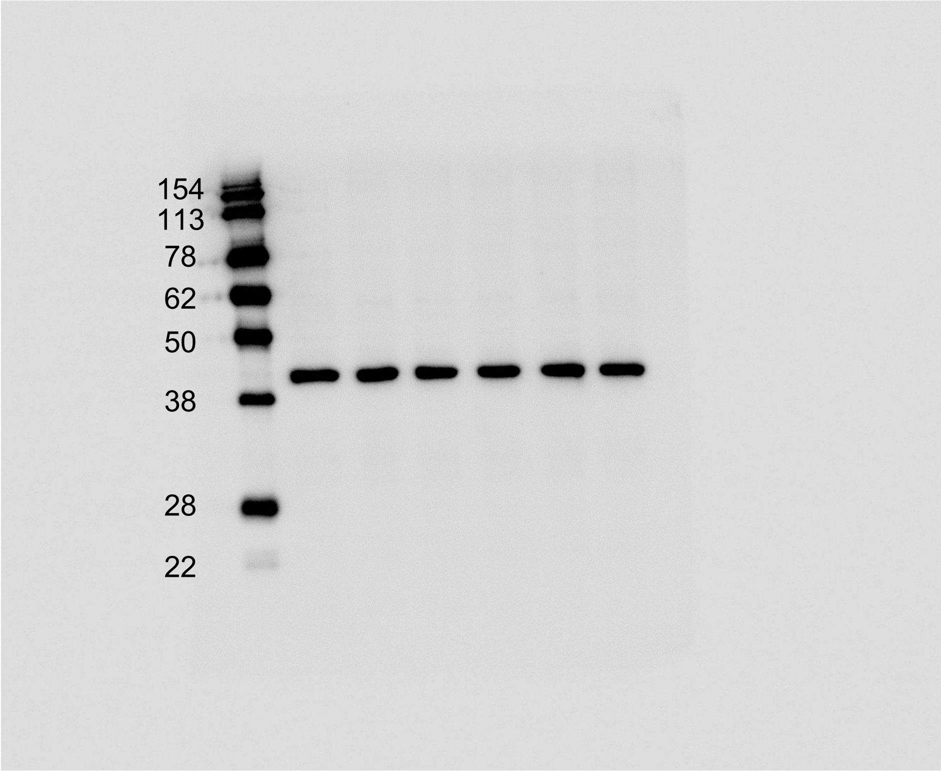

Figure 2C. Original western blot.

Figure 2C shows the whole blot after cutting membrane at molecular weight USP29 (104kDa) and β-actin (42kDa).

USP29
104kDa

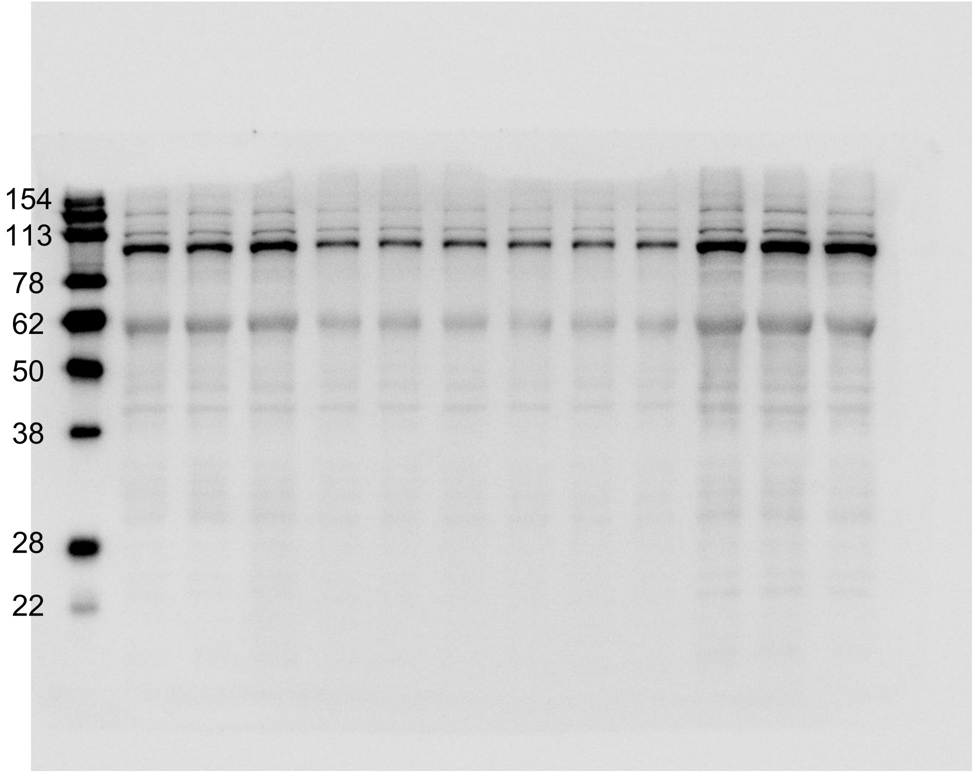

β-actin
42kDa

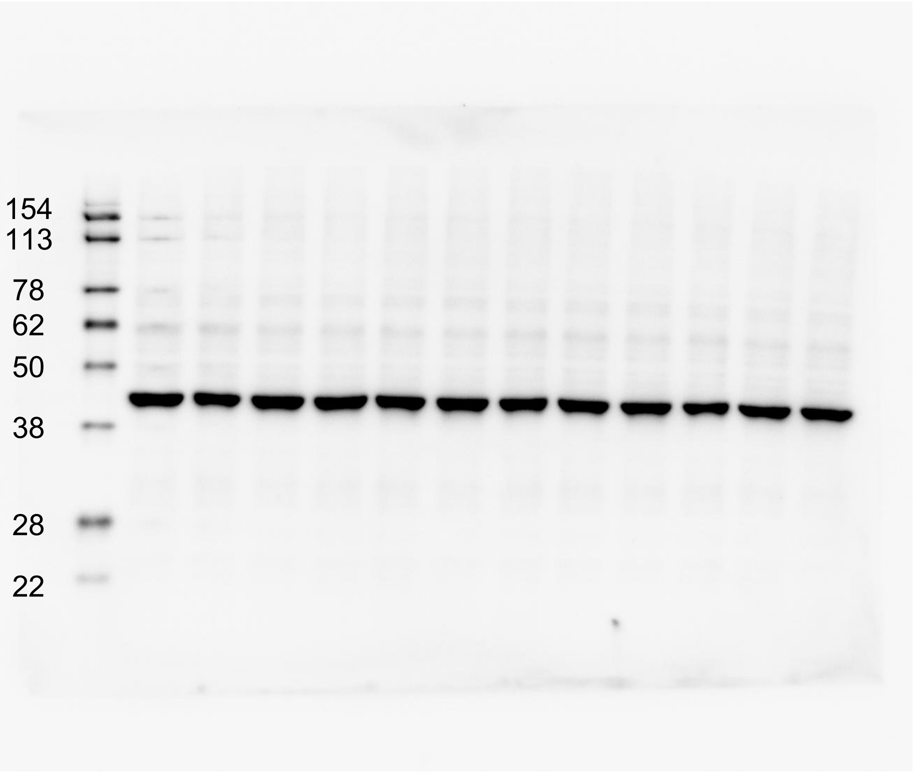

Figure 2F. Original western blot.

Figure 2F shows the whole blot after cutting membrane at molecular weight iNOS (131kDa), CD206 (166kDa) and β-actin (42kDa).

iNOS 131kDa

CD206 166kDa

β-actin 42kDa

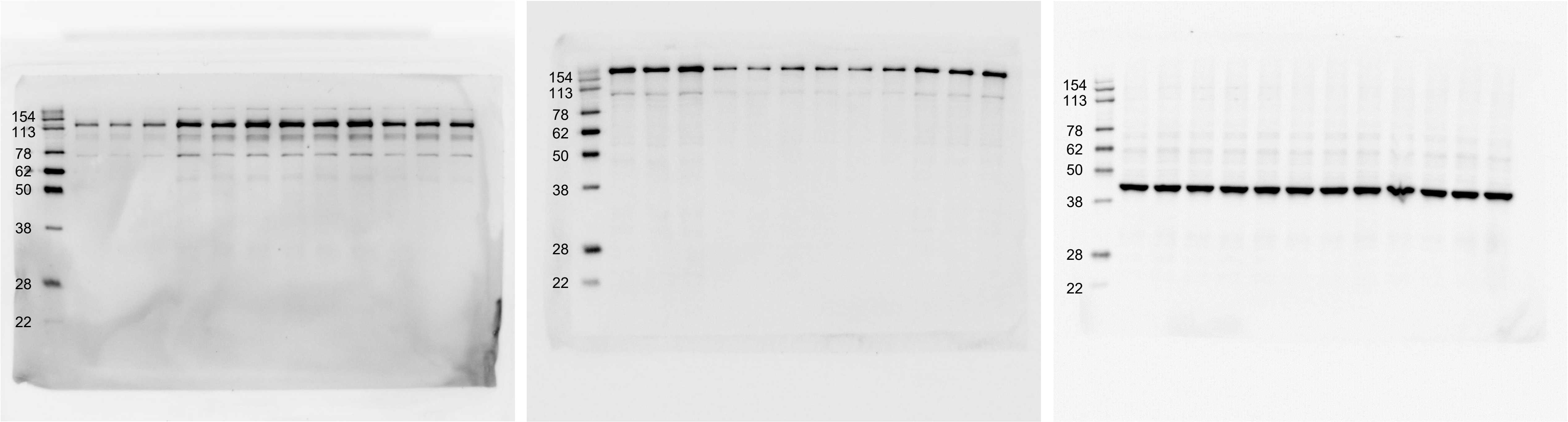

Figure 3C. Original western blot.

Figure 3C shows the whole blot after cutting membrane at molecular weight p-TAK1 (75kDa), TAK1 (75kDa) and β-actin (42kDa).

p-TAK1
75kDa

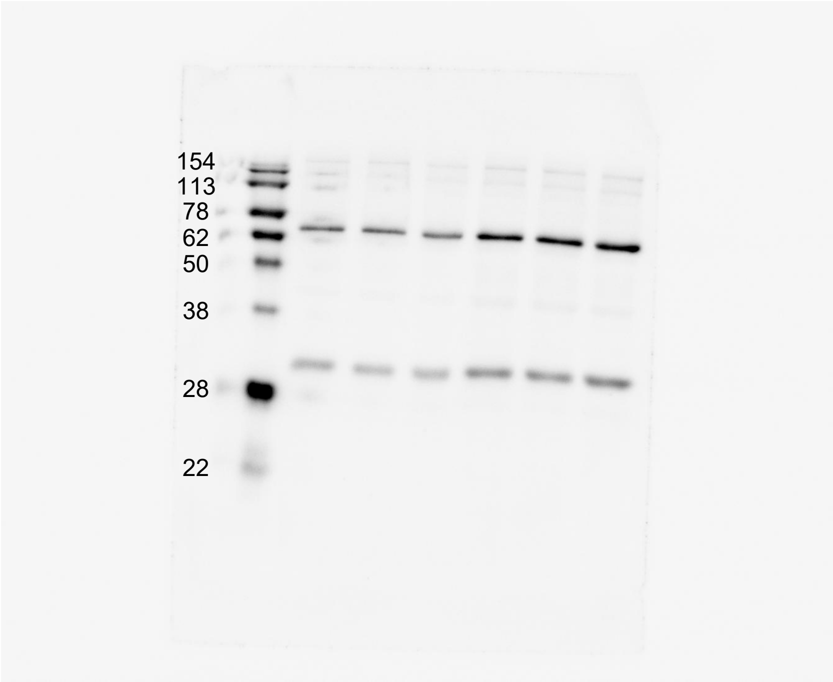

TAK1
75kDa

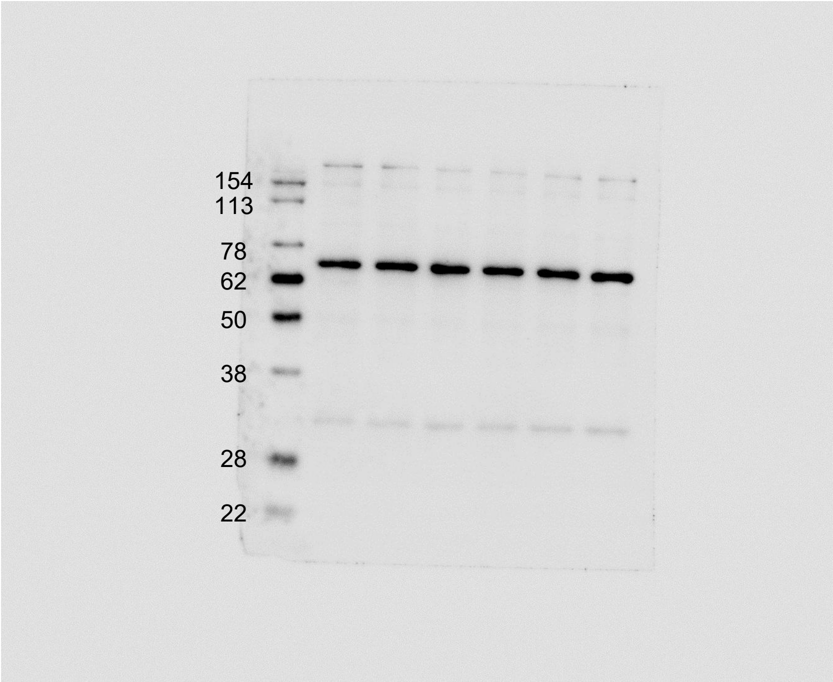

β-actin
42kDa

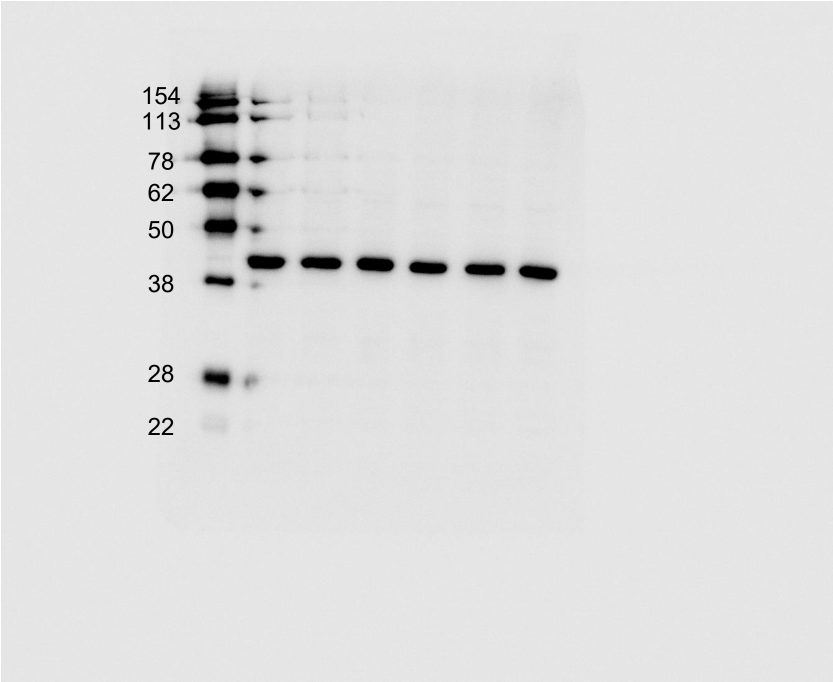

Figure 3D. Original western blot.

Figure 3D shows the whole blot after cutting membrane at molecular weight p-TAK1 (75kDa), TAK1 (75kDa) and β-actin (42kDa).

p-TAK1
75kDa

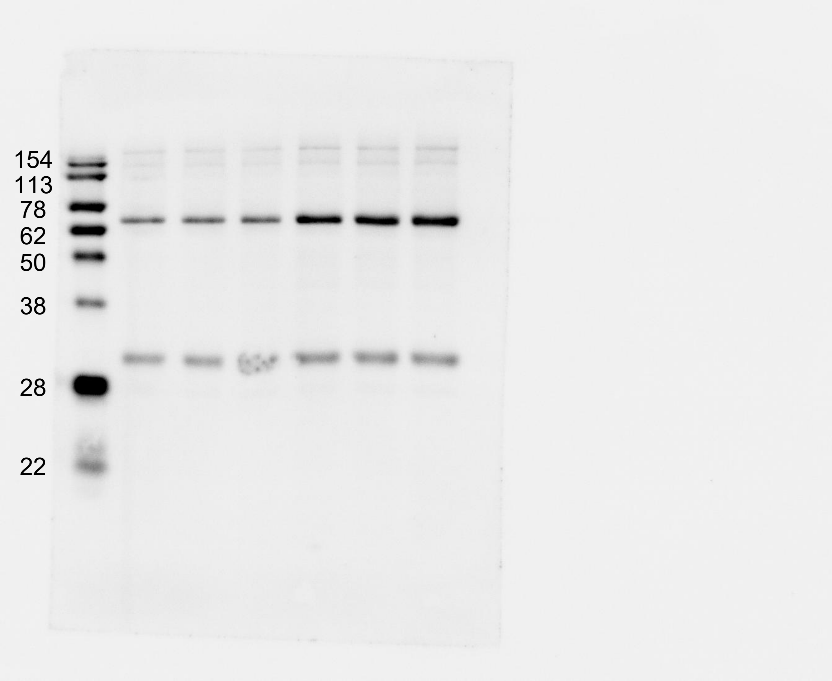

TAK1
75kDa

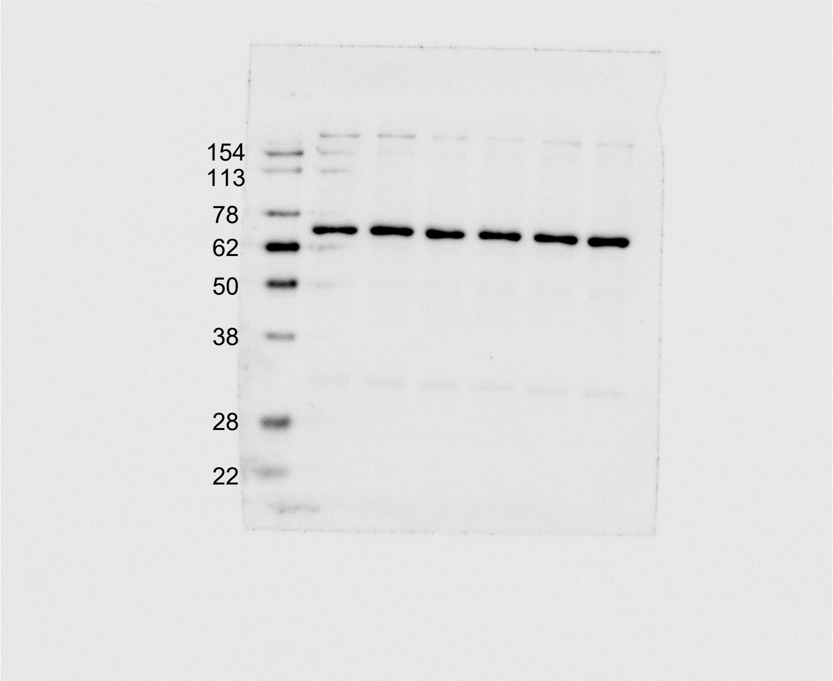

β-actin
42kDa

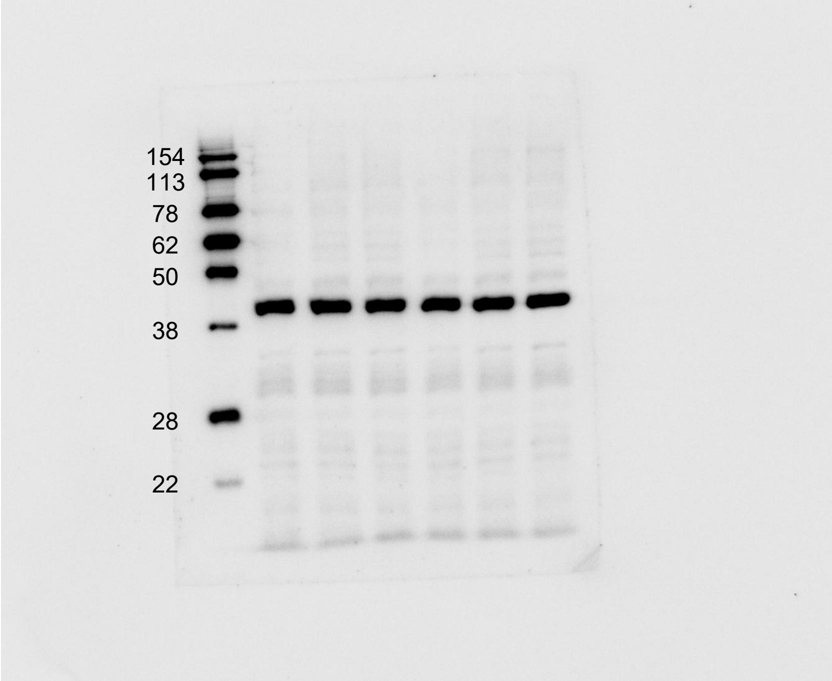

Figure 3F. Original western blot.

Figure 3F shows the whole blot after cutting membrane at molecular weight p-TAK1 (75kDa), TAK1 (75kDa) and β-actin (42kDa).

p-TAK1
75kDa

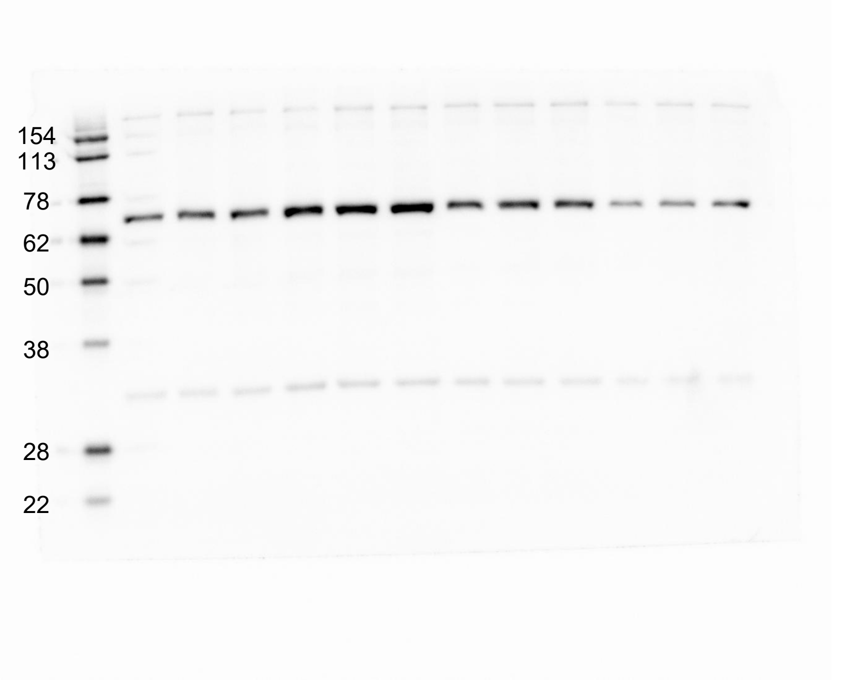

TAK1
75kDa

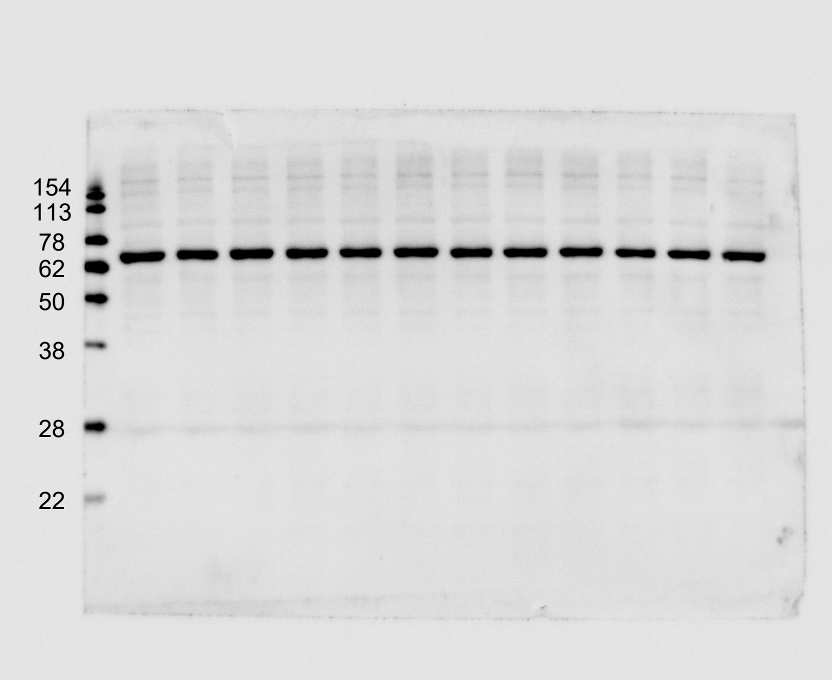

β-actin
42kDa

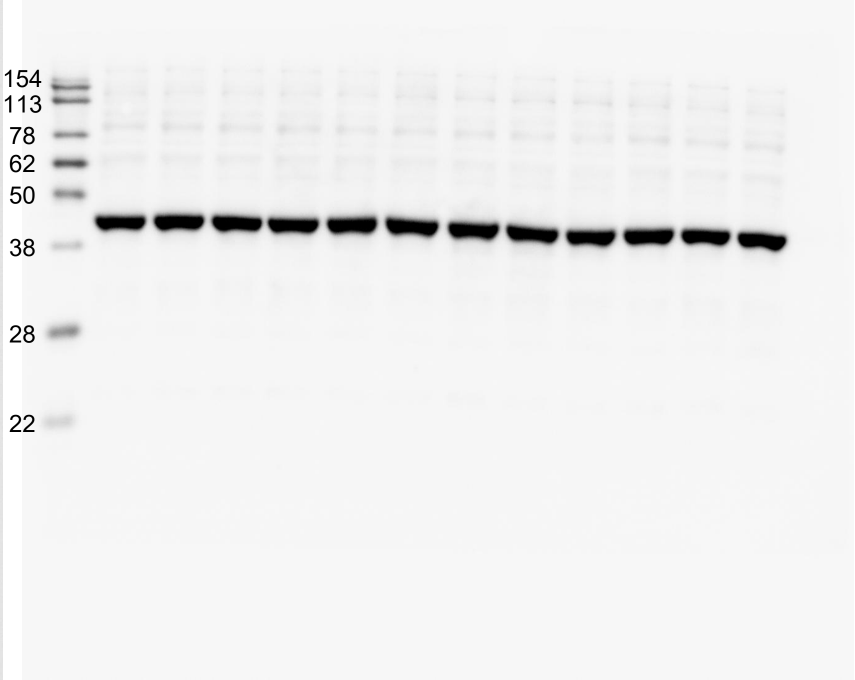

Figure 3G. Original western blot.

USP29
104kDa

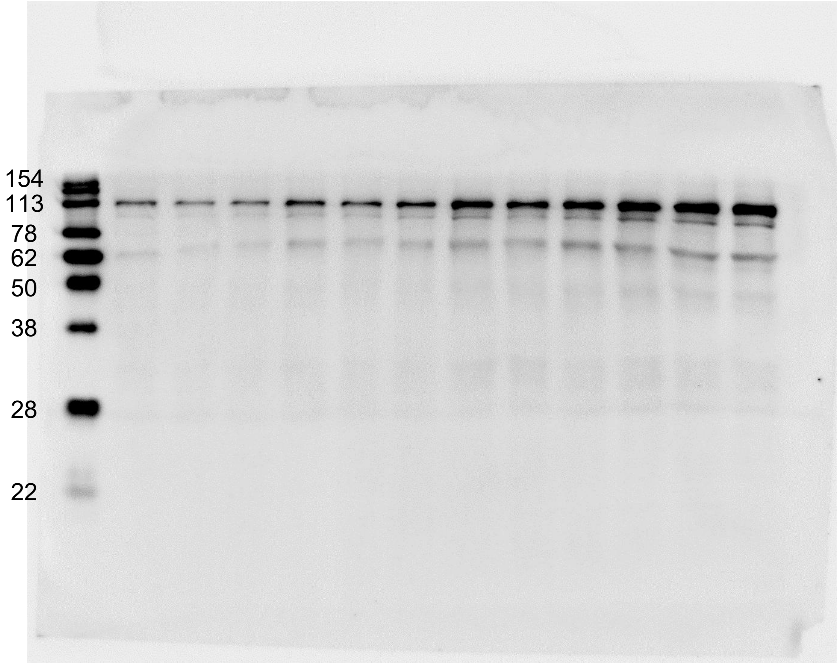

p-TAK1
75kDa

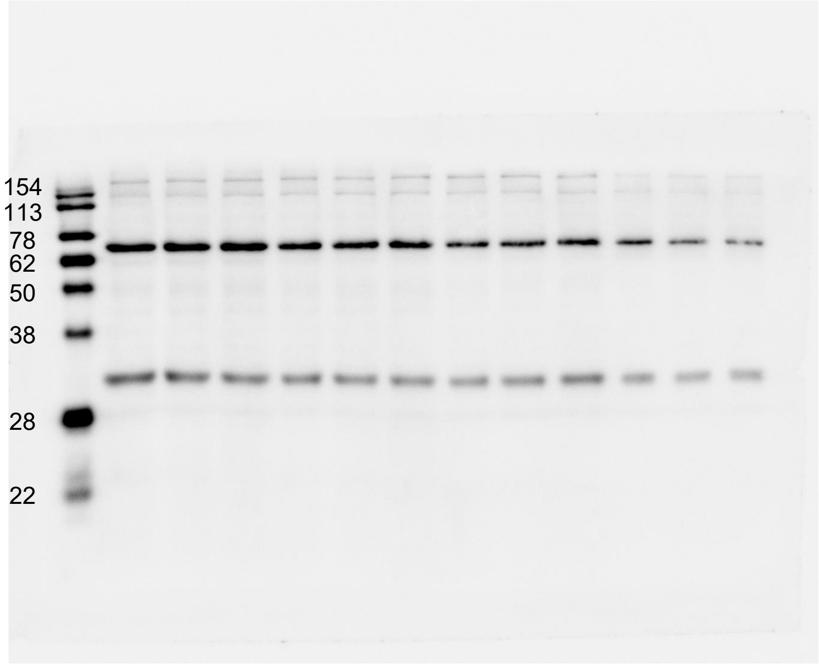

Figure 3G shows the whole blot after cutting membrane at molecular weight USP29 (104kDa), p-TAK1 (75kDa), TAK1 (75kDa) and β-actin (42kDa).

TAK1
75kDa

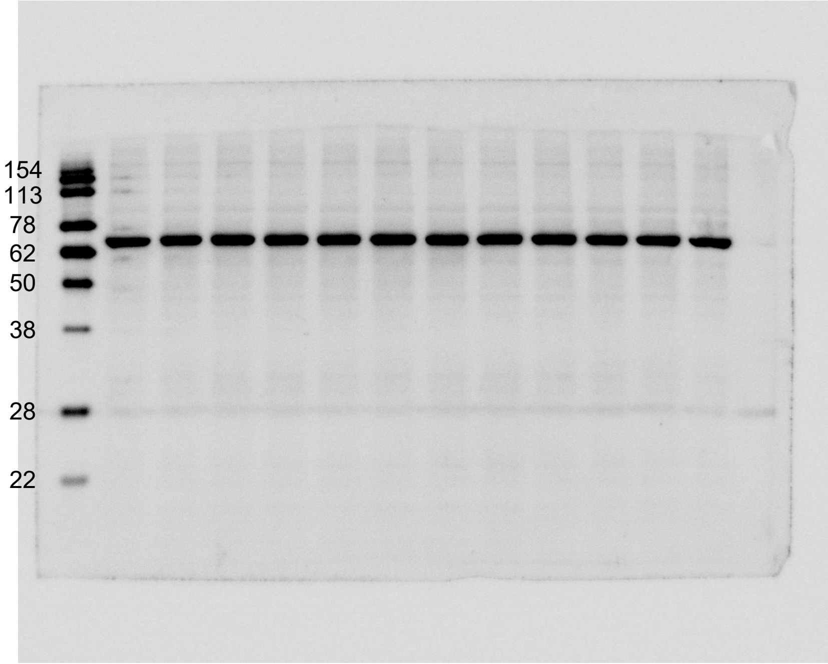

β-actin
42kDa

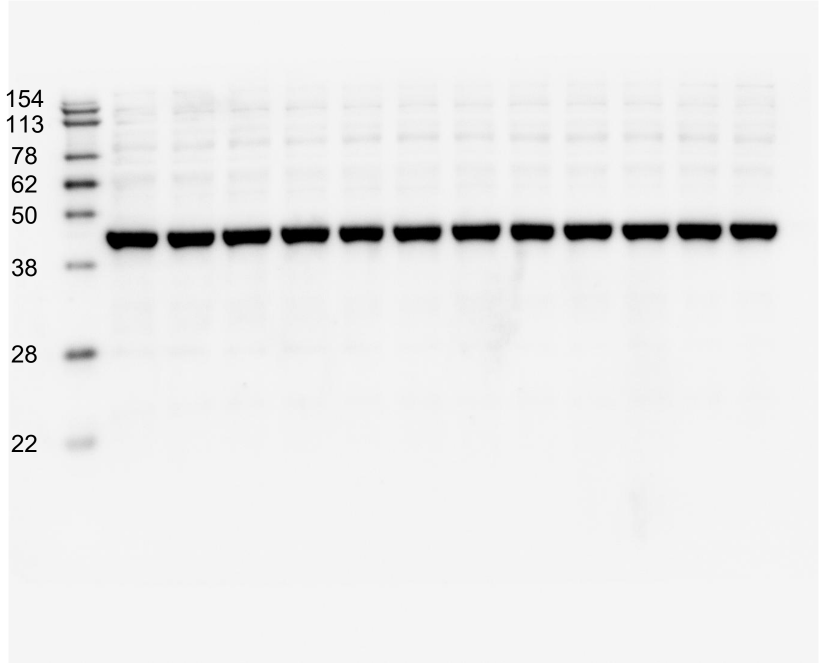

Figure 3H. Original western blot.

Figure 3H shows the whole blot after cutting membrane at molecular weight Flag and Myc.

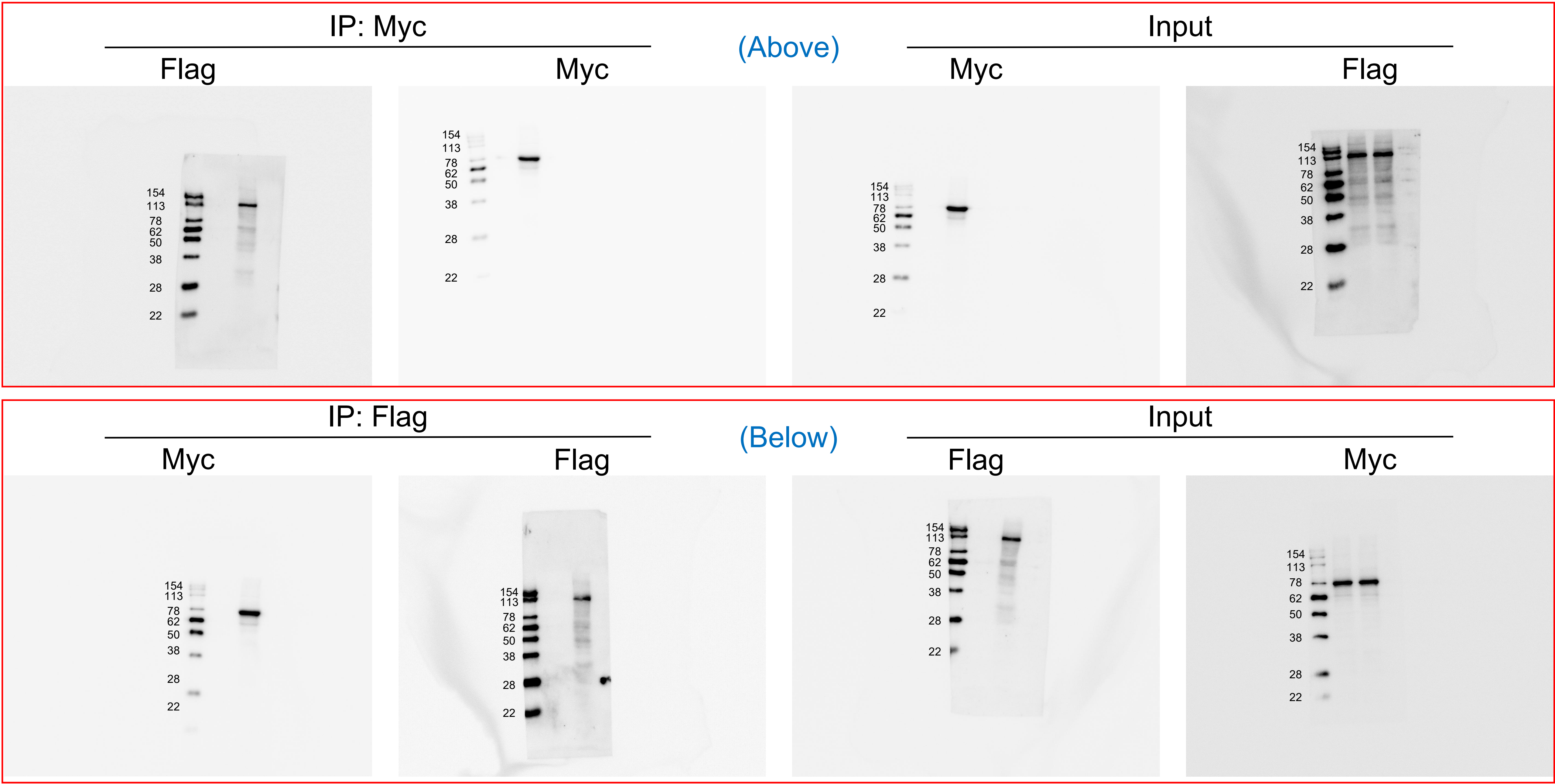

Figure 3I. Original western blot.

Figure 3I shows the whole blot after cutting membrane at molecular weight Flag and Myc.

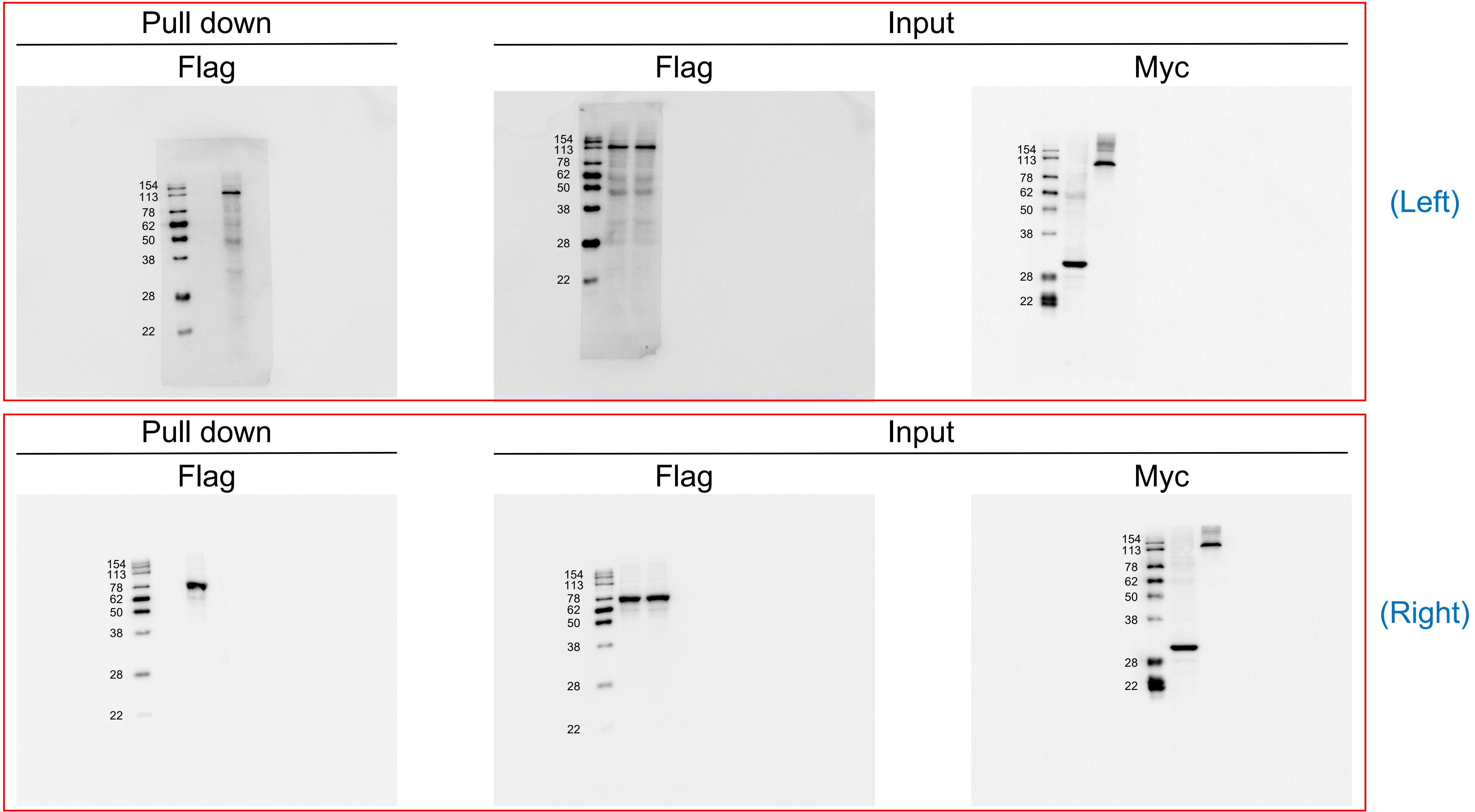

Figure 3J. Original western blot.

Figure 3J shows the whole blot after cutting membrane at molecular weight HA, Myc and Flag.

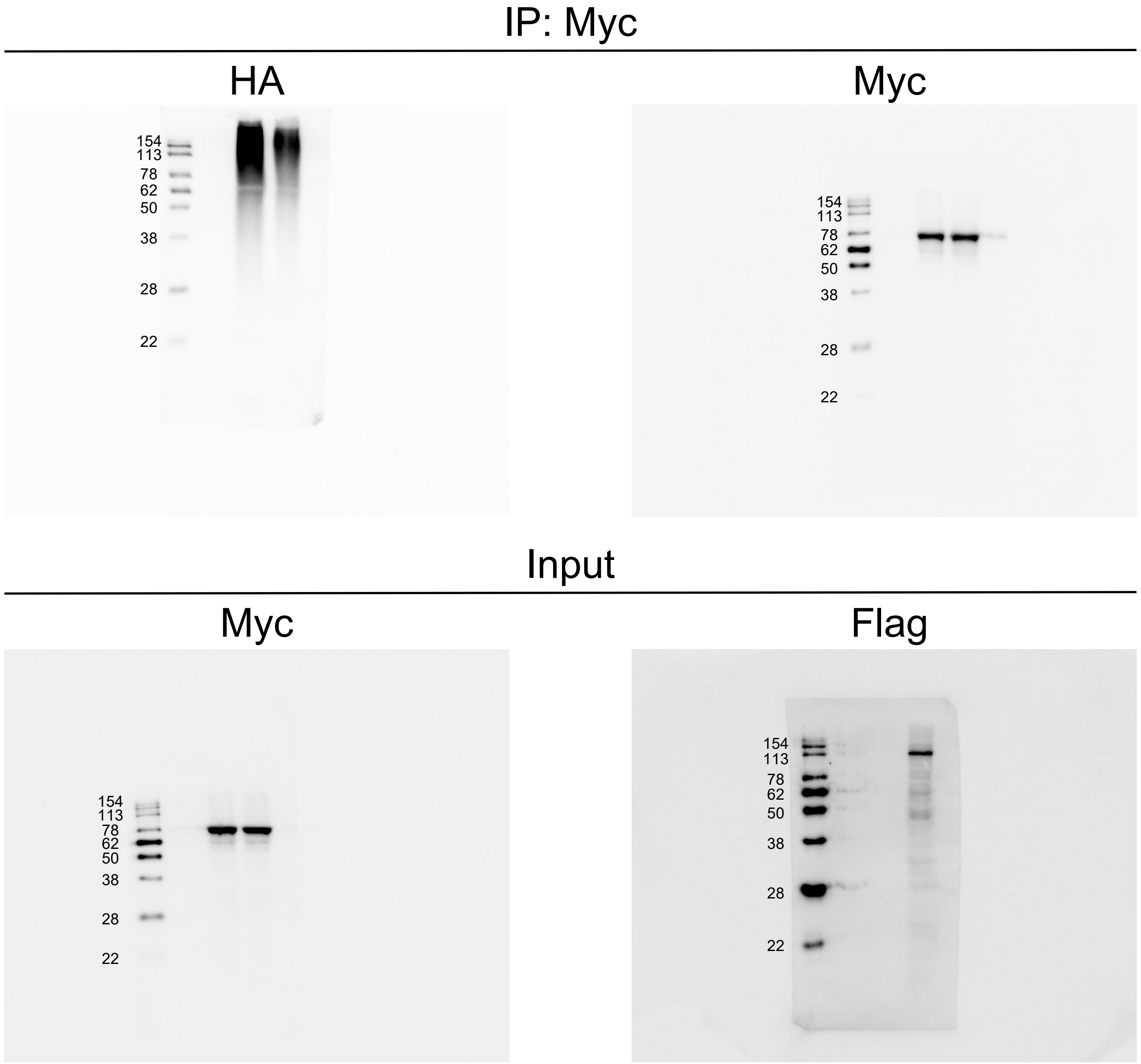

Figure 4A. Original western blot.

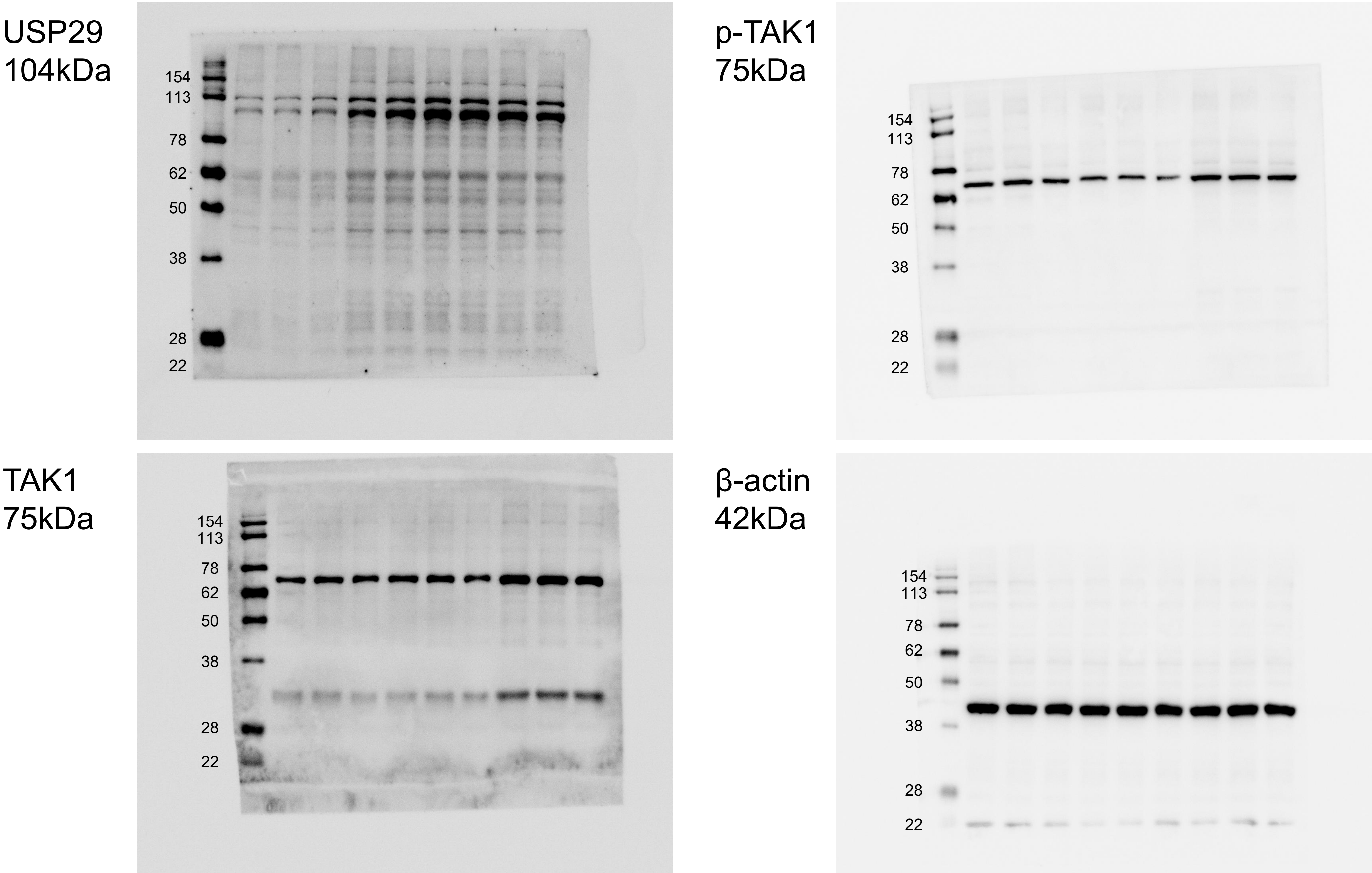

Figure 4A shows the whole blot after cutting membrane at molecular weight USP29 (104kDa), p-TAK1 (75kDa), TAK1 (75kDa) and β-actin (42kDa).

Figure 4D. Original western blot.

Figure 4D shows the whole blot after cutting membrane at molecular weight iNOS (131kDa), CD206 (166kDa) and β-actin (42kDa).

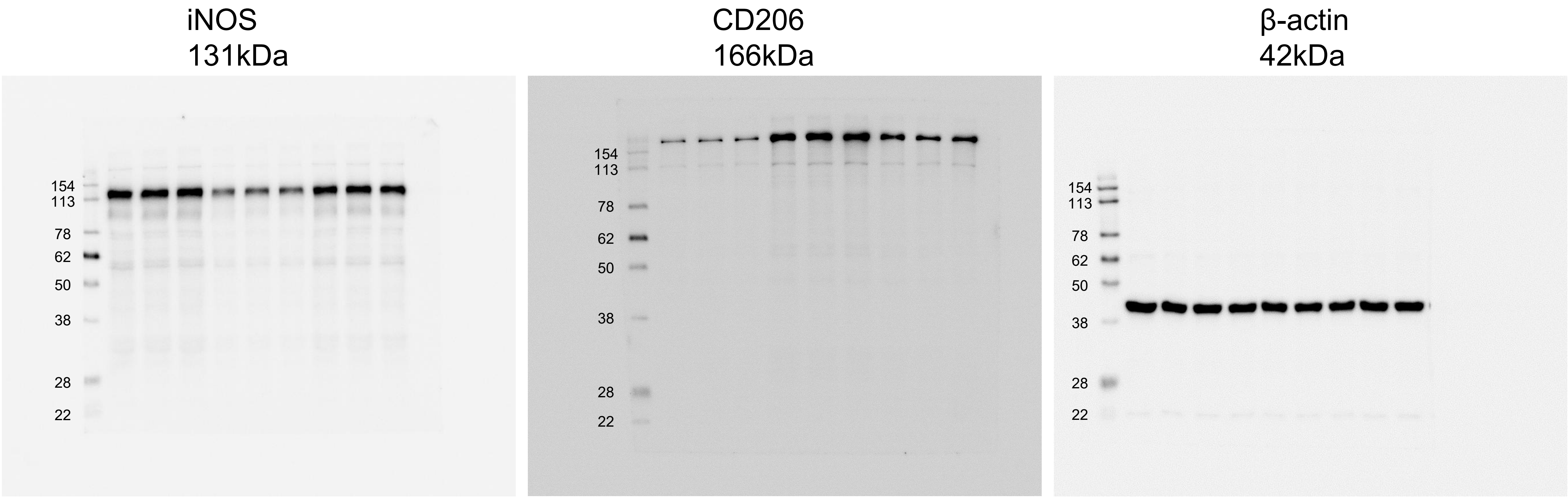

Figure 5B. Original western blot.

Figure 5B shows the whole blot after cutting membrane at molecular weight ELAVL1 (36kDa) and β-actin (42kDa).

ELAVL1
36kDa

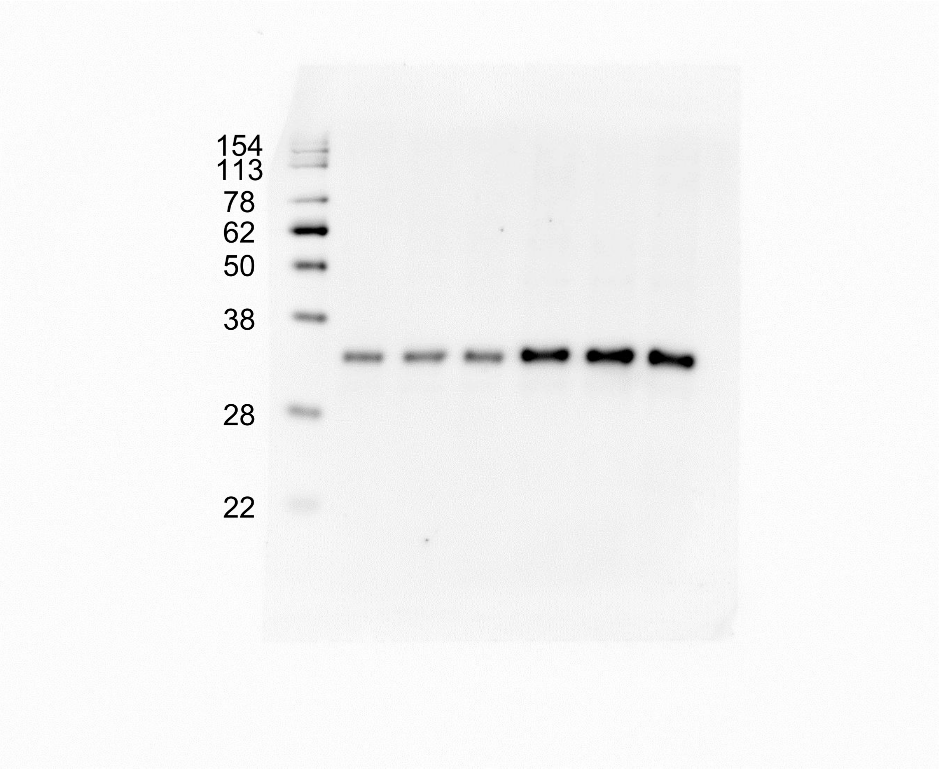

β-actin
42kDa

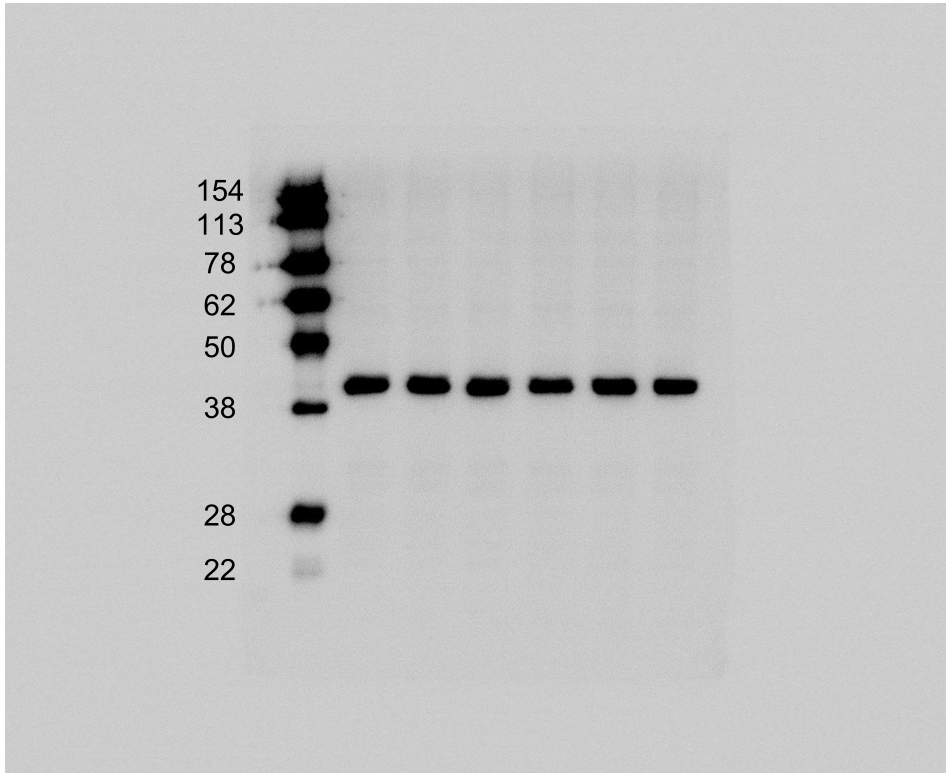

Figure 5C. Original western blot.

Figure 5C shows the whole blot after cutting membrane at molecular weight ELAVL1 (36kDa) and β-actin (42kDa).

ELAVL1
36kDa

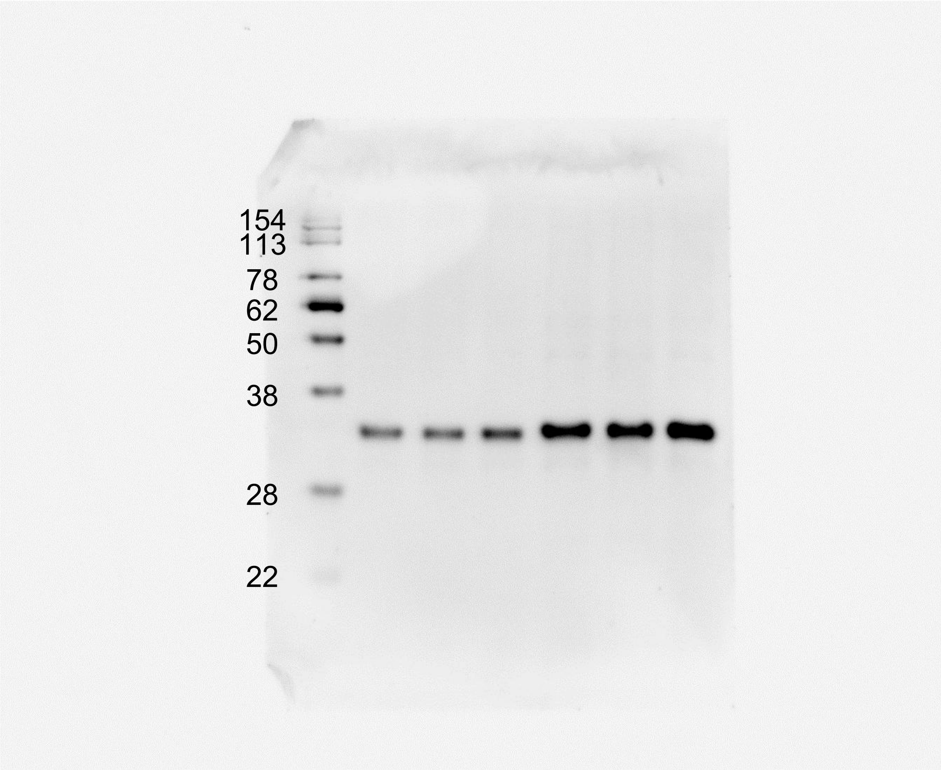

β-actin
42kDa

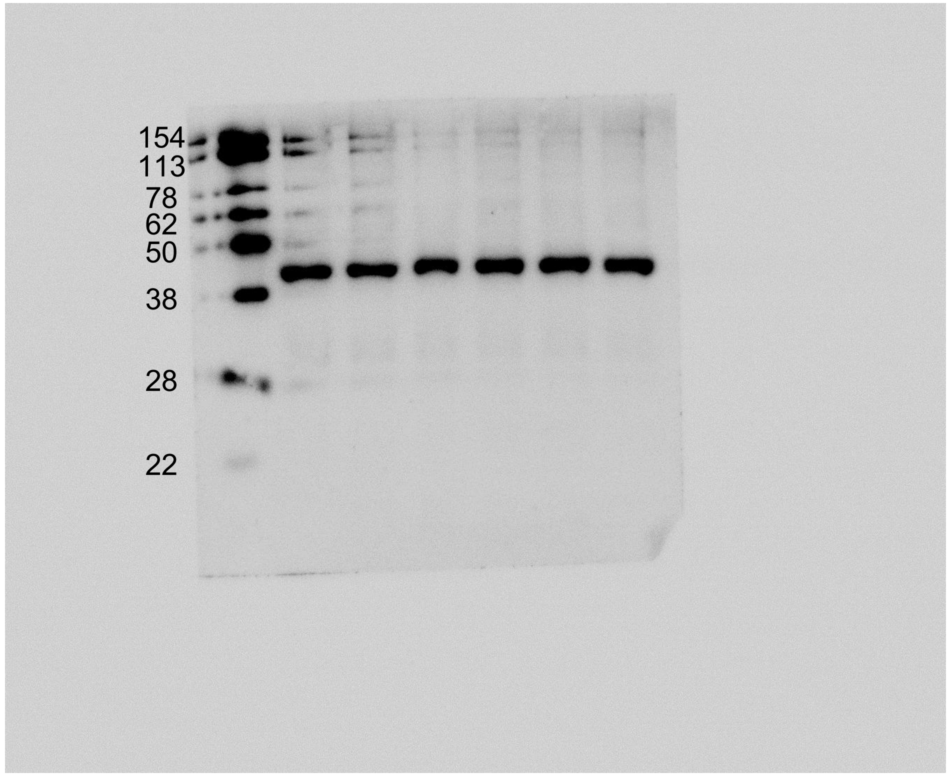

Figure 5F. Original western blot.

Figure 5F shows the whole blot after cutting membrane at molecular weight ELAVL1 (36kDa), USP29 (104kDa) and β-actin (42kDa).

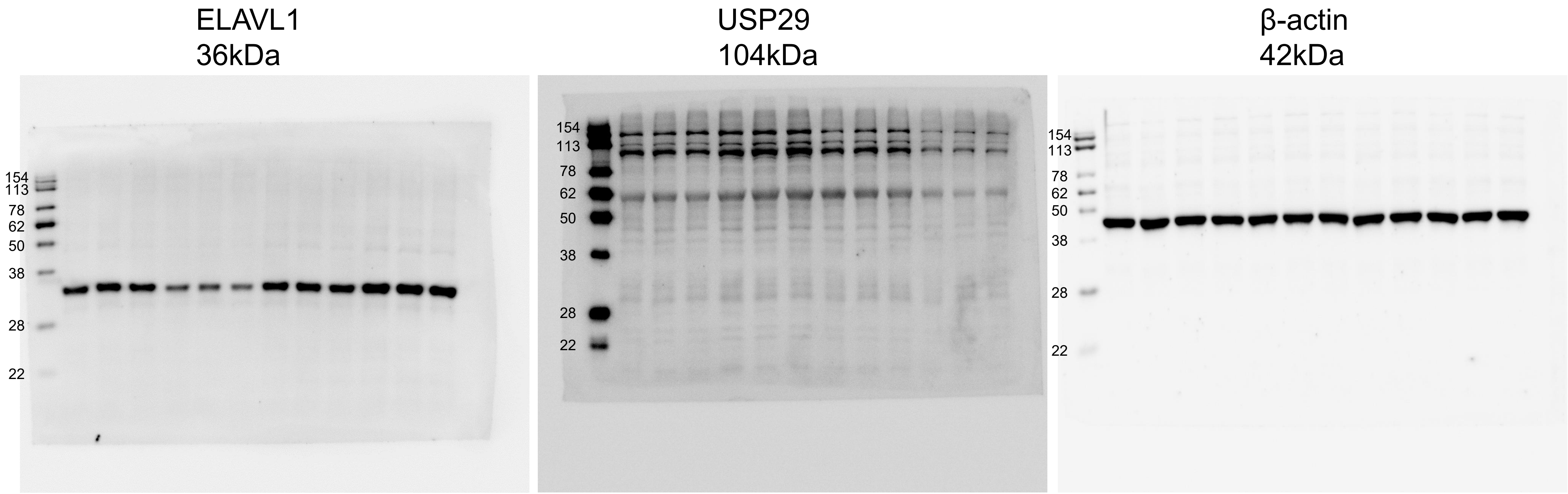

Figure 5H. Original western blot.

Figure 5H shows the whole blot after cutting membrane at molecular weight ELAVL1 (36kDa).

ELAVL1
36kDa

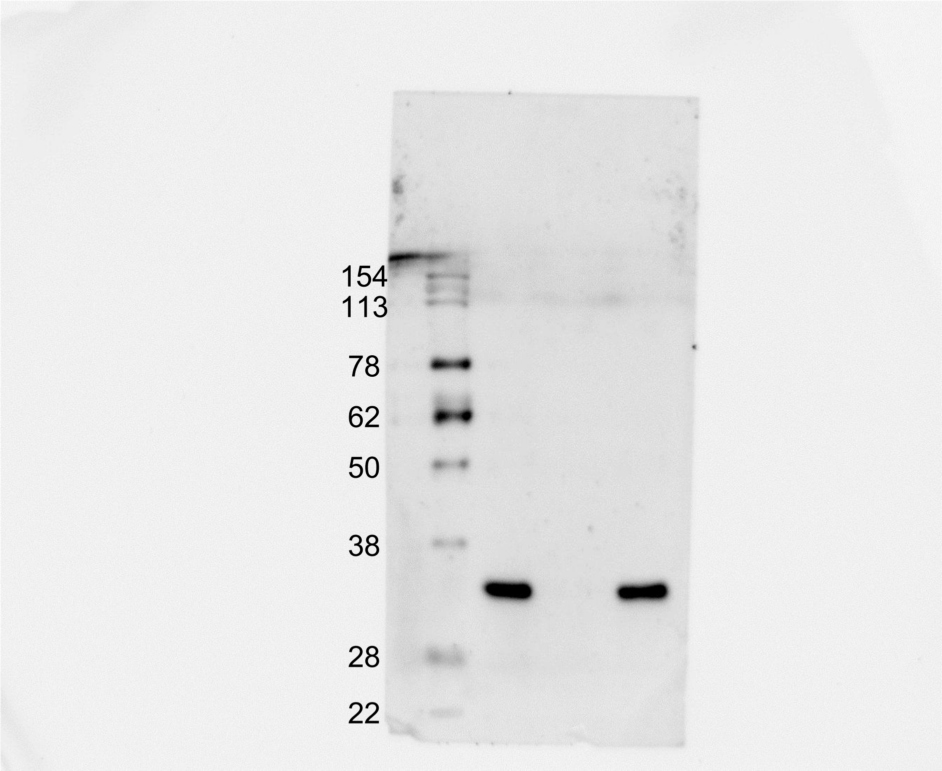

Figure 6B. Original western blot.

Figure 6B shows the whole blot after cutting membrane at molecular weight ELAVL1 (36kDa), USP29 (104kDa), p-TAK1 (75kDa), TAK1 (75kDa) and β-actin (42kDa).

ELAVL1, 36kDa

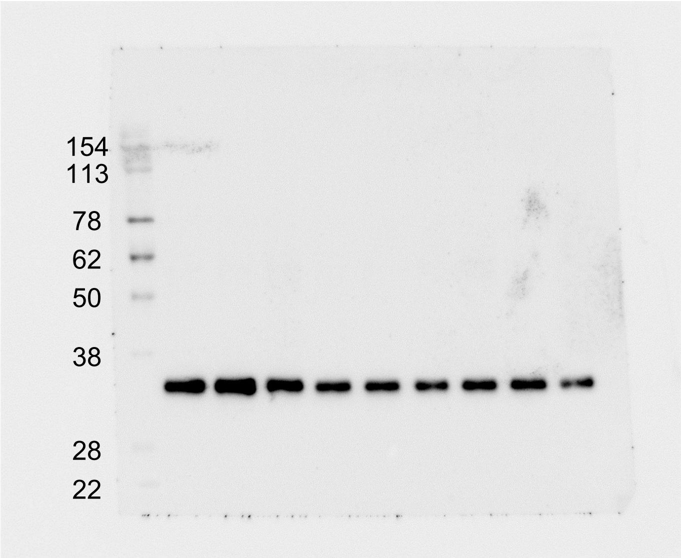

USP29, 104kDa

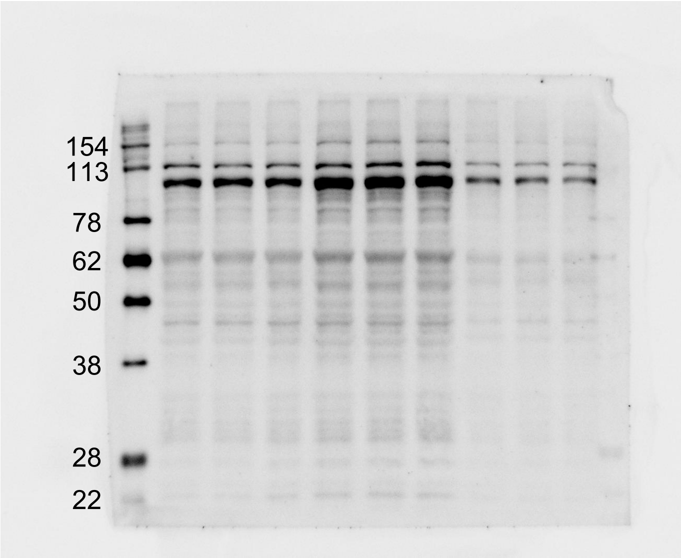

p-TAK1, 75kDa

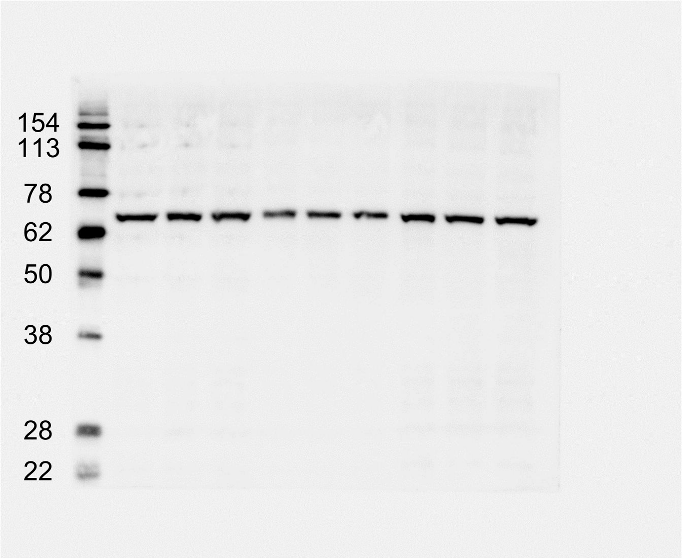

TAK1, 75kDa

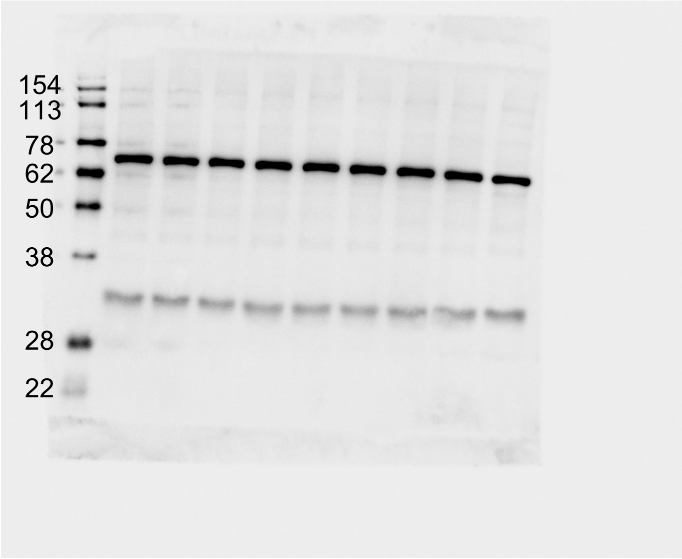

β-actin, 42kDa

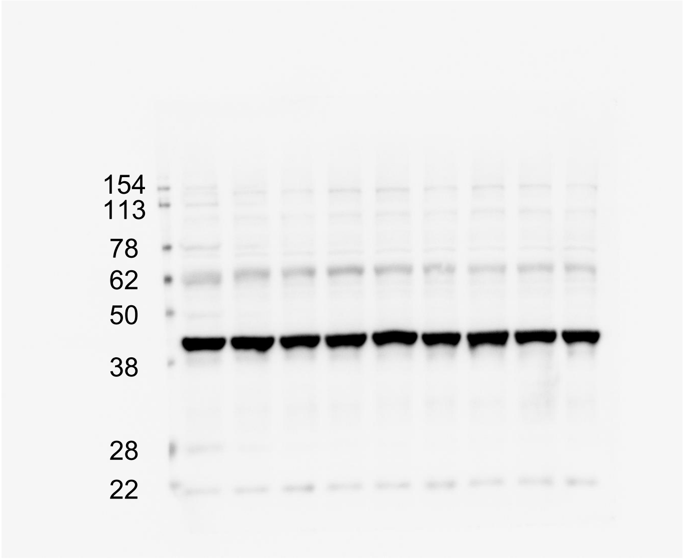

Figure 6E. Original western blot.

Figure 6E shows the whole blot after cutting membrane at molecular weight iNOS (131kDa), CD206 (166kDa) and β-actin (42kDa).

iNOS
131kDa

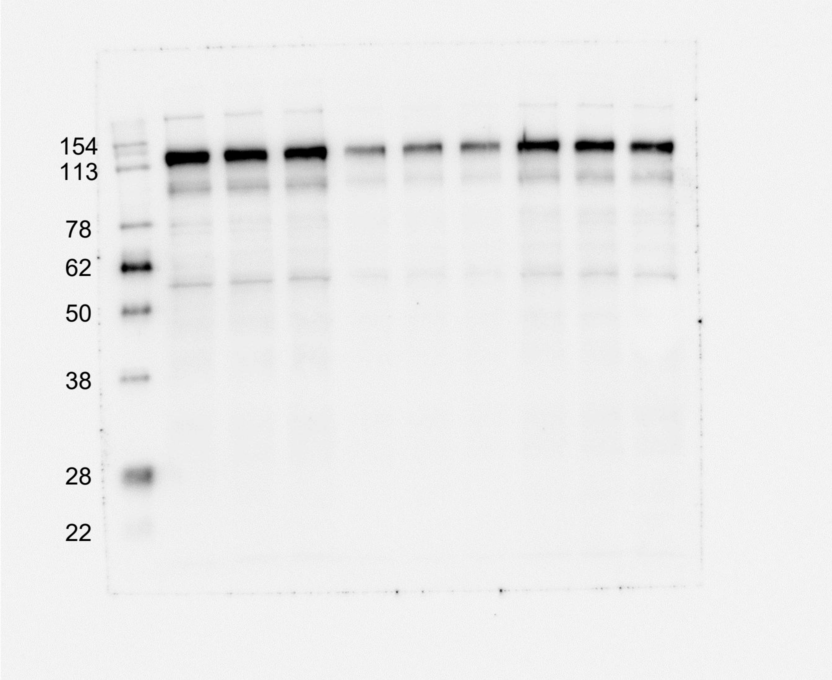

CD206
166kDa

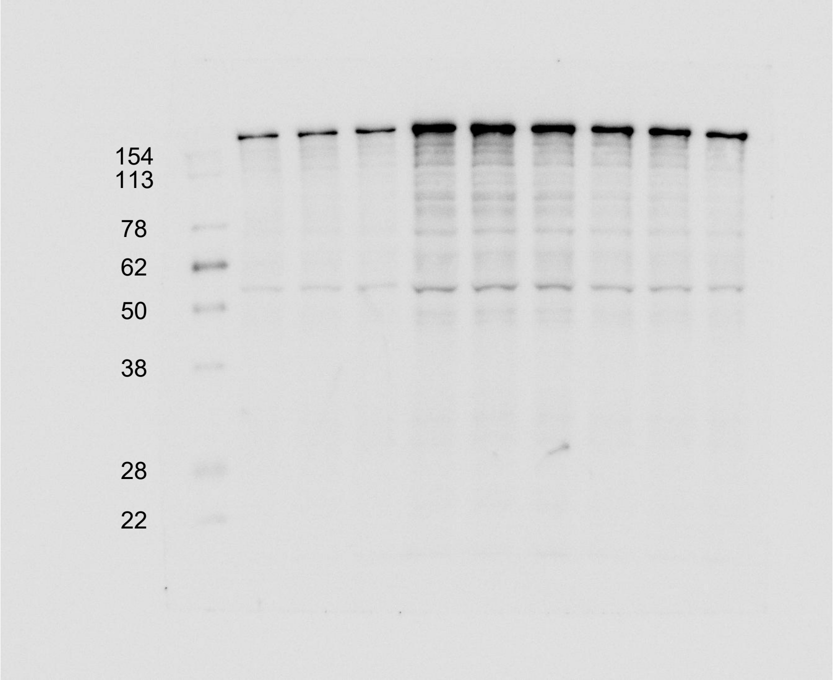

β-actin
42kDa

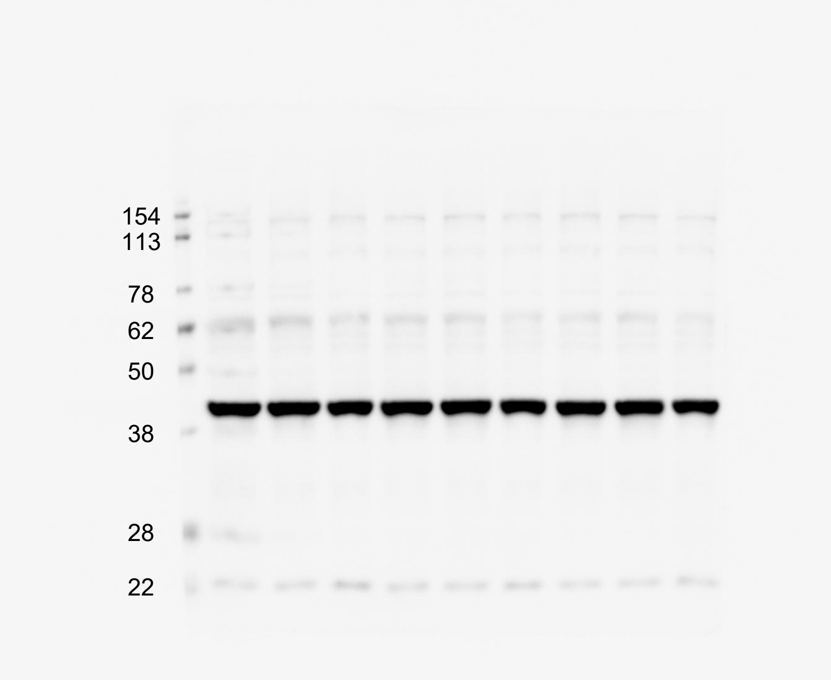

Figure 8B. Original western blot.

Figure 8B shows the whole blot after cutting membrane at molecular weight ELAVL1 (36kDa), USP29 (104kDa), p-TAK1 (75kDa), TAK1 (75kDa) and β-actin (42kDa).

ELAVL1, 36kDa

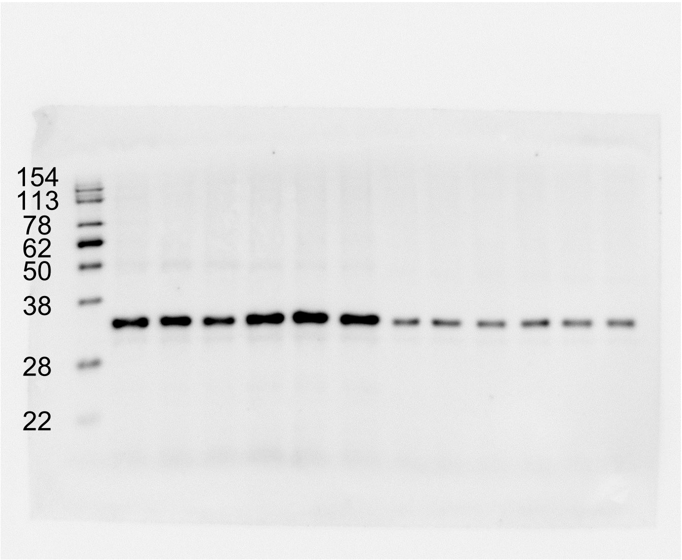

USP29, 104kDa

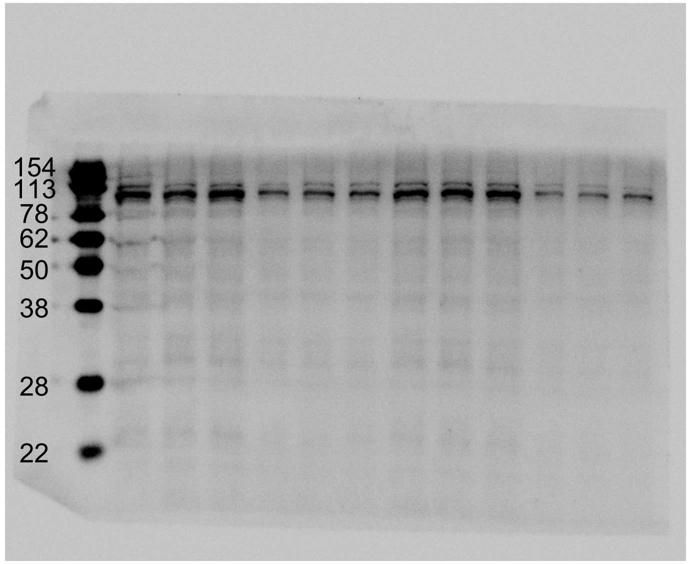

p-TAK1, 75kDa

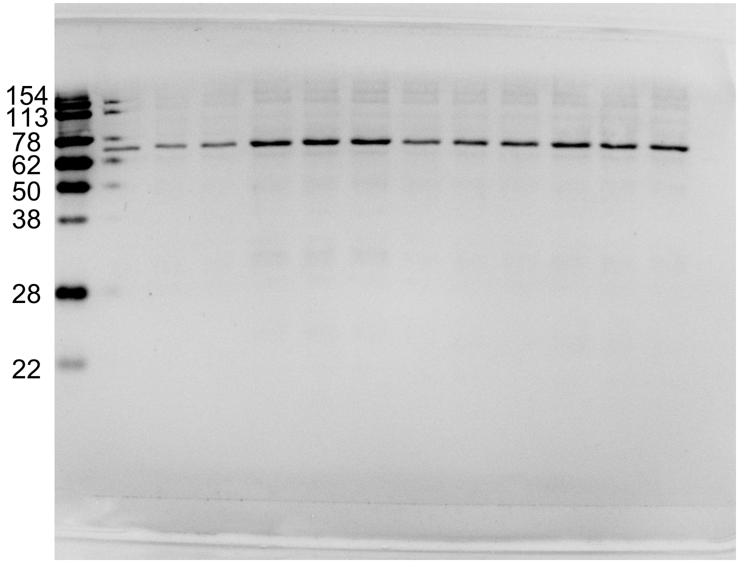

TAK1, 75kDa

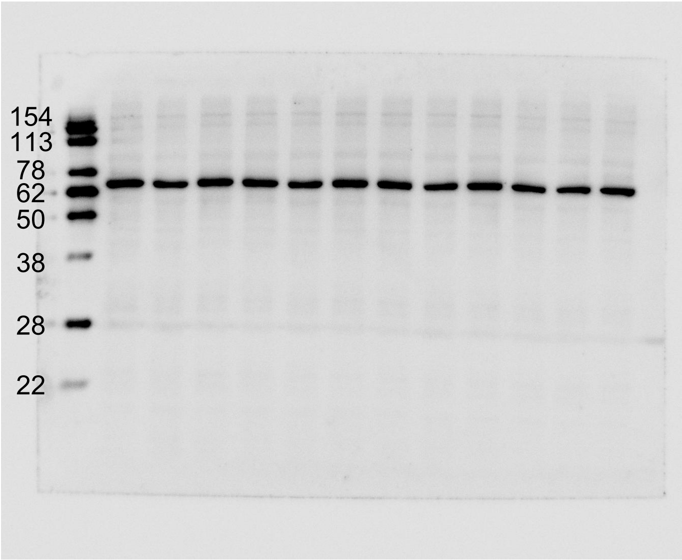

β-actin, 42kDa

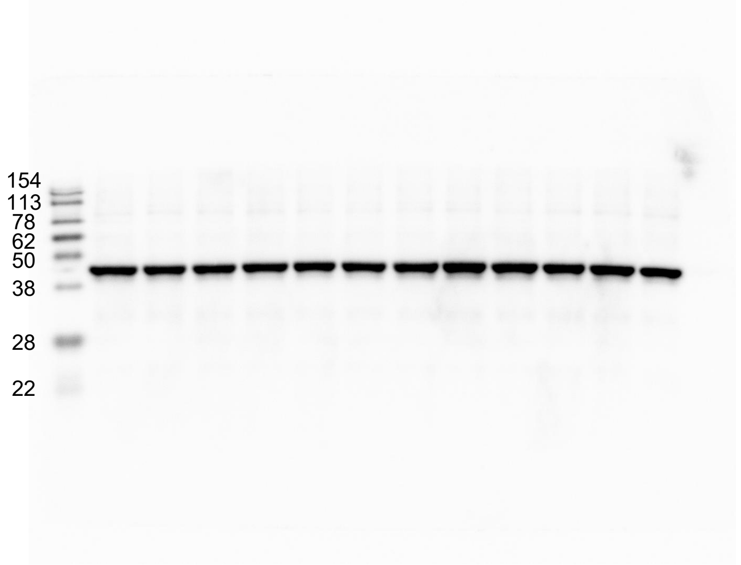

Figure S2B. Original western blot.

Figure S2B shows the whole blot after cutting membrane at molecular weight USP29 (104kDa) and β-actin (42kDa).

USP29
104kDa

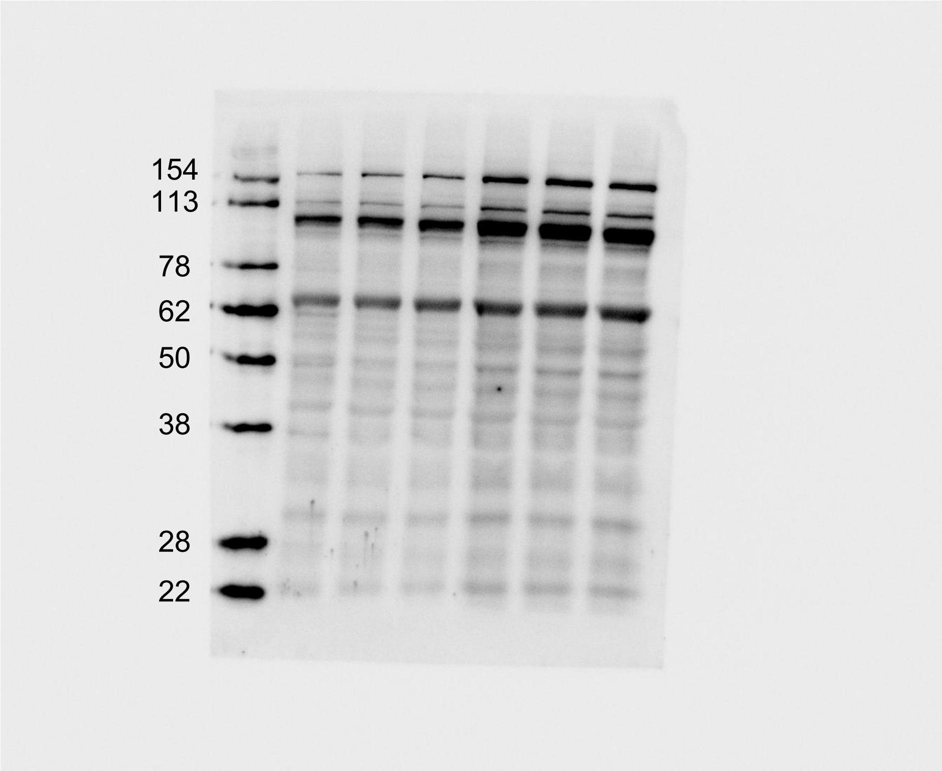

β-actin
42kDa

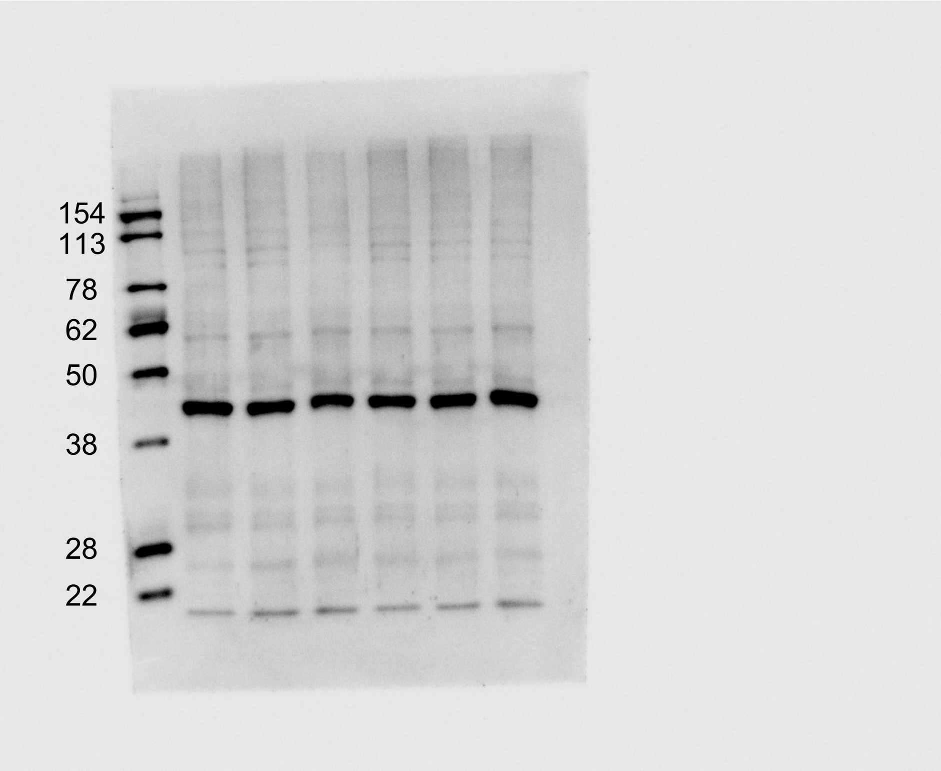

Figure S2E. Original western blot.

Figure S2E shows the whole blot after cutting membrane at molecular weight iNOS (131kDa), CD206 (166kDa) and β-actin (42kDa).

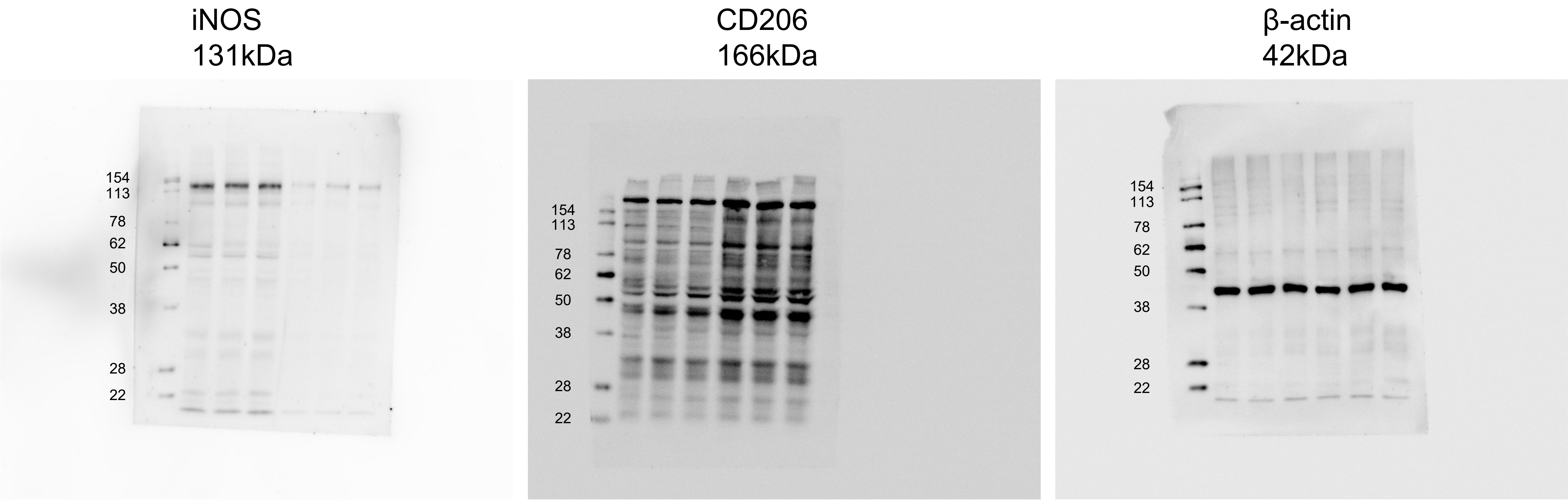

Figure S3A. Original western blot.

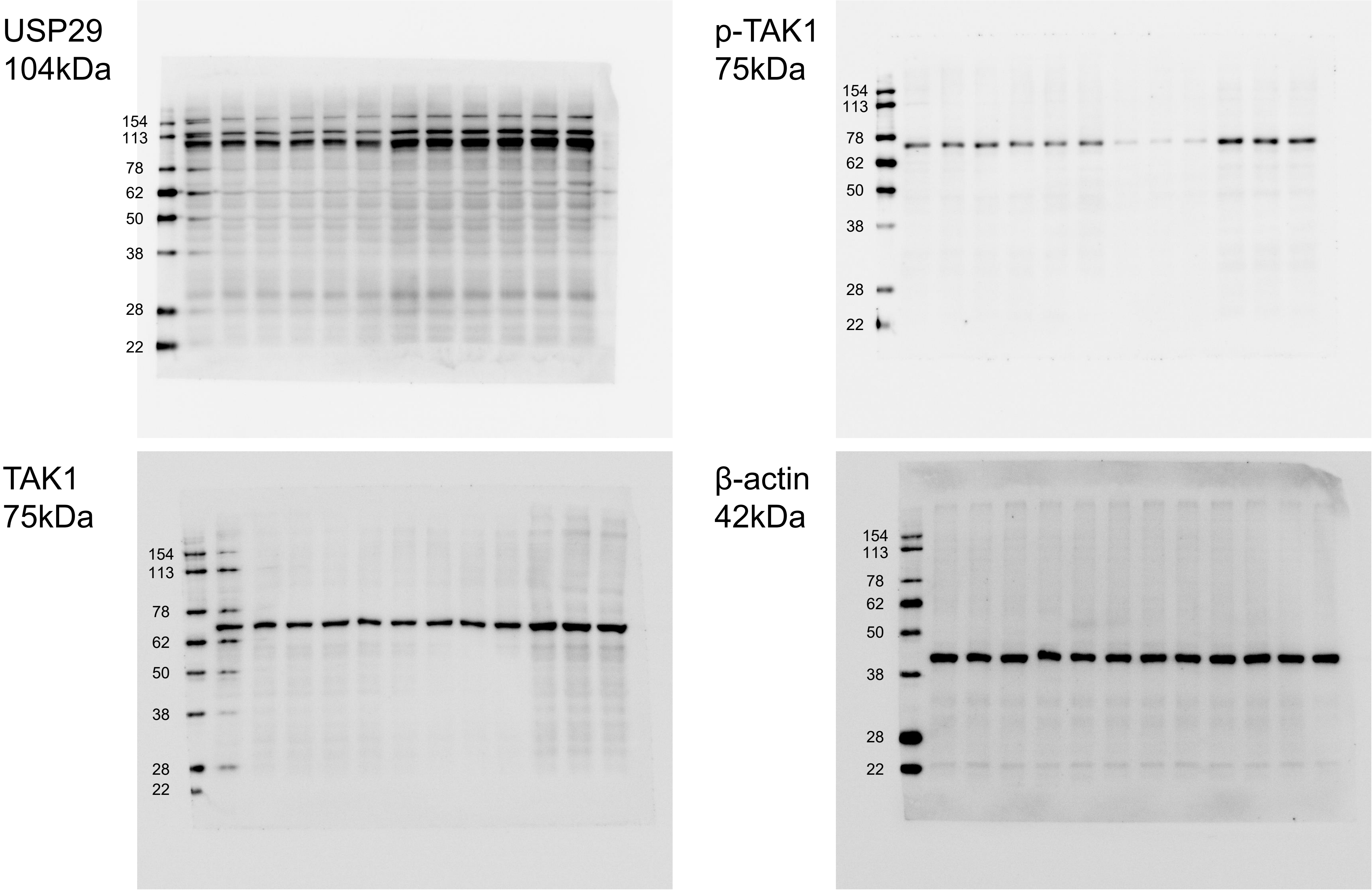

Figure S3A shows the whole blot after cutting membrane at molecular weight USP29 (104kDa), p-TAK1 (75kDa), TAK1 (75kDa) and β-actin (42kDa).

Figure S3C. Original western blot.

Figure S3C shows the whole blot after cutting membrane at molecular weight ELAVL1 (36kDa), USP29 (104kDa) and β-actin (42kDa).

ELAVL1
36kDa

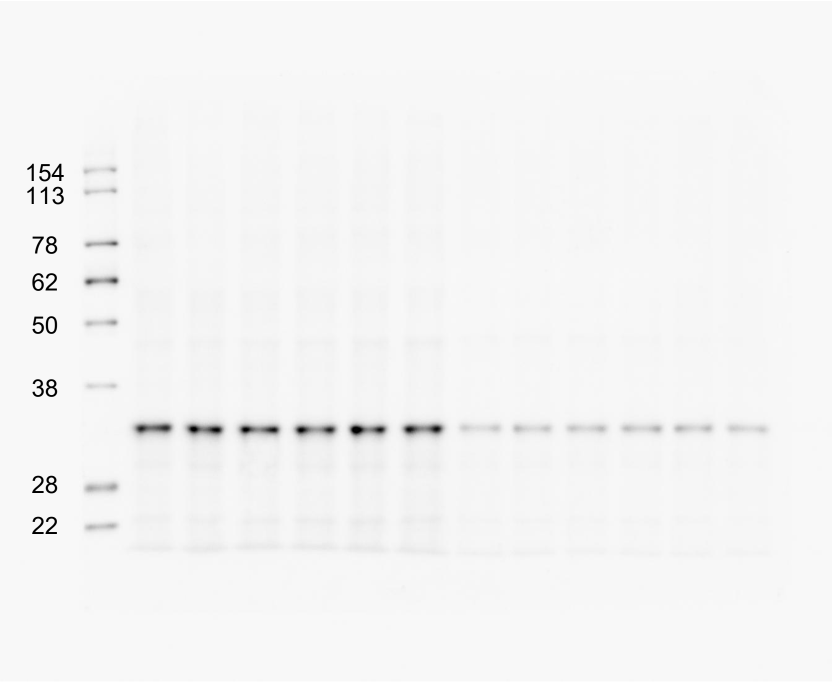

USP29
104kDa

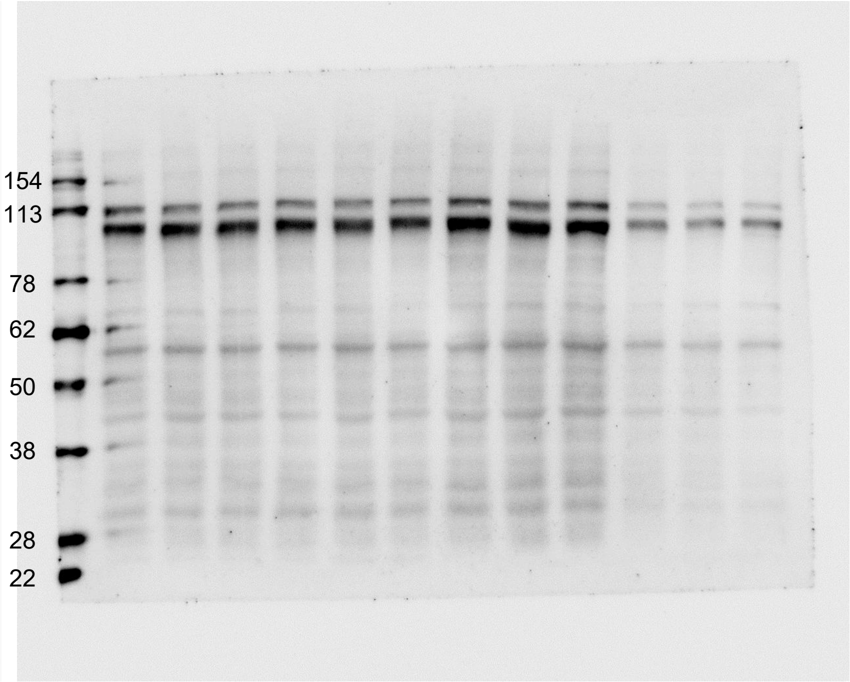

β-actin
42kDa

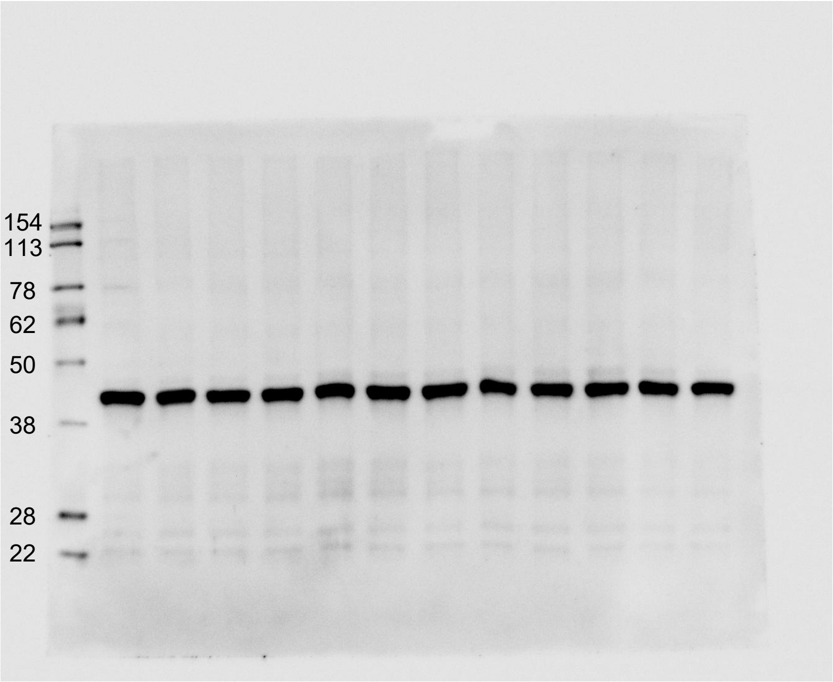

Supplement: Supplementary file 3 — Original figures [file 41420_2025_2604_MOESM3_ESM.pdf]
